# Non-uniform tropical forest responses to the ‘Columbian Exchange’ in the Neotropics and Asia-Pacific

**DOI:** 10.1038/s41559-021-01474-4

**Published:** 2021-06-10

**Authors:** Rebecca Hamilton, Jesse Wolfhagen, Noel Amano, Nicole Boivin, David Max Findley, José Iriarte, Jed O. Kaplan, Janelle Stevenson, Patrick Roberts

**Affiliations:** 1 Max Planck Institute for the Science of Human History, Jena, Germany; 2 School of Culture, History and Language, College of Asia and the Pacific, Australian National University, Canberra, Australian Capital Territory Australia; 3 Australian Research Council Centre of Excellence for Australian Biodiversity and Heritage, Australian National University, Canberra, Australian Capital Territory Australia; 4 School of Social Science, University of Queensland, Brisbane, Queensland Australia; 5 Department of Anthropology and Archaeology, University of Calgary, Calgary, Alberta Canada; 6 Department of Anthropology, National Museum of Natural History, Smithsonian Institution, Washington DC, USA; 7 Department of Archaeology, College of Humanities, University of Exeter, Exeter, UK; 8 Department of Earth Sciences, The University of Hong Kong, Hong Kong, China; 9 Archaeological Studies Programme, University of the Philippines, Quezon City, the Philippines

**Keywords:** Palaeoecology, Environmental impact, Archaeology

## Abstract

It has been suggested that Iberian arrival in the Americas in 1492 and subsequent dramatic depopulation led to forest regrowth that had global impacts on atmospheric CO_2_ concentrations and surface temperatures. Despite tropical forests representing the most important terrestrial carbon stock globally, systematic examination of historical afforestation in these habitats in the Neotropics is lacking. Additionally, there has been no assessment of similar depopulation–afforestation dynamics in other parts of the global tropics that were incorporated into the Spanish Empire. Here, we compile and semi-quantitatively analyse pollen records from the regions claimed by the Spanish in the Atlantic and Pacific to provide pan-tropical insights into European colonial impacts on forest dynamics. Our results suggest that periods of afforestation over the past millennium varied across space and time and depended on social, economic and biogeographic contexts. We argue that this reveals the unequal and divergent origins of the Anthropocene as a socio-political and biophysical process, highlighting the need for higher-resolution, targeted analyses to fully elucidate pre-colonial and colonial era human–tropical landscape interactions.

## Abstract (web summary)

The authors assess the impact of Spanish colonization on forest dynamics across the tropics, finding variable responses according to regional land use strategies, as well as other cultural, social and biophysical factors.

## Main

The term Anthropocene—used to describe a new epoch in which human activity has become the dominant influence on Earth systems—has been vigorously debated in the natural and social sciences^1–3^ since its popularization two decades ago^4^. Tropical forests, which cover only 14% of the Earth’s surface^5^ but contain 68% of the global living carbon stock^6^ and half of the Earth’s biodiversity^7^, are a central feature of this discussion^8^. Indeed, human-driven, habitat-scale reorganization of these systems (a conceivable scenario given contemporary climatic, fire and land use trajectories^9^) is thought to pose an “existential threat to civilization”^10^. The search for the beginnings of the Anthropocene has, in geological circles, centred on the identification of a single golden spike^11^. Attempts to track this critical transition initially focused on the onset of industrial fossil fuel burning in the eighteenth and nineteenth centuries^4^, but today concentrate on the Great Acceleration in the 1960s^11,12^. However, there have been growing calls in the social sciences to search for the origins of the Anthropocene as a long-term process that extends back into the pre-industrial era^2,13,14^, based on the premise that early agricultural processes substantially impacted atmospheric greenhouse gas concentrations, including CO_2_ levels^3^. This is particularly important within carbon-rich tropical forests where archaeological and palaeoecological research has revealed evidence for substantial human impacts on ecosystems, species distributions and soils over the past 45,000 years^15^. However, while there is increasing consensus that pre-industrial societies had large impacts on global ecosystems and biodiversity^16,17^, the exact scale and nature of anthropogenic alteration, particularly with respect to forest cover and CO_2_ concentrations, remains to be elucidated.

It has been proposed that contact between the so-called Old World and New World after 1492 ce as part of the expansion of the Spanish and Portuguese empires (termed the Columbian Exchange^18^) resulted in the radical reorganization of life on Earth without geological precedent^18,19^. Not only did Iberian colonizers bring new crops, animals and ways of using the land to the tropics^18^, they also introduced lethal diseases from Eurasia. The ensuing pandemics, alongside starvation and murder, wiped out up to 90% of Indigenous populations in the Americas, with the impact of their lack of immunity compounded by colonial policies focused on urban relocation and enslavement^20,21^. Earth system scientists have argued that this Great Dying, and the abandonment of traditional land use now known to have been extensive across the Neotropics, was so widespread that it led to dramatic forest regrowth^22,23^. The latest estimates suggest that subsequent afforestation captured 7.4 PgC (3.5 ppm CO_2_ equivalent) from the atmosphere, resulting in a CO_2_ level drop recognizable in ice cores by 1610 ce and driving global cooling seen in the form of the Little Ice Age (LIA)^21^. Although the global impacts of this regional signal have been promoted as a potential golden spike for the Anthropocene and the scale of the Great Dying is historically well documented (noting that specific estimates remain debated), direct evaluation of consequential vegetation change and overhaul of land management in carbon-rich tropical forests following Iberian colonization has been limited. Research of this nature in the Americas has often been locally constrained (for example, ref. ^24^), while larger-scale analyses have focused on the synthesis of charcoal records to reveal population and land use change, including a sustained period of reduced biomass burning after ~500 calibrated years before the present (cal yr bp)^21,25,26^. Assessment of ecosystem responses to these demographic and land use drivers has, to date, been qualitative, non-systematic and focused on a small number of datasets^21^. Homogenous, broad-scale ecological transitions in response to depopulation in the Neotropics thus remain more assumed than proven.

**Fig. 1:**
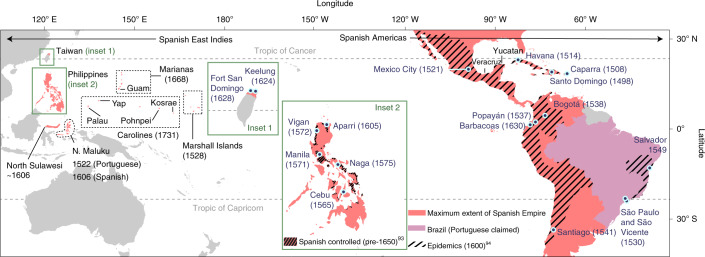
Extent of the Spanish Empire in the tropics. Map showing the maximum extent of the Spanish Empire in the tropics, including major colonial settlements and, for the Americas, regions impacted by major epidemics by 1600 ce. Dates in brackets indicate the approximate timing of colonization. Data on Spanish-controlled regions in the Philippines were from ref. ^93^. Data on epidemics were from ref. ^94^.

Although Alfred Crosby, who coined the term Columbian Exchange^18^, discussed impacts at a global scale, recent framings of this phenomenon on tropical ecosystems and their Earth systems feedbacks have been almost entirely limited to the Atlantic sphere^21^. This is despite the fact that, following their arrival in the Philippine archipelago in the sixteenth century, the Spanish Empire (including Portuguese-claimed regions incorporated into the Spanish Empire between 1580 and 1640 ce during the short-lived Iberian Union) united Madrid, Mexico City and Manila into the first truly pan-tropical biological, cultural and economic system^27^ (Fig. 1). Like their Neotropical counterparts, many societies living in Southeast Asia and parts of the Pacific had been part of extensive exchange systems that moved people, crops and ideas across vast areas^28^. Historical records and archaeology also show that the Spanish East Indies (including parts of Taiwan, Indonesia, Palau and Micronesia)—particularly those that were geographically isolated from Eurasia—witnessed large-scale (albeit staggered) disease spread^29^ and the introduction of additional novel domesticates^30^ between the 1500s and 1700s (contact dates shown in Fig. 1). The resultant infection rates, coupled with new forms of settlement organization and land use imposed by colonial states, led to major demographic disruption, with a population decrease estimated at 30–90% depending on pre-Iberian geography and demography (discussed for each region in Supplementary Text 2). It is thus plausible that associated shifts in traditional farming and forestry may have resulted in similar afforestation processes and, potentially, Earth systems feedbacks to those hypothesized for the Americas^29,31,32^. Yet, there has been no regional assessment of how Iberian arrival in the Asia-Pacific influenced ecosystems and Indigenous land use and whether any parallels can be drawn between the Pacific and Atlantic hemispheres^33^. Addressing this gap is important for unravelling the landscape legacy of Iberian colonization on a more global scale and, more broadly, as a starting point for assessing cross-continental, European colonial legacies within the tropics.

Here, we systematically compile and semi-quantitatively analyse pollen records from tropical regions of the Americas, Southeast Asia and the Pacific that became part of the first truly pan-tropical Empire—the Spanish Empire—between the 1500s and 1700s (Fig. 1). This permits direct, broad-scale insights into vegetation changes over the past 2,000 years—a time frame that provides context for understanding the scale of forest response to shifting land use and climate dynamics in the period leading up to and following European colonization. In doing so, we take advantage of Neotoma^34^—a rich, open access palaeoecological database that permits consistent reclassification of previously published data—to yield broad-scale assessments of ecological change in the Neotropics through time. We also compile pollen, phytolith and charcoal data from available palaeoenvironmental records in the Spanish East Indies to determine how tropical forests in the Asia-Pacific region responded to land use change associated with a decrease in the Indigenous population and Iberian colonization. We test the degree to which a uniform, pan-tropical Anthropocene process is visible following European colonization and assess how interplays of physical and human geography may complicate, or even overprint, this signal in ecosystem dynamics. We seek to provide a major advance on existing work and provide a framework for exploring how the concept of the Anthropocene can be more successfully applied as a tool for discerning the longevity, imbalances and variability of human–Earth system impacts over the past 2,000 years, providing more pragmatic perspectives for ongoing policy and conservation^35^.

## Results

### Neotropics

**Fig. 2:**
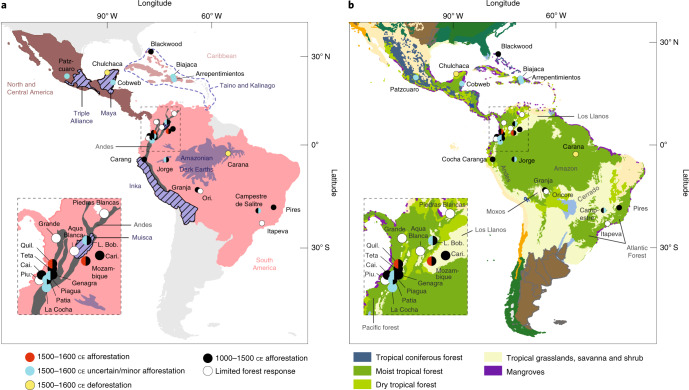
Locations of Neotropical pollen records. **a**,**b**, Locations of the Neotropical pollen records (points) included in this analysis shown relative to major pre-Iberian and Iberian era colonial geopolitical units (**a**) and ecoregions (**b)**^36^. In **a**, the red shading corresponds to the tropical Spanish Americas, whereas the blue shading and font represent selected pre-Iberian cultural zones. In **b**, any shading not defined in the key represents temperate/xeric biomes. The colour of each point corresponds to the assessed afforestation response of each record before (1000–1500 ce) and after (1500–1600 ce) Iberian contact. Record names are shown in black. Ecoregion names are shown in grey. In both panels, the insets show a magnified view of the Andes area highlighted by a dashed box in the main map. Terrestrial ecoregion data in **b** partially reproduced with permission from WWF.

The distribution of the 28 Neotropical sites included in our analysis of the Spanish Americas shows a spatial bias to the Andes and coastal regions and lacks data for regions known to be populous in the fifteenth century, including territory occupied by the Triple Alliance (or Aztec Empire) (Fig. 2). Nevertheless, there is generally at least one record for each of the major cultural sub-regions that have previously been defined within the Neotropics^21^ (Fig. 2a). This doubles the number of records assessed in the most recent attempt to gauge forest response to the Great Dying in the Americas^21^, thereby representing a major advance on previous assessments of post-colonial Neotropical vegetation change. Half of the analysed sites are located in moist tropical forest, five occur within dry tropical forest, three in tropical coniferous forest and six in tropical/montane savanna settings^36^. Given the poorly resolved age–depth models and sampling resolution of many of the datasets used in this analysis (discussed for each record in Supplementary Text 3), many afforestation responses after Iberian contact were classified with a degree of uncertainty (see Methods and Fig. 2). This factor has not been accounted for in recent attempts to use pollen data to assert early colonial era afforestation in the Spanish Americas^21^.

**Fig. 3:**
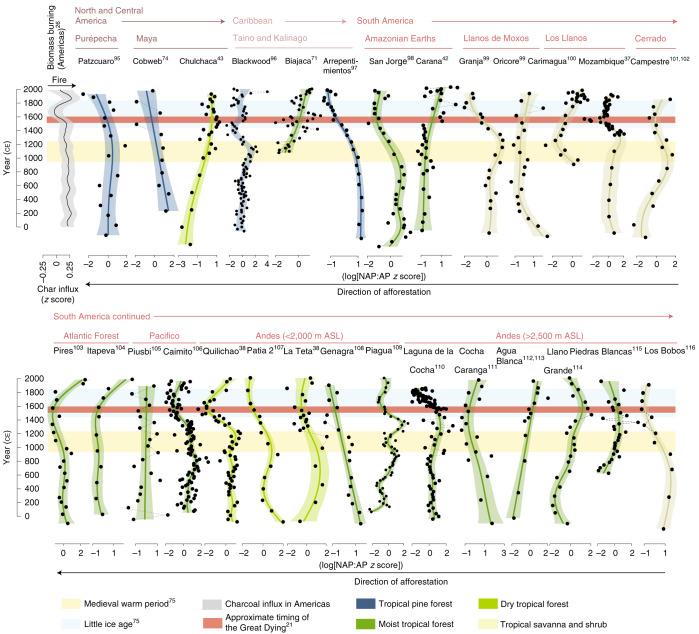
Neotropical non-arboreal to arboreal pollen ratios. Non-arboreal to arboreal (NAP:AP) pollen ratios (point data) overlain with GAMs (shading) over the past 2,000 years for 28 pollen records assessed from the Spanish Americas. The data are grouped according to broad geopolitical zones (Fig. 2a) and the shading of the GAMs corresponds to the contemporary biome in which each record currently occurs (Fig. 2b)^36^. The orange, blue and red horizontal bars represent the timings of the MWP^75^, LIA^75^ and Great Dying^21^, respectively. A composite biomass burning curve for the Americas, reproduced from ref. ^26^ and expressed as 150-year, LOWESS-smoothed, *z* scores of the transformed charcoal influx, is included (greyscale curve; top left) to provide context for the region-wide shift in the fire regimen over the past 2,000 years. The data used to create the remaining plots were from refs. ^37,38,42,43,71,74,95–116^. ASL, above sea level.

**Extended Data Fig. 1:**
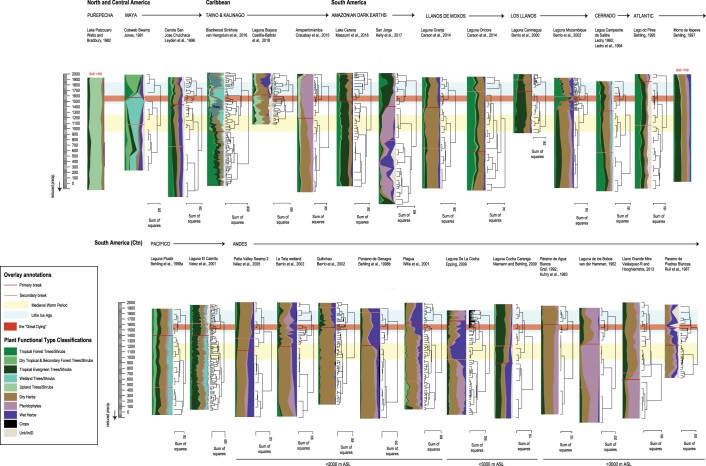
Changes in major plant functional groups & cluster analysis of Neotropical pollen records. Stratigraphic plot of Neotropical pollen records showing changes in major plant functional groups & cluster analysis of these data.

Eleven of the 28 records show a degree of afforestation between 1500 and 1600 ce (Fig. 2), consistent with prevailing theories of forest regrowth following European arrival^21^. This signal is classified with a higher degree of certainty (that is, it is clearly reflected in the generalized additive model (GAM) curvature and in the plant functional grouping (Extended Data Fig. 1)) for two of the 11 sites, which are located within the Los Llanos tropical savanna (Laguna Mozambique^37^) and Andean valley dry forests (Quilichao basin^38^) (Figs. 2b and 3, Extended Data Fig. 1). Fifteen sites indicate afforestation in the years preceding Spanish arrival (1000–1500 CE) (Figs. 2 and 3), probably linked to the onset of broadly wetter climate conditions over much of the Neotropics during this time period (Supplementary Text 1)^39,40^. Seven of these records occur within the Andes, including all five sites located in or immediately proximal to dry valley forest ecoregions (<2,000 m above sea level). This spatial response bias likely reflects the cooler, wetter conditions associated with the LIA in the Andes, coupled with the sensitivity of seasonally dry and Neotropical montane forests to changing climate drivers^41^. There is no clear spatial or cultural relationship among the remaining eight records that reflect pre-Iberian afforestation. However, five of these sites occur within biomes other than moist tropical forest (four within tropical savanna and scrubland and one in coniferous tropical forest^36^). This implies heightened climatic sensitivity of non-rainforest biomes that lie closer to precipitation thresholds and/or that changes in the availability of resources under a changing climate regimen within these habitat types encouraged social restructuring. Two of the 28 records indicate forest opening between 1500 and 1600 ce: Lake Caranã^42^ (a long-cultivated tropical forest site in the Amazon) and Cenote San Jose Chulchaca^43^ (a site occurring on the boundary of the Maya lowlands within dry tropical forest).

We also explored changes in vegetation in these records over the past 150 years to provide some comparison of pre-industrial and industrial era environmental changes, although these results should be approached cautiously given the lack of sampling and age–depth resolution in the younger portion of several of the records included in this analysis (and hence lack of signal detection in the GAM (Fig. 3)). There appears to be a clear signal of deforestation in sites from the Caribbean (2/3), Atlantic Forests (2/2), Cerrado (1/1), Los Llanos (2/2), lowland Andes (2/5) and Amazonian rainforest (1/2) after ~1850 ce (Fig. 3).

### Spanish East Indies

**Fig. 4:**
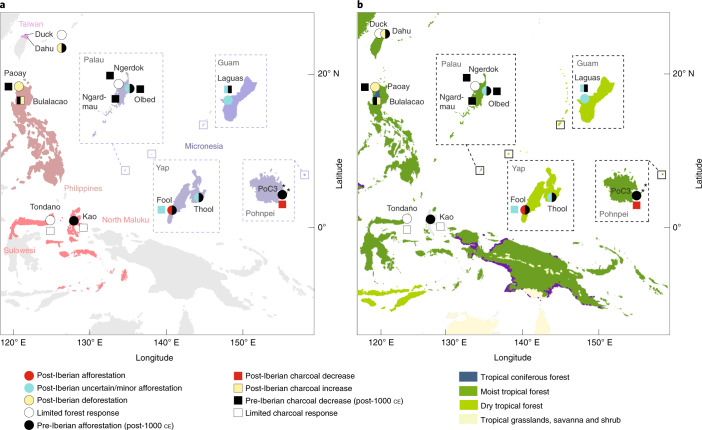
Locations of the palaeoecological sites in the Asia Pacific region. **a**,**b**, Locations of the palaeoecological sites in the Asia Pacific region included in the present analysis, shown relative to geopolitical units **(a**) and ecoregion (**b**)^36^. In **a**, the coloured shading corresponds to the four biogeographic zones in the former Spanish East Indies. In **a** and **b**, circular points represent pollen records, squares represent charcoal records and the asterisk represents the single phytolith record. The colour of each circle (pollen/phytolith records) or square (charcoal records) corresponds to the assessed afforestation or fire response before and after Iberian-introduced epidemics, the timings of which were variable across the region and are discussed in Supplementary Text 2. Record names are shown in black, whereas select Micronesian island names are shown in grey. Terrestrial ecoregion data in **b** partially reproduced with permission from WWF.

With the exception of the Marshall Islands, our dataset includes at least one island from each of the major tropical archipelagos directly impacted by Iberian imperialism in the Asia-Pacific region (Fig. 4). There is, however, a scarcity of data from the Philippines—the centre of (and largest archipelago within) what was known as the Spanish East Indies—which is only represented by a single charcoal record and a single pollen record for the colonial period (both from the island of Luzon). Importantly, historical records suggest that all of the assessed regions in the Spanish East Indies experienced a degree of Indigenous population mortality following European contact between the 1500s and 1700s (the timing and extent of which is reviewed in Supplementary Text 2). Together, the 21 palaeoenvironmental records from 13 sites encompass the major tropical biogeographical zones in the region (Taiwan, the Philippines, Wallacea and Micronesia) (Fig. 4). Nine of the analysed sites occur in moist tropical forest, three occur within seasonally dry tropical forest and one occurs in tropical coniferous forest^36^ (Fig. 4b).

**Fig. 5:**
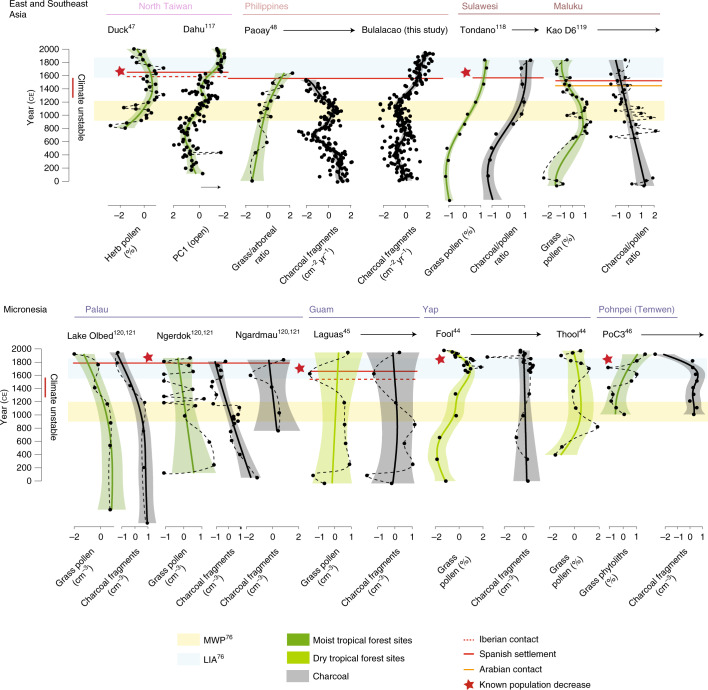
Afforestation proxies and charcoal curves from the Asia Pacific region. Afforestation proxies (pollen and phytolith records) and charcoal curves (point data) overlain with GAMs (coloured shading for pollen/phytolith data and black shading for charcoal data) over the past 2,000 years for the 21 records assessed from the Spanish East Indies. The data are grouped according to broad geopolitical zones (Fig. 4a) and the shading of the GAM for the pollen/phytolith data corresponds to the contemporary biome in which each record currently occurs (Fig. 4b)^36^. The orange and blue horizontal bars represent the occurrences of the MWP and LIA, respectively, in the Asia-Pacific^76^. The timing of Iberian contact and known population decrease is individually annotated for each region. The data used to create the plots were from refs. ^44–48,117–121^, as well as the present study.

There is evidence for afforestation and decreased fire activity in four pollen/phytolith records and three charcoal records from Micronesia after archival references to a decrease in the Indigenous population (between ~50 and 90%; Supplementary Text 2). Most of these shifts have been classified with a degree of uncertainty (Fig. 4) due to the low sampling and chronological resolution of the datasets, meaning that this change is not captured in the GAMs produced for each record (Fig. 5 and Supplementary Text 3). All of the records showing afforestation in the years following European contact come from small islands (Yap (Fool and Thool swamps)^44^ and Guam (Laguas)^45^) and 75% of the records are located within seasonally dry tropical forest (Fig. 4b). Available charcoal data from these islands indicate that afforestation coincides with reduced or unchanged fire activity in the landscape (Fig. 5). Decreased fire activity after a known population decrease (50% between 1840 and 1900 ce) is also reflected in a charcoal record from Pohnpei^46^, although in this case there is no evidence of corresponding afforestation, possibly due to the simultaneous introduction of pigs to the island by the Spanish (see Supplementary Text 3).

None of the records from regions that regularly traded with mainland Eurasia before colonization (Minahasa, North Maluku, Luzon and North Taiwan)—which also appear to have relatively low post-colonization mortality rates (15–50%; Supplementary Text 2)—show an afforestation signal following Iberian contact. In fact, the records assessed from the Philippines and Taiwan suggest that colonization resulted in deforestation and increased fire disturbance, although this is not captured in the GAM (and thus not classified) for the Duck Pond site^47^ (Taiwan), while the Lake Paoay^48^ (Philippines) charcoal data do not include the Spanish colonial period (Fig. 4 and 5; see Supplementary Text 3 for details). Higher sampling resolution and better selection of sites relative to key European occupation zones, particularly for the Philippines, are required to investigate the consistency of this process over space.

Approximately half of the pollen/phytolith (6/11) and charcoal (6/10) records show afforestation and decreased fire in the landscape in the centuries before Iberian imperial influence. There is no consistent geographic or ecoregional pattern associated with this signal. This suggests that changes in forest cover after colonization were, in many cases, muted relative to those caused by land use or climatic factors before Iberian contact.

The lack of sampling resolution in the Asia-Pacific records, as well as the relatively late influence of Europeans on some of the islands (Palau (1800s), Yap and Pohnpei (1700s); Supplementary Text 2) means that it is not possible to tease out an industrial era signal of ecosystem change for this region from available palaeoecological data.

## Discussion

Over one-third of the palaeobotanical records from the Atlantic and Pacific realms indicate a degree of afforestation (including minor or uncertain forest recovery) following Iberian contact. This, in part, appears to support claims made in previous work that the documented decrease in Indigenous populations in the Americas following the introduction of foreign diseases and colonial policies led to a collapse of existing farming and food production systems and concomitant forest regrowth^21^. Furthermore, it provides the first evidence that this process was not exclusive to the Americas, but also occurred in the Spanish East Indies (although it should be noted that the timing of Iberian contact was staggered in the Asia-Pacific; see Supplementary Text 2). Nevertheless, the lack of consistency in this response across the entire spectrum of records studied indicates that variable land use strategies, as well as other cultural, social and biophysical factors, played a key role in the observed changes to vegetation and burning. For instance, documented Indigenous resistance to Iberian occupation (for example, in North Sulawesi^49^ and the West Caroline Islands^50^) appears to have resulted in a geographically isolated settlement and/or a protracted Iberian settlement process, thereby drawing out the spread of Eurasian pathogens and land use (discussed in Supplementary Text 2). A similar result may have also ensued from the geographic inaccessibility of, lack of Iberian interest in or socio-ecological resilience of certain regions, including the interior Amazon^51^ and Pacific coast rainforests^52^, the Llanos de Moxos^53^, Palau^54^, the Brazilian Cerrado^55^, the West Carolines^56^ and the highlands of Hispaniola^57^ and the Philippines^58^ (discussed in Supplementary Text 2). As a consequence, the demography of some of these less accessible sites after Iberian colonization may have been characterized by population replacement or migration rather than just an abrupt decrease in the population. Finally, it is important to point out that certain land use strategies adopted in the tropics (for example, the polyagricultural systems deployed in the eastern and southwestern Amazon Basin) may have actually sustained forest cover, thus challenging the assumption that afforestation in palaeoenvironmental records should be the only expected ecological signal of a decrease in the Indigenous population following colonization^42,59^.

Ecological and biogeographic factors may also have mediated forest resilience to human disturbance in both the pre-colonial and early colonial era. For instance, seasonal ecosystems within both the Atlantic and the Pacific appear more prone to Iberian-era afforestation, potentially reflecting their structural reliance on Indigenous land use practices, particularly swiddening^60^. Similarly, islands that were seemingly pushed towards their natural resource limits by pre-Iberian populations (including the small Micronesian islands and Hispaniola) are, from a biogeographic perspective, more sensitive to disturbance^61^ and appear to show a higher prevalence of forest regrowth after European contact. Interestingly, the more open habitat types that appear to show the greatest forest dynamism in response to Iberian conquest have, in general, lower carbon sequestration capacity than the apparently less sensitive, dense, perpetually humid rainforests of South America or Southeast Asia^6^—a factor that is overlooked in the calculation of the impact of early colonial era afforestation on the global carbon budget^21^. It is also worth noting that our dataset lacks coverage within key pre-Iberian and Iberian era urban hubs—regions that would be expected to show higher levels of ecological restructuring following colonization. Key under-researched sites include the Valley of Mexico (controlled by the populous Empire of the Triple Alliance at the time of Spanish contact) and important Spanish settlements in the Philippines (for example, Manila, which was central to European colonialism-driven biological exchange because it hosted the Philippines–Acapulco Galleon Trade). A lack of research within important Iberian hubs relates, at least in part, to the fact that most pollen-based studies focus on reconstructing past ecological–climate or human–environment relationships over much longer time scales, biasing site selection away from landscapes that have been heavily modified over the past ~1,000 years^40^. Targeted site selection to European settlement and trade centres, as well as improved chronological and sampling control within recent centuries, is thus an important element of future palaeoecological work. Indeed, these limitations have been raised in the context of recent work attempting to use palaeoecology to gauge pre-European landscape burning patterns in Northern America^62^.

Our dataset also documents, in many instances, afforestation in the centuries before Iberian conquest across the study area (that is, after 1000 ce). In several cases, this process actually appears to have exceeded early Iberian era forest regrowth in terms of scale. Notably, it suggests that coupled atmospheric–human drivers (Supplementary Text 1), social disruption and, potentially, ecosystem engineering by pre-colonial populations (Supplementary Text 3) may have been more important drivers of regional forest cover than Iberian contact. Particularly important climate drivers probably included increasing climatic variability between ~1050 and 1400 ce, linked to the El Niño–Southern Oscillation (ENSO)^63,64^, as well as the heterogeneous expression of the Medieval Warm Period (MWP) and LIA across the study region (see Figs. 3 and 5 for the approximate timing in the Spanish Americas and Spanish East Indies, respectively). For example, the MWP is linked to warmer, wetter conditions in the Northern Hemisphere in the centuries preceding Spanish arrival^65^ (Supplementary Text 1). As with the early Iberian era, increased forest regrowth before European contact also appears to have been partially controlled by biogeography. For instance, climatically driven increases in forest cover between 1200 and 1450 ce was more common in the seasonal ecosystems of western South America (including Los Llanos, Andean valley dry forests and the Brazilian Cerrado), as well as in the resource-limited Pacific islands, than in perpetually humid forests. However, it is also worth pointing out that climate-driven landscape changes were, in all likelihood, accompanied and potentially reinforced by human behavioural adaptations. For instance, there is evidence that Indigenous groups, including the Classic and post-Classic Maya and Inka, engaged in adaptive agroforestry and developed new agricultural practices to cope with climatic extremes^66^. Similarly, the ENSO-driven 1300 ce climate event in the Pacific, which resulted in a substantial depletion of available food resources (see Supplementary Text 3), is speculated to have led to the development of inland rather than coastal systems for procuring food, reef flat infill and construction of defensive infrastructure^67^. More detailed work is thus required to determine the changing intensity of pre-colonial and colonial human–environment–climate interactions in many of these tropical regions, such as has been conducted for the Brazilian highlands^68^.

Our data also demonstrate patterns of deforestation after Iberian arrival, both as a more immediate response to settlement and as a later response to the broader consequences of European colonialism, including the rise of capitalist European hegemony^69,70^ and the Great Acceleration^11^. Although currently limited in number, sites in Taiwan and the Philippines that are proximal to early Spanish centres hint at intensified land clearance following settlement (Fig. 4). A potential side effect of these settlements, which were usually established in agriculturally primed, governable lowlands may have been the active decision by Indigenous populations to migrate to less accessible uplands (for example, the Luzon highlands). It is plausible that this could have led to locally intensified land use and forest clearance within previously uncultivated areas as a corollary of a social and political resistance to colonial rule^58^. Sites in the Americas proximal to early Iberian settlements also indicate localized forest opening after an intensification of European land use. For instance, records from the Hispaniola lowlands show dramatic landscape opening in the sixteenth and seventeenth centuries following the establishment of intensive monocultural cropping systems^71^. Similarly, sites proximal to mining centres in the Andes and Veracruz, Mexico indicate intensified landscape disturbance following Iberian arrival^72^. Neotropical records from converted landscapes (for instance, those from the Atlantic Forests) highlight that industrial era deforestation far exceeds in magnitude any other shifts in forest cover over the past 2,000 years. The low temporal (subsampling) resolution of core tops and the spatial sampling bias towards sites surrounded by more intact ecosystems means that this change is likely to be under-represented in the assessed pollen records.

Overall, our analysis indicates that while forest regrowth did often occur following the decimation of large Indigenous populations after Iberian contact in the tropical Americas^21^, as well as the Asia-Pacific^29,73^, the timing and extent of observed afforestation in the early Iberian era appears contingent on spatially variable cultural and climatic factors coupled with ecoregion-specific resilience. Tropical forests reflect long-term land use legacies on an interhemispheric basis^15^. The murder, relocation and infection of Indigenous populations in many regions, as well as the floral and faunal exchanges that took place following Iberian colonialism, are essential considerations for re-evaluating the Anthropocene as a temporally variable and biogeographically/culturally contingent unequal process^13^. However, the variations in forest dynamics we observed before and after the initial period of Iberian contact and the establishment of colonies highlight the need to develop more detailed records of vegetation and land management change in different parts of the tropics, combining archaeology, palaeoecology and Indigenous traditional knowledge. This will permit a comprehensive exploration of the ways in which Indigenous resistance, invasive species, economic imbalances and the extension of colonial power and profit-driven land use left their varied marks on contemporary landscapes around the tropical world. Addressing these fine-scale, interdisciplinary questions will require well-dated, high-resolution palaeoenvironmental reconstructions spanning the past 2,000 years and covering the range of pre-colonial and colonial land use strategies that were present across the tropics. Only when such records become available can more realistic estimates of land use change and corresponding carbon fluxes be produced and fed into Earth systems models, with current projections^21^ likely to be simplifications. It is also clear that more refined understandings and records will enable conservation practitioners to grapple with the diverse socio-political, cultural and economic factors that have shaped, and continue to shape, the composition, diversity and resilience of tropical landscapes into the twenty-first century.

## Methods

Due to the inaccessibility of raw palynological data from sites in the Spanish East Indies relative to the Spanish Americas (shaded regions in Figs. 2a and 4a), palaeoecological data from each of these regions were extracted and prepared differently, as described below. For the Neotropics, we did not attempt to quantitatively reanalyse charcoal data from the region, as previous work has already demonstrated a sustained period of reduced biomass burning after ~500 cal yr bp^21,25,26^. In some instances, this change has been linked to a decrease in anthropogenic fire use following Iberian arrival and has been used to support the hypothesis that reduced land cultivation following a decrease in the population led to region-wide afforestation. However, site-specific discussion of the role of changing fire regimens relative to vegetation response are discussed in Supplementary Text 3 and included in our analyses where relevant. A composite curve of transformed charcoal influx (biomass burning) for the Americas (including North and South America)^26^, which shows a decrease in charcoal influx (biomass burning) between 1500 and 1650 ce to a minimum at 1650–1700 ce, is included in Fig. 3.

### Neotropics data preparation

We extracted Neotropical pollen datasets from the Neotoma Paleoecology Database^34^ (Neotoma), which were relevant for reconstructing tropical floristic change in the former Spanish Americas before, during and after Spanish colonization, using the following criteria:

1. The record was located within geopolitical units that were part of the former Spanish Empire (including Brazil).
2. The record was directly dated.
3. The record encompassed the time period spanning at least 600–1900 ce, permitting reasonable assessment of the scale of Iberian-induced change relative to the past 2,000 years. These datasets (*n* = 98; Supplementary Text 3; Supplementary Data 1) were individually assessed and selected for further analysis if:
4. The record derived from a terrestrial site that currently occurs within a tropical or subtropical biome^36^. If in a montane grassland, savanna and shrubland biome, the site was proximal (<5 km) to a tropical or subtropical biome.
5. The record included one sample that was estimated to come from the time frame 1500–1600 ce, thereby permitting assessment of floristic response to any Iberian-induced land use change.
6. The temporal resolution of the upper 2,000 years of the record (or total core length where the base of the record was <2,000 cal yr bp) was <200 years per sample.

The cut-off in criterion (6) was set in an attempt to capture forest turnover while maintaining a reasonable distribution of records across the study area. The Cobweb Swamp (Sawgrass Core)^74^ record, which has a resolution of 212 years per sample (Supplementary Data 1), was retained for analysis as it is situated within the heart of urban development across the Mesoamerican lowlands during the Classic Maya period. We set 2,000 years as an appropriate time frame for assessing ecological dynamics as it is short enough to identify late Holocene-scale floristic change while being long enough to assess the magnitude of Iberian-influenced change against the backdrop of pre-European land use dynamics and key late Holocene climate forcing (notably, the MWP and LIA and intensification of the ENSO^75,76^). An overview of how these events impacted the various geographic regions assessed in this study is outlined in Supplementary Text 1.

The application of the above criteria resulted in a final selection of 28 pollen records (from the same number of sites) for analysis. The setting, location and publication(s) associated with the selected records are detailed in Supplementary Data 1 and discussed in Supplementary Text 3.

The chronology and sampling resolution of the pollen records can influence whether rapid response dynamics are captured and appropriately constrained^77^. While this is problematic for many Neotropical records^78^, our selection of the pollen records based on sampling density and chronological control over the time period of interest attempts to selectively remove data that do not adequately capture ecosystem dynamics before, during and after the Iberian colonial period. We used the most up-to-date chronological models developed for the records in Neotoma and include a discussion of the interpreted ecological change, in the context of each chronological model produced for the records, as part of Supplementary Text 3.

A major obstacle to comparing palynological records across space (including different cultural zones and ecoregions) is the variability in the taxonomic resolution and the range of methods used by the original authors to classify vegetation change through time. To manage this, we adopted a two-pronged approach to assessing ecological change. First, we used the raw pollen counts from each record and consistently reclassified all individual pollen taxa into nine plant functional groups that can provide information about major state shifts in site vegetation through time. These functional groupings are listed in Extended Data Fig. 1. Classification was based on previously published work assigning different pollen types to biomes using surface pollen data from Latin America^79^. Once regrouped, we converted raw values to relative abundances and plotted each dataset stratigraphically against the age–depth models produced for each record (Supplementary Text 3). Major changes in the records were identified by clustering the data (method = coniss; distance = Euclidean; stratigraphically constrained)^80,81^. Second, the reclassified data for each site were used to calculate the ratio of non-arboreal to arboreal taxa as a proxy for landscape openness in the tropics^82,83^. This methodology attempts to eliminate interpretations of change based on fluctuations in aquatic, wetland and fern taxa, which are commonly driven by site-specific, local-scale hydrological shifts. This method assumes that grass pollen derives from a dryland versus wetland source. Changes in this ratio were cross-checked against shifts in the plant functional groupings, as well as the nature of the site type (Supplementary Text 3).

### Spanish East Indies data preparation

Neotoma and the Global Paleofire Database^84^ were searched for pollen and charcoal data within the time frame 0–2000 ce from countries within the former Spanish East Indies. This search returned four charcoal records (Supplementary Data 2) and no pollen records. We obtained an additional, unpublished raw charcoal dataset prepared by J.S. from a site (Lake Bulalacao) in the Philippines, and the raw pollen data were from Lake Paoay^48^. The five raw charcoal datasets were analysed by converting raw concentration values into influx rates (fragments per cm^2^ per year). The Lake Paoay pollen data were prepared by calculating and plotting the ratio of grass to arboreal pollen as a proxy for forest openness. This proxy was chosen to maintain consistency with other available pollen curves from the region.

To increase data capture within the Spanish East Indies, a review of regional published pollen and publications was conducted. Data from any plots of grass or dry herbs data plotted against depth (in most cases, used as a proxy for landscape openness), as well as any complimentary charcoal data, were extracted using WebPlotDigitizer^85^. While all efforts were made to ensure precise data extraction, minor sample or variable offsets may have been introduced as errors into the dataset depending on the quality of the initial graph production. Because some of these plots were made against depth (versus age), and the interpretation of age was based on outdated chronologies, an updated age–depth model for several of the cores was constructed using the program Bacon^86^ (R version 3.6.2)^81^ (details included in Supplementary Text 3). Because of the paucity of datasets from this part of the world, more liberal inclusion criteria were set for these datasets. Records were included if they captured environmental change within the 200-year period before Iberian contact (or a known disease-influenced population decrease) and at least one post-European sample. In the absence of pollen data, a single phytolith record was used to obtain a forest response signal from the island of Pohnpei. This was the only phytolith record used in the analysis given that phytoliths appear to be less sensitive to changes in tree cover than pollen in evergreen forests^87^, including the majority of the sites considered in this paper, and tend to represent more local rather than regional proxies for vegetation change, making them less useful than pollen for gauging broader afforestation signals.

The extracted data totalled ten pollen records, eight charcoal records and one phytolith record from 13 sites. Site details are outlined in the Supplementary Data 2 and discussed in Supplementary Text 3.

### Generalized additive modelling of palaeoecological data

The non-arboreal to arboreal ratios calculated for each Neotropical record, and the charcoal data and various pollen proxies for forest openness in the Spanish East Indies, were summarized using GAMs. This permitted an assessment of nonlinear trends in palaeoecological data, particularly those that are irregularly spaced in time, as is the case both within and between some of the Neotropical pollen datasets^88^. Data were first standardized by transformation (logit transformation for percentage data; log transformation for count and frequency data) and then standardized into *z* scores. Each GAM was fitted using thin-plate regression splines as the basis function^89^: the rank of the basis function was set to one-tenth the sample size or 5, whichever value was larger (ranks ranged from 5–26). GAM plots included 95% uncertainty intervals around the GAM fit line. Implementation of the GAM fits, calculation of uncertainty intervals and creation of record-specific GAM plots was undertaken in R version 3.6.2 (ref. ^81^) using the mgcv package (version 1.8-31)^90^. For data importation and manipulation, we used the packages data.table (version 1.12.8)^91^ and readxl (version 1.3.1)^92^. Resultant data are plotted stratigraphically in Fig. 3 (Spanish Americas) and Fig. 5 (Spanish East Indies). The analytical script used to create core-specific plots is available at the Open Science Framework (OSF) project page https://osf.io/gu483/, which also includes code to apply the same methods used in this paper to other datasets.

### Analysis of afforestation signal over 2,000 years

The palaeoecological proxies for forest cover, their associated GAMs, the known timings of Iberian contact and disease-induced population decrease (outlined for each region in Supplementary Text 2) and, for the Neotropical sites, cluster analysis of the plant function group (PFG) data, were used to semi-quantitatively assess whether the records showed afforestation or deforestation following Iberian contact, pre-Iberian (1000–1500 ce) afforestation or limited forest change over the past 1,000 years. We set 1,000 years as the time frame for classifying change as it is sufficiently long to provide a context for the pre-Iberian forest conditions while reducing the need to consider the influence of protracted mid- to late Holocene climate change on forest cover^40^. However, attention was given to the timing and asynchronous influence of shorter-term climate events on forest cover over the past 1,000 years (that is, the MWP and LIA), the regional influence of which is discussed in Supplementary Texts 1 and 3. The following criteria were used to classify the afforestation signal for each record:

- Post-Iberian afforestation (that is, afforestation after Iberian contact): the forest cover proxy data and GAM curvature show an increase in forest pollen in the 100-year time frame following a known population decrease associated with Iberian contact. For the Neotropics only, the clustered PFG data show that this shift is associated with a clear change in forest type and forest cover.
- Minor (or unclear) post-Iberian afforestation: either (1) the forest cover proxy data and GAM curvature show an increase in forest pollen in the 100-year time frame following a known population decrease associated with Iberian contact but the PFG data indicate forest stability over the same time period or (2) the forest cover proxy and PFG data indicate an increase in forest pollen in the 100-year time frame following a known population decrease associated with Iberian contact but this is not captured in the curvature of the GAM.
- Post-Iberian deforestation (that is, deforestation after Iberian contact): as for post-Iberian afforestation, but the data indicate forest opening rather than closing.
- Pre-Iberian afforestation: there is an afforestation or minor afforestation signal (as above) in the period between 1000 and 1400 ce for the Neotropical sites or between 1000 ce and the timing of Iberian contact for the Asia-Pacific sites.
- A limited forest response was determined if the records did not meet any of the above-listed criteria.

Changes in the Asia-Pacific charcoal records were assessed over the same time frames as those used for the above-discussed vegetation data. Decreases or increases in fire activity (as interpreted from the charcoal proxy data) after Iberian contact that were not captured in the GAM curvature were classified as minor/uncertain.

Site-by-site analysis of the palaeoecological and chronological trends and confidence for each record, together with other external supporting data, are presented in Supplementary Text 3.

The results of pre- and post-Iberian land use and forest change analysis were mapped for each region in ArcGIS Pro 2.5 (Fig. 2 and Fig. 4) and interpreted within both broad, pre-Iberian cultural groupings (Figs. 2a and 4a), as well within biomes (Figs. 2b and 4b).

### Reporting Summary

Further information on research design is available in the Nature Research Reporting Summary linked to this article.

## Supplementary information

Supplementary Information Supplementary Text 1–3, Figs. 1–34, Tables 1–3 and References.

Reporting Summary

Supplementary Data Details of the individual records used in this study. Supplementary Data 1: list of the Neotropical (Spanish American) records used in this study, including Neotoma codes, source, location and site details. Greyed-out records include the studies that did not meet the criteria for inclusion in our analyses (see column k and Methods for rationale). Supplementary Data 2: list of the Asia-Pacific (Spanish East Indies) records used in this study, including location, site and publication details.

## Data Availability

The synthesized datasets used to undertake the analyses are available at the following OSF project page: 10.17605/OSF.IO/GU483.

## Code availability

The analytical script used to create core-specific plots is available at the following OSF project page: 10.17605/OSF.IO/GU483. This includes code to apply the same methods used in this paper to other datasets.

## References

[1] Zalasiewicz J, Williams M, Haywood A, Ellis M (2011). The Anthropocene: a new epoch of geological time?. Phil. Trans. A Math. Phys. Eng. Sci..

[2] Ellis E, Maslin MA, Boivin N, Bauer A (2016). Involve social scientists in defining the Anthropocene. Nature.

[3] Ruddiman WF (2003). The Anthropogenic greenhouse era began thousands of years ago. Clim. Change.

[4] Crutzen PJ (2002). Geology of mankind. Nature.

[5] Gallery, R. E. in *Ecology and the Environment* (ed. Monson, R. K.) 247–272 (Springer, 2014).

[6] Pan Y (2011). A large and persistent carbon sink in the world’s forests. Science.

[7] Dinerstein E (2017). An ecoregion-based approach to protecting half the terrestrial realm. BioScience.

[8] Malhi Y, Gardner TA, Goldsmith GR, Silman MR, Zelazowski P (2014). Tropical forests in the Anthropocene. Annu. Rev. Environ. Resour..

[9] Staal A (2020). Hysteresis of tropical forests in the 21st century. Nat. Commun..

[10] Lenton TM (2019). Climate tipping points—too risky to bet against. Nature.

[11] Zalasiewicz J (2017). The Working Group on the Anthropocene: summary of evidence and interim recommendations. Anthropocene.

[12] Syvitski, J. et al. Extraordinary human energy consumption and resultant geological impacts beginning around 1950 CE initiated the proposed Anthropocene Epoch. *Commun. Earth Environ.*10.1038/s43247-020-00029-y (2020).

[13] Ruddiman, W. F. Three flaws in defining a formal ‘Anthropocene’. *Prog. Phys. Geogr. Earth Environ.*10.1177/0309133318783142 (2018).

[14] Smith BD, Zeder MA (2013). The onset of the Anthropocene. Anthropocene.

[15] Roberts P, Boivin N, Kaplan JO (2018). Finding the Anthropocene in tropical forests. Anthropocene.

[16] Boivin NL (2016). Ecological consequences of human niche construction: examining long-term anthropogenic shaping of global species distributions. Proc. Natl Acad. Sci. USA.

[17] Stephens L (2019). Archaeological assessment reveals Earth’s early transformation through land use. Science.

[18] Crosby, A. W. *The Columbian Exchange: Biological and Cultural Consequences of 1492* (Greenwood Publishing Group, 1972).

[19] Lewis SL, Maslin MA (2015). Defining the anthropocene. Nature.

[20] Denevan WM (1976). Estimating the Aboriginal population of Latin America in 1492: methodological synthesis. Publ. Ser. Conf. Lat. Am. Geogr..

[21] Koch A, Brierley C, Maslin MM, Lewis SL (2019). Earth system impacts of the European arrival and Great Dying in the Americas after 1492. Quat. Sci. Rev..

[22] Denevan, W. M. *The Native Population of the Americas in 1492* (Univ. Wisconsin Press, 1992).

[23] Denevan WM (2016). After 1492: nature rebounds. Geogr. Rev..

[24] Iriarte J (2012). Fire-free land use in pre-1492 Amazonian savannas. Proc. Natl Acad. Sci. USA.

[25] Nevle RJ, Bird DK, Ruddiman WF, Dull RA (2011). Neotropical human–landscape interactions, fire, and atmospheric CO_2_ during European conquest. Holocene.

[26] Power MJ (2013). Climatic control of the biomass-burning decline in the Americas after ad 1500. Holocene.

[27] Mann, C. C. *Uncovering the New World Columbus Created* (Knopf Publishing Group, 2011).

[28] Hung H-C (2007). Ancient jades map 3,000 years of prehistoric exchange in Southeast Asia. Proc. Natl Acad. Sci. USA.

[29] Newson, L. A. *Conquest and Pestilence in the Early Spanish Philippines* (Univ. Hawaii Press, 2009).

[30] Amano N, Bankoff G, Findley DM, Barretto-Tesoro G, Roberts P (2021). Archaeological and historical insights into the ecological impacts of pre-colonial and colonial introductions into the Philippine Archipelago. Holocene.

[31] Acabado, S. B. in *Irrigated Taro (Colocasia esculenta) in the Indo-Pacific (Senri Ethnological Studies 78)* (eds Spriggs, M. et al.) 285–305 (National Museum of Ethnology, 2012).

[32] Junker, L. L. *Raiding, Trading, and Feasting: The Political Economy of Philippine Chiefdoms* (Ateneo de Manila Univ. Press, 2000).

[33] Amano N, Piper PJ, Hung H-C, Bellwood P (2013). Introduced domestic animals in the Neolithic and Metal Age of the Philippines: evidence from Nagsabaran, Northern Luzon. J. Isl. Coast. Archaeol..

[34] Williams JW (2018). The Neotoma Paleoecology Database, a multiproxy, international, community-curated data resource. Quat. Res..

[35] Boivin N, Crowther A (2021). Mobilizing the past to shape a better Anthropocene. Nat. Ecol. Evol..

[36] Olson DM (2001). Terrestrial ecoregions of the world: a new map of life on Earth: a new global map of terrestrial ecoregions provides an innovative tool for conserving biodiversity. BioScience.

[37] Berrio JC, Hooghiemstra H, Behling H, Botero P, Van der Borg K (2002). Late-Quaternary savanna history of the Colombian Llanos Orientales from Lagunas Chenevo and Mozambique: a transect synthesis. Holocene.

[38] Berrío JC, Hooghiemstra H, Marchant R, Rangel O (2002). Late-glacial and Holocene history of the dry forest area in the south Colombian Cauca Valley. J. Quat. Sci..

[39] Mayle FE, Burbridge R, Killeen TJ (2000). Millennial-scale dynamics of southern Amazonian rain forests. Science.

[40] Flantua SGA (2016). Climate variability and human impact in South America during the last 2000 years: synthesis and perspectives from pollen records. Climate.

[41] Polissar PJ (2006). Solar modulation of Little Ice Age climate in the tropical Andes. Proc. Natl Acad. Sci. USA.

[42] Maezumi SY (2018). The legacy of 4,500 years of polyculture agroforestry in the eastern Amazon. Nat. Plants.

[43] Leyden, B. W. et al. in *The Managed Mosaic: Ancient Maya Agriculture and Resource Use* (ed. Fedick, S. L.) (Univ. Utah Press, 1996).

[44] Dodson JR, Intoh M (1999). Prehistory and palaeoecology of Yap, Federated States of Micronesia. Quat. Int..

[45] Athens JS, Ward JV (2004). Holocene vegetation, savanna origins and human settlement of Guam. Rec. Aust. Mus. Suppl..

[46] Levin MJ, Ayres WS (2017). Managed agroforests, swiddening, and the introduction of pigs in Pohnpei, Micronesia: phytolith evidence from an anthropogenic landscape. Quat. Int..

[47] Chen S-H (2009). Late Holocene paleoenvironmental changes in subtropical Taiwan inferred from pollen and diatoms in lake sediments. J. Paleolimnol..

[48] Stevenson J, Siringan F, Finn JAN, Madulid D, Heijnis H (2010). Paoay Lake, northern Luzon, the Philippines: a record of Holocene environmental change. Glob. Change Biol..

[49] Wigboldus JS (1987). A History of the Minahasa c. 1615–1680. Archipel.

[50] United States Office of the Chief of Naval Operations. *West Caroline Islands* (Office of the Chief of Naval Operations, Navy Department, 1943).

[51] De Souza JG (2019). Climate change and cultural resilience in late pre-Columbian Amazonia. Nat. Ecol. Evol..

[52] Leal, C. *Landscapes of Freedom: Building a Postemancipation Society in the Rainforests of Western Colombia* (Univ. Arizona Press, 2018).

[53] Block, D. *Mission Culture on the Upper Amazon: Native Tradition, Jesuit Enterprise & Secular Policy in Moxos, 1660*–*1880* (Univ. Nebraska Press, 1994).

[54] Callaghan R, Fitzpatrick SM (2007). On the relative isolation of a Micronesian archipelago during the historic period: the Palau case-study. Int. J. Nautical Archaeol..

[55] Da Silva CM (2019). The miracle of the Brazilian Cerrados as a juggernaut: soil, science, and national culture. Hispanic Issues Ser..

[56] Goldberg, W. M. *The Geography, Nature and History of the Tropical Pacific and Its Islands* (Springer, 2017).

[57] Schwaller RC (2018). Contested conquests: African maroons and the incomplete conquest of Hispaniola, 1519–1620. Americas.

[58] Acabado SB (2019). The short history of the Ifugao rice terraces: a local response to the Spanish conquest. J. Field Archaeol..

[59] Iriarte J (2020). The origins of Amazonian landscapes: plant cultivation, domestication and the spread of food production in tropical South America. Quat. Sci. Rev..

[60] Staver AC, Archibald S, Levin SA (2011). The global extent and determinants of savanna and forest as alternative biome states. Science.

[61] Graham NR, Gruner DS, Lim JY, Gillespie RG (2017). Island ecology and evolution: challenges in the Anthropocene. Environ. Conserv..

[62] Roos CI (2020). Scale in the study of Indigenous burning. Nat. Sustain..

[63] Lu, Z., Liu, Z., Zhu, J. & Cobb, K. M. A review of paleo El Niño-Southern Oscillation. *Atmosphere*10.3390/atmos9040130 (2018).

[64] Shi F, Li J, Wilson RJ (2014). A tree-ring reconstruction of the South Asian summer monsoon index over the past millennium. Sci. Rep..

[65] Newton, A., Thunell, R. & Stott, L. Climate and hydrographic variability in the Indo-Pacific Warm Pool during the last millennium. *Geophys. Res. Lett.*10.1029/2006gl027234 (2006).

[66] Chepstow-Lusty A, Winfield M (2000). Inca agroforestry: lessons from the past. Ambio.

[67] Nunn P (2000). Environmental catastrophe in the Pacific Islands around A.D. 1300. Geoarchaeology.

[68] Robinson M (2018). Uncoupling human and climate drivers of late Holocene vegetation change in southern Brazil. Sci. Rep..

[69] Green WA (1986). The New World and the rise of European capitalist hegemony: some historiographical perspectives. Itinerario.

[70] Wolf, E. R. *Europe and the People Without History* (Univ. California Press, 2010).

[71] Castilla-Beltrán A (2018). Columbus’ footprint in Hispaniola: a paleoenvironmental record of indigenous and colonial impacts on the landscape of the central Cibao Valley, northern Dominican Republic. Anthropocene.

[72] Goman M, Byrne R (1998). A 5000-year record of agriculture and tropical forest clearance in the Tuxtlas, Veracruz, Mexico. Holocene.

[73] Francis X, Hezel SJ (2010). Disease in Micronesia: a historical survey. Pac. Health Dialogue.

[74] Jones, J. G. *Pollen Evidence of Prehistoric Forest Modification and Maya Cultivation in Belize*. PhD thesis, Texas A&M Univ. (1991).

[75] Rojas M, Arias PA, Flores-Aqueveque V, Seth A, Vuille M (2016). The South American monsoon variability over the last millennium in climate models. Climate.

[76] Rosenthal Y, Linsley BK, Oppo DW (2013). Pacific ocean heat content during the past 10,000 years. Science.

[77] Blois JL, Williams JW, Grimm EC, Jackson ST, Graham RW (2011). A methodological framework for assessing and reducing temporal uncertainty in paleovegetation mapping from late-Quaternary pollen records. Quat. Sci. Rev..

[78] Flantua SGA, Blaauw M, Hooghiemstra H (2016). Geochronological database and classification system for age uncertainties in Neotropical pollen records. Climate.

[79] Marchant R (2009). Pollen-based biome reconstructions for Latin America at 0, 6000 and 18 000 radiocarbon years ago. Climate.

[80] Juggins, S. rioja: Analysis of Quaternary Science Data. R package version 0.9-21. https://cran.r-project.org/package=rioja (2014)..

[81] R Core Development Team *R: A Language and Environment for Statistical Computing* (R Foundation for Statistical Computing, 2013).

[82] Hamilton, R., Penny, D. & Hall, T. L. Forest, fire & monsoon: investigating the long-term threshold dynamics of south-east Asia’s seasonally dry tropical forests. *Quat. Sci. Rev.*10.1016/j.quascirev.2020.106334 (2020).

[83] Bhagwat SA, Nogué S, Willis KJ (2012). Resilience of an ancient tropical forest landscape to 7500 years of environmental change. Biol. Conserv..

[84] Blarquez O (2014). paleofire: an R package to analyse sedimentary charcoal records from the Global Charcoal Database to reconstruct past biomass burning. Comput. Geosci..

[85] Rohatgi, A. WebPlotDigitizer v4.2 https://automeris.io/WebPlotDigitizer (2019).

[86] Blaauw M, Christen JA (2011). Flexible paleoclimate age–depth models using an autoregressive gamma process. Bayesian Anal..

[87] Plumpton H, Whitney B, Mayle F (2019). Ecosystem turnover in palaeoecological records: the sensitivity of pollen and phytolith proxies to detecting vegetation change in southwestern Amazonia. Holocene.

[88] Simpson, G. L. Modelling palaeoecological time series using generalised additive models. *Front. Ecol. Evol.*10.3389/fevo.2018.00149 (2018).

[89] Wood SN (2003). Thin plate regression splines. J. R. Stat. Soc. Ser. B Stat. Methodol..

[90] Wood SN (2011). Fast stable restricted maximum likelihood and marginal likelihood estimation of semiparametric generalized linear models. J. R. Stat. Soc. Ser. B Stat. Methodol..

[91] Dowle, M. et al. data.table: Extension of ‘data.frame’. R package version 0.9-21. https://cran.r-project.org/web/packages/data.table (2018).

[92] Wickham, H. & Bryan, J. readxl: Read Excel Files. R package version 1.3.1. https://CRAN.R-project.org/package=readxl (2019).

[93] Doeppers DF (1972). The development of Philippine cities before 1900. J. Asian Stud..

[94] Dobyns HF (1993). Disease transfer at contact. Annu. Rev. Anthropol..

[95] Watts WA, Bradbury JP (1982). Paleoecological studies at Lake Patzcuaro on the west-central Mexican Plateau and at Chalco in the Basin of Mexico. Quat. Res..

[96] Van Hengstum PJ (2016). The intertropical convergence zone modulates intense hurricane strikes on the western North Atlantic margin. Sci. Rep..

[97] Crausbay SD, Martin PH, Kelly EF, McGlone M (2015). Tropical montane vegetation dynamics near the upper cloud belt strongly associated with a shifting ITCZ and fire. J. Ecol..

[98] Kelly TJ (2017). The vegetation history of an Amazonian domed peatland. Palaeogeogr. Palaeoclimatol. Palaeoecol..

[99] Carson JF (2014). Environmental impact of geometric earthwork construction in pre-Columbian Amazonia. Proc. Natl Acad. Sci. USA.

[100] Berrio JC, Hooghiemstra H, Behling H, van der Borg K (2000). Late Holocene history of savanna gallery forest from Carimagua area, Colombia. Rev. Palaeobot. Palynol..

[101] Ledru M-P (1993). Late quaternary environmental and climatic changes in central Brazil. Quat. Res..

[102] Ledru M-P, Behling H, Fournier M, Martin L, Servant M (1994). Localisation de la forêt d'Araucaria du Brésil au cours de l'Holocène. Implications paléoclimatiques. C. R. Acad. Sci. Paris.

[103] Behling H (1995). A high resolution Holocene pollen record from Lago do Pires, SE Brazil: vegetation, climate and fire history. J. Paleolimnol..

[104] Behling H (1997). Late Quaternary vegetation, climate and fire history from the tropical mountain region of Morro de Itapeva, SE Brazil. Palaeogeogr. Palaeoclimatol. Palaeoecol..

[105] Behling H, Hooghiemstra H, Negret AJ (1998). Holocene history of the Chocó rain forest from Laguna Piusbi, southern Pacific lowlands of Colombia. Quat. Res..

[106] Vélez MI (2001). Late Holocene environmental history of southern Chocó region, Pacific Colombia; sediment, diatom and pollen analysis of core El Caimito. Palaeogeogr. Palaoclimatol. Palaeoecol..

[107] Vélez MI, Berrío JC, Hooghiemstra H, Metcalfe S, Marchant R (2005). Palaeoenvironmental changes during the last ca. 8590 calibrated yr (7800 radiocarbon yr) in the dry forest ecosystem of the Patía Valley, Southern Colombian Andes: a multiproxy approach. Palaeogeogr. Palaeoclimatol. Palaeoecol..

[108] Behling H, Negret AJ, Hooghiemstra H (1998). Late Quaternary vegetational and climatic change in the Popayán region, southern Colombian Andes. J. Quat. Sci..

[109] Wille M, Hooghiemstra H, Behling H, van der Borg K, Negret AJ (2001). Environmental change in the Colombian subandean forest belt from 8 pollen records: the last 50 kyr. Veg. Hist. Archaeobot..

[110] Epping, I. *Environmental Change in the Colombian Upper Forest Belt.* MSc thesis, Univ. Amsterdam (2009).

[111] Niemann H, Behling H (2009). Late Pleistocene and Holocene environmental change inferred from the Cocha Caranga sediment and soil records in the southeastern Ecuadorian Andes. Palaeogeogr. Palaeoclimatol. Palaeoecol..

[112] Graf, K. in *Pollendiagramme aus den Anden, eine Synthese zur Klimageschichte und Vegetationsentwicklung Seit der letzten Eiszeit*. Vol. 34 (Univ. Zurich, 1992).

[113] Kuhry, P., Salomons, J. B., Riezebos, P. A. & Van der Hammen, T. in *Studies on Tropical Andean Ecosystems/Estudios de Ecosistemas Tropandinos: La**Cordillera Central Colombiana Transecto Parque Los Nevados* (eds van der Hammen, T. et al.) 227–261 (Cramer, 1983).

[114] Velásquez-R CA, Hooghiemstra H (2013). Pollen-based 17-kyr forest dynamics and climate change from the Western Cordillera of Colombia; no-analogue associations and temporarily lost biomes. Rev. Palaeobot. Palynol..

[115] Rull V, Salgado-Labouriau M-L, Schubert C, Valastro S (1987). Late Holocene temperature depression in the Venezuelan Andes: palynological evidence. Palaeogeogr. Palaeoclimatol. Palaeoecol..

[116] Van der Hammen T (1962). Palinología de la región de “Laguna de los Bobos”: historia de su clima, vegetación y agricultura durante los últimos 5.000 años. Revista de la Academia Colombiana de Ciencias Exactas. Físicas Nat..

[117] Wang LC (2014). Late Holocene environmental reconstructions and their implications on flood events, typhoon, and agricultural activities in NE Taiwan. Climate.

[118] Dam RAC, Fluin J, Suparan P, van der Kaars S (2001). Palaeoenvironmental developments in the Lake Tondano area (N. Sulawesi, Indonesia) since 33,000 yr B.P. Palaeogeogr. Palaeoclimatol. Palaeoecol..

[119] Suparan P, Dam RAC, van der Kaars S, Wong TE (2001). Late Quaternary tropical lowland environments on Halmahera, Indonesia. Palaeogeogr. Palaeoclimatol. Palaeoecol..

[120] Athens, J. S. & Ward, J. V. *Holocene Paleoenvironmental Investigations on Ngerekebesang, Koror, South Babeldaob, and Peleliu Islands, Palau* (International Archaeological Research Institute, 2002).

[121] Athens, J. S. & Ward, J. V. *Palau Compact Road Archaeological Investigations, Babeldaob Island, Republic of Palau. Phase I: Intensive Archaeological Survey. Volume IV: Holocene Paleoenvironment and Landscape Change* (International Archaeological Research Institute, 2005).

