# Supplementary material for: Non-uniform tropical forest responses to the ‘Columbian Exchange’ in the Neotropics and Asia-Pacific
Source: Nat Ecol Evol. 2021 Jun 10;5(8):1174–84. doi: 10.1038/s41559-021-01474-4 (PMC8324576; doi:10.1038/s41559-021-01474-4)
Supplement: Supplementary file 1 — Supplementary Text 1–3, Figs. 1–34, Tables 1–3 and References. [file 41559_2021_1474_MOESM1_ESM.pdf]

nature portfolio

https://doi.org/10.1038/s41559-021-01474-4

Supplementary information

Non-uniform tropical forest responses to the 'Columbian Exchange' in the Neotropics and Asia-Pacific

In the format provided by the authors and unedited

Supplementary Information for 'Non-uniform tropical forest regrowth response to the 'Columbian Exchange' in the Neotropics and Asia-Pacific'

Rebecca Hamilton[1,2,3*], Jesse Wolfhagen[1], Noel Amano[1], Nicole Boivin[1,4,5,6], David Max Findley[1], José Iriarte[7], Jed O. Kaplan[8], Janelle Stevenson[2,3], Patrick Roberts[1,4,9*]

[1]Max Planck Institute for the Science of Human History, Jena, Germany

[2]College of Asia and the Pacific, Australian National University, Canberra, Australia

[3]Australian Research Council Centre of Excellence for Australian Biodiversity and Heritage, The Australian National University, Canberra, ACT, Australia

[4]School of Social Sciences, University of Queensland, St Lucia, Australia

[5]Department of Anthropology and Archaeology, University of Calgary, Calgary, Canada

[6]Department of Anthropology, National Museum of Natural History, Smithsonian Institution, Washington, DC, USA.

[7]Department of Archaeology, College of Humanities, University of Exeter, Exeter, UK

[8]Department of Earth Science, The University of Hong Kong, Hong Kong

[9]Department of Archaeology, University of the Philippines, Quezon City, The Philippines

*correspondence to: Rebecca Hamilton, Patrick Roberts

email: hamilton@shh.mpg.de, roberts@shh.mpg.de

This PDF file includes:

Supplementary Text 1:	Overview of the regional climate context for the Spanish Americas and Spanish East Indies over the past 2,000 years
Supplementary Text 2:	Review of pre-Iberian human land-use and impact of Spanish/ Portuguese contact on the population dynamics within geographical units defined for Spanish East Indies and Spanish American sites.
Supplementary Text 3:	Site-by-site analysis of ecosystem change from pollen records included in the study
Supplementary Figure 1 (Extended Data Figure):	Pollen percentage diagrams and cluster analysis of plant functional types from the Neotropical sites analysed in this study.
Supplementary Figures 2-34:	Chronological models for the records analysed in this study.
Supplementary Table 1:	Summary table of pre-Iberian land-use, the timing of Iberian contact and settlement, and Iberian-induced land-use- and population- change within the Spanish East Indies
Supplementary Table 2:	Summary table of pre-Iberian land-use, the timing of Iberian contact and settlement, and Iberian-induced land-use- and population- change within the Spanish Americas.
Supplement Table 3:	Sample ID, depth, and 14C age of samples extracted from 445 cm long sediment core extracted from Lake Bululacao, the Philippines.

References

Supplementary Text 1: Overview of the regional climate context for the Spanish Americas and Spanish East Indies over the past 2,000 years.

This section provides a brief and generalized overview of the palaeoclimate of the study regions over the past 2,000 years in order to provide a climatic context for vegetation and social changes within the former tropical Spanish Empire. These changes are discussed separately for tropical regions in the Asia-Pacific and the Americas that were incorporated into the Spanish Empire (termed the Spanish East Indies and the Spanish Americas, respectively).

SPANISH EAST INDIES

Time period (CE)	Climate driver	Influence on regional climate
>50 to 900	Precession-driven decrease in Northern Hemisphere summer insolation leads to southerly placement of Intertropical Convergence Zone (ITCZ) and weaker Southeast Asian Summer Monsoon[1]; Cooler sea surface temperatures (SST) in southern Indo-Pacific Warm Pool result in strengthened El Niño–Southern Oscillation (ENSO)[2,3]	*Northern Hemisphere:* Reduced precipitation<br>*Southern Hemisphere:* Variable, sometimes anti-phased conditions across space, including aridity in northwest Australia; muted impacts at equator[2,3]
~900 to 1200 (Medieval Warm Period [MWP])[4]	Northward migration of the ITCZ; warmer SSTs in Indo-Pacific Warm Pool	*Northern Hemisphere:* Strengthened Southeast Asian Summer Monsoon leads to warm, wet conditions[1,4,5].<br>*Southern Hemisphere:* Weakened ENSO results in wetter conditions[2]
1300 to 1550	Rapid SST cooling in the Pacific; strengthened ENSO, a weak Southeast Asian Summer Monsoon, falling sea levels[6-9]	Heightened climate variability[10] including drought events.<br>*Oceania & Taiwan:* Heightened tropical cyclone activity; sea level fluctuations; drought[11]
1550 to 1850 (Little Ice Age [LIA])[4]	Southern displacement of the ITCZ[9,12,13] or equatorial contraction of the ITCZ[14]; drop in Pacific SST by approximately 0.75°C[4]; further ENSO strengthening[6]; movement of ITCZ north from ~1650 CE[9].	More arid conditions (more muted in southern and equatorial sites than those at higher latitudes)[12]<br>*Oceania & Taiwan:* early LIA (<1550 to 1620 CE) characterized by dry, stable climate; late LIA (1640 to 1850 CE) associated with an increase in precipitation and cyclones[10].
1850 to present	Human forced atmospheric warming; sea level rise; strong ENSO	20th and 21st century warming. Increased frequency and intensity of tropical cyclones; temperature rise.

		Northern hemisphere: Strengthening of the Southeast Asian summer monsoon[7] *Southern hemisphere:* Increased total precipitation over northern Australia[15].

SPANISH AMERICAS

A generalized summary of climate drivers and regional climate responses for the Spanish Americas (Fig. 1) is summarized below. Note that a detailed overview of the palaeoclimate of this region over the past 2,000 years, on which much of this section is based, is discussed in[16].

**Time period (CE)**	**Climate driver**	**Influence on regional climate**
50 BCE to ~500-750 CE	Southerly placement of Intertropical Convergence Zone (ITCZ).	Relatively stable; *Northern Hemisphere:* relatively dry [17,18] *Southern Hemisphere:* relatively humid[19,20]
~500-750 to 1050	Heightened ENSO[21]; fluctuations in Atlantic Meridional Overturning Circulation (AMOC) associated with a southwards shift in the ITCZ-and variability in sea surface salinity (SSS) and sea surface temperature (SST) gradients[22].	Increased variability (implicated in the "Terminal Classical Droughts" of the Maya region [22] & fall of the city of Teotihuacán[21]).
950 to 1250 (MWP)[23]	Northward migration of the ITCZ leads to warmer north tropical Atlantic SST, and cooler south equatorial Atlantic SST[23] and a weakened South American Summer Monsoon; In South America, the South Atlantic Convergence Zone (SACZ) causes variable climate between the east and west Amazon basin[24]; ENSO & Interdecadal Pacific Oscillation (IPO) are key modulating factors[16].	Overall heightened variability, but differential impacts across space (latitudinally and longitudinally).<br>Generalised patterns are:<br>*Northern Hemisphere:* wetter conditions[23]<br>*west Southern Hemisphere:* drier conditions[25]<br>*central & east Southern Hemisphere:* early MWP (900 to 1150 CE) = humid conditions; Late MWP 1150 and 1350-1400 CE = arid conditions[26,27]
1250 to 1450	Southwards migration of the ITCZ	LIA to MWP transitional conditions
1450 to 1850 (LIA)[23]	Southerly placement of the ITCZ leads to cooler tropical north Atlantic SST and warmer tropical south Atlantic SST[23]; In South America, the South Atlantic Convergence Zone (SACZ) causes variable climate between the east and west Amazon basin[24]; El Niño	Differential impacts across space (latitudinally and longitudinally).<br>Generalised patterns are:

	warming in the Pacific (ENSO important in east South America)[23,25]	*Northern Hemisphere:* drier conditions[28]<br>*west Southern Hemisphere:* cooler, wetter conditions[29,30]<br>*central & east Southern Hemisphere: variable as climate* strongly modulated by ENSO.
1850 to present	ENSO becomes the dominant control on rainfall[31]. human forced atmospheric warming; sea level rise; strong ENSO	20th century warming; heightened variability in precipitation both across space and through time[31]

Supplementary Text 2: Review of pre-Iberian human land-use and impact of Spanish/ Portuguese contact on the population dynamics and economies within geographical units defined for Spanish East Indies and Spanish American sites

The presence of a sufficiently large Indigenous population, a notable impact of these people on the environment, and a population decline subsequent to Iberian impact are considered necessary pre-requisites for an anthropogenic afforestation response to the 'Columbian Exchange'[32]. These dynamics are considered below, in the context of the Spanish East Indies, and, more briefly, in the context of the Spanish Americas given the recent publication of a review paper on this topic[32]. We place an increased focus on regions that were analysed in this study.

SPANISH EAST INDIES

Human-landscape dynamics within the Spanish East Indies are considered in the context of four geographically defined zones: 1) North Taiwan (Spanish Formosa); 2) The Philippines; 3) Wallacea (north Sulawesi [Minahasa] and north Maluku), and; 4) Micronesia. By the time of Iberian arrival, it is generally accepted that all of these regions had been reached by Austronesian-speaking farming communities who had introduced novel cultivation practices as well as the domesticated pig, chicken, and dog[33,34]. One of the key cultivation practices associated with the Austronesian language groups was the production of rice (*Oryza sativa*) – the earliest regional evidence of which comes from eastern Taiwan, dated to 4,200 yrs BP[35]. While arrival of Austronesian-speakers to Taiwan, island Southeast Asia and the Pacific is suggested to have regionally intensified land-use, increased population sizes, and reduced forest cover, particularly in the uplands[36], signals of dramatic land-use overhaul remains to be seen in the archaeological and palaeoecological record[37]. Additionally, there is evidence for the cultivation of starchy root-crops in the region long prior to Austronesian arrival, and a growing body of research demonstrates the ongoing use and management of wild tropical forest resources by both Austronesian-speaking communities and hunter-gatherers[38-41]. Below, we evaluate the existing evidence for pre-European land-use in these geographic regions at- and prior to- Spanish/Portuguese arrival, their likely impact on environments, and the political, social, economic, and environmental consequences of Iberian colonization.

North Taiwan

Proto-Austronesian language groups settled in Taiwan from 5,500 yrs BP[42]. Neolithic archaeology from the island indicates that food production at this time reflects adaptation to environmental heterogeneity, and included the cultivation of tubers, cereals (especially rice and foxtail millet (*Setaria italica*)[43,44]), as well as foraging and fishing[44]. In the millennia following 5,500 yr BP, Taiwan geographically split into different cultural groups. In northern Taiwan – the region later incorporated into the Spanish East Indies – the Xuntangpu-Yuanshan-Zhiwuyuan cultures emerged in the Neolithic followed by the Shihsanhang culture

of the Iron Age [45,46]. At the time of Spanish contact in the 1600s, Indigenous food production was focused on fishing and hunting (particularly for deer) as well as cultivation of rice, tubers and fruits, resources that were commonly traded between different groups[43,47]. The impact of humans on the landscape was thus heterogenous and contingent on factors such as soil fertility and proximity to the coast[43].

The first Europeans to set foot on the island were Portuguese sailors who were shipwrecked for 45 days in 1582[48]. By the 1600s, north Taiwan had captured the eyes of the Spanish, who were interested in exploiting the natural resources of the island, setting up defensive bases against the Dutch, and establishing a strategic port for trading with China[43]. Spanish occupation was short, spanning 1626 to 1641 CE, and concentrated around bases at Keelung and Fort San Domingo (Fig. 1) before the region was taken over by the Dutch. Pulses of endemic disease, typically associated with the start of Dutch rule in 1635 CE, are expected to have had a significant impact on the mortality of Indigenous people. The population of Taiwan between 1640 and 1650 CE was estimated at 100,000[49] – a number already thought to be significantly lower than the pre-European population size, though the exact scale of change remains debated[41]. By 1654, the total Indigenous population was estimated at approximately 50,000 people[43].

The Philippines

A comprehensive overview of the archaeology of the Philippines in the context of human-landscape relationships is the subject of a recent review paper[50]. The main points from this review are summarized here. Speakers of the Proto-Malayo-Polynesian branch of the Austronesian language group are thought to have moved out of Taiwan to Luzon between 2500 and 1500 BCE[51]. This first phase of settlement is associated with the introduction of: 1) a novel set of agricultural crops (allegedly based around rice cultivation, though evidence for this is scarce[52]); 2) a distinct "cultural package" (red-clipped pottery, spindle whorls, bark-cloth beater, and polished stone tools), and; 3) domesticated animals (pigs, dogs, and possibly chickens)[42,53].

Evidence for Neolithic agriculture in the Philippines is rare, potentially due to poor preservation and lack of archaeological records[50]. Hunter-gatherers may have been processing taro (*Colocasia esculenta*) prior to the arrival of farmers speaking Austronesian languages, though there is limited archaeological evidence of this in the Philippines[54], with most work focused on identifying taro and yam (*Dioscorea*) tissues based in nearby Borneo. Domesticated animals (including the pig) were present in the Neolithic landscape[55], however, they do not appear to have been used as widespread dietary staples[50]. Very little archaeobotanical research has been done on Neolithic and Metal Period sites, but historical documents and landscape features (i.e. terraces) suggest increased cultivation of rice in some parts of the archipelago through time, possibly driven by population growth[50].

Early Spanish accounts of land-use indicate that, at the time of colonization, rice, taro, banana (*Musa*), coconut (*Cocos nucifera)*, tropical forest fruits, and sugarcane (*Saccharum*) were well assimilated into mixed subsistence economies[56]. This may have involved broad-scale landscape modification including swiddening in the lowlands (for dryland rice) and terracing and irrigation (for wet rice and root crops)[50,56]. As with Taiwan, the impact of people on the landscape appears to be geographically contingent. For instance, it has been postulated that land-use within the rugged uplands of Luzon was more fluid, with people relying on shifting agriculture, hunting and foraging[57] – a practice that in some places has persisted until recent times[58]. Nevertheless, by the time of European arrival there is evidence for the emergence of significant coastal polities, for example at Manila and Cebu[59], as well as increasing use and clearance of the land for cultivation of wet and dry rice[60].

The impact of disease and conflict associated with Spanish contact (1521 CE), is generally thought to have been lower in the Philippines than elsewhere in the Spanish Empire. This is a result of the supposedly low pre-Spanish population densities, the physical geography of the archipelago, existing immunity from relationships with so-called "Old World" domesticates (the Philippines had frequent trade contact with China from 400 CE), and the resistance of Indigenous communities to Spanish attempts to agglomerate populations[61,62]. Nonetheless, population decline after Spanish occupation was still significant. The population of Luzon, for instance, dropped~40% from an estimated 1.05 million to 638,760 between 1565 and 1591 CE[62,63]. This tracks the broader trend of population decline being highest for islands that experienced sustained contact with the Spanish (e.g. Luzon and Visayas)[63]. In Luzon, the cities of Ilocos, Cagayan, Bikol, and Pampanga lost >40% of their pre-Spanish population, while in Manila, high rates of mortality were mitigated by immigration[63]. It is key to note that, due to poor record keeping and translocation of populations to the Luzon lowlands under the Spanish Policy of *reducción* (consolidation and resettlement of the population to enable mass conversion to Catholicism), Indigenous populations were likely underestimated. A second wave of population decline (~46%) occurred in Luzon in the early 17th century[63].

In addition to population decline, Spanish occupation altered the settlement patterns, administration, and economies of the Philippines by introducing land tenure systems, plantation agriculture, ranching, and the extraction of resources for shipbuilding, fortification, and urbanisation[56,64]. Not only that, but a significant amount of archaeological and historical research has demonstrated that Indigenous societies, particularly in the Ifugao highlands of Luzon, through their staunch resistance to Spanish rule, altered their political, social, and economic organization in the absence of direct Spanish control[65,66]. Indeed, the overall impacts of Spanish urbanism, economic structures, and land tenure are generally thought to have been much lower, and less effective, when compared to Iberian rule in many parts of the Americas[50,67,68].

Wallacea

North Sulawesi

North Sulawesi was settled by Austronesian speakers in a series of waves[69] between 1550 and 1000 BCE[42]. This resulted in the establishment of disparate communities within the region. Oral histories indicate land clearance and shifting dryland cultivation across the pre-Iberian landscape[70]. Wet rice was farmed in the vicinity of lake basins (including at Lake Tondano – a site analyzed in this study) since at least the 16th century[71]. Pork and rice appear to have been dietary staples at the time of Iberian contact[69]. Local oral histories do, however, extend back to a time when rice was not cultivated and "fruit from a vine" was the main food source[69].

Northeast Sulawesi, being proximal to the Spice Islands, was incorporated into the Sultanate of Ternate at the time of Portuguese (1530 to 1540 CE) and Spanish (1549 CE) arrival in Minahasa[69]. Efforts by the Iberians to infiltrate the region were initially hindered by the Sultanate of Ternate, and later impacted by significant conflict in the region between 1572 and 1617 CE[69]. Nonetheless, evidence of maize grown alongside swidden crops in Minahasa from the 1580s provides an early indication of the "Columbian Exchange" in Sulawesi[69]. A shift from mission-focused maritime imperialism to colonization – which had a political and economic (including rice export) focus – occurred between 1606 and 1616 CE[69]. Conflict between Spanish, Minahasan, and later Dutch groups persisted between 1642 and 1657 CE, terminating with the expulsion of the Spanish from the island by the Dutch[71].

There are large discrepancies in estimations made of Minahasa's 17th century population. However a conservative estimate of 50,000 has been made for the region at both the start and end of the century[71]. There appears to be a clear decrease in this number between 1644 and 1679 CE due to disease and impacts of Europeans on local social and economic structures[71].

Maluku

Halmahera – the largest island in North Maluku – was occupied between 1500 and 1000 BCE by Austronesian speakers moving from the Philippines, and Papuan language speakers arriving from New Guinea[72]. These populations appear to have focused on hunting and gathering, with some landscape cultivation and reliance on trade[73]. Subsistence appears to have been centred around swiddening[73] and selective cultivation of sago (*Metroxylon sagu*) within low-lying swamplands[74]. Land clearance for cropping potentially resulted in forest opening. Historical accounts indicate that swidden cropping likely focused on *Coix, Cenchrus*, millet (*Setaria italica*), and *Sorghum*[75].

The endemism of wild cloves (*Syzygium aromaticum*) – a lucrative trading commodity within the Asian market (Arab-speaking countries, China, and India) – to the five "Spice Islands" west of Halmahera resulted in frequent contact between local populations and external traders from at least the 1430s, including those

from the Arab world. The Portuguese made landfall in Maluku in 1511 CE and established themselves at Ternate (the administrative capital under Sultanate of Ternate) between 1522 and 1575 CE[76]. While information on Iberian impact on the local Indigenous populations in the 16th and 17th centuries is fragmentary, it appears that conflict, disease, and forced relocations between islands led to localized population declines[77]. Dutch records indicate a ~17% reduction in Indigenous populations for Central Maluku (the then-capital of the clove-trade) between 1634 and 1674 CE[77].

European occupation of Maluku by Portuguese, Spanish, and Dutch colonists disrupted traditional land-use heterogeneously across the archipelago. The competition for the establishment of a spice monopoly in the 16th and 17th century resulted in clearance of rainforests in the small "Spice Islands" (as well as the Banda Islands in South Maluku), alternating establishment and decimation of clove plantations and, in some cases, massacre and enslavement of the local populations and the deliberate destruction of their local food sources, including coconut and sago palms[78]. A secondary impact of land-use intensification on spice-producing regions was heightened land-use pressure in proximal islands for resources such as sago, fuel, and timber[73]. The Spanish focused on cultivation of so-called "New World" crops, including maize and cassava (*Manihot esculenta*) in the drier, southern islands, and taro and sweet potato *(Ipomoea batatas*) in wetter, northern islands[73].

Micronesia

Mariana Islands - Guam

Occupation of the Mariana Islands by Austronesian speakers occurred by 3500 yrs BP[79,80], though earlier dates have been proposed[81], and the tempo and nature of the arrival of agriculture remains contested. Early archaeological sites within the larger, southern islands – Guam and Saipan –are coastal-oriented[80,82]. Erosional evidence indicating increased inland land-use dates from 170 CE [83]. By 1000 CE (the start of the *Latte* Period – named for the upright pillars of stone house posts, or *latte* [84]), local Chamorro populations had expanded, and human occupation was evident across most inland zones in the Mariana archipelago[85,86]. This is thought to have resulted in the establishment of an extensive trading system for fish, rice and tubers between inland "agricultural people" and coastal fishers[87]. There is also evidence for human occupation of the rugged, water-scare, isolated islands in the north Marianas from 1300 CE[88], potentially a defensive response to heightened climatic instability[11].

The nature and timing of human impact, including land-use cultivation through swiddening, in the Marianas is contested. Athens and Ward [81] draw on palaeoenvironmental evidence from a sedimentary record in Guam (Laguas Wetland – a site included in this study) to argue that swidden agriculture and land cultivation associated with human settlement between 950 and 350 BCE was significant enough to trigger a regional stable state shift from humid forest to the savanna/dry forest habitat that the island currently supports. This

is contested by Hunter-Anderson [89] who argues that origins of savanna in the Marianas are geo-climatic, and probably associated with the amplification of the ENSO in the late Holocene. Nonetheless, evidence of pre-Spanish cultivation and/or exploitation of inland resources (characterized as extensive rather than intensive[56]), includes use of bananas, *Artocarpus* spp (breadfruit), coconut, sugarcane, taro and *Alocasia macrorrhica* (giant taro), and yam as food staples[56,86]. It has also been suggested that soil treatment with lime and burned coconut shells may have been used to improve crop yield[56,90]. Early Spanish accounts of Guam refer to cultivation by "every able bodied person" of "farm plots in the hillside or jungle" indicating intensive land-use at the time of European contact[87].

The first Spanish contact with the Marianas occurred when Ferdinand Magellan made landfall in Guam in 1521 CE[56], though the island was not officially claimed by the Spanish until 1565 CE. From 1658 CE, the island served as a temporary stopover for Spanish ships travelling between the Philippines and Mexico[56]. While the Chamorro population was likely impacted by Spanish-transmitted diseases in the 1650s and early 1660s, Spanish settlement in 1668 CE served to significantly disrupt Chamorro culture and demography[56]. This was due to a combination of disease (including smallpox epidemics in 1688 and 1689), the implementation of missionization and *reducción* policies, and conflict between the Chamorro and Spanish[91,92]. The immediate result of this disruption was an estimated population decline of nearly 70% between 1668 CE (24,000 to 28,000 people) and 1699 CE (8100 people)[63]. In the case of the smaller Mariana islands (aside from Rota), these policies led to the complete destruction of the Chamorro[63]. In Guam, missionization and *reducción* had the effect of forcibly translocating Chamorro people into consolidated towns in southern Guam (Hagåtña, and Pago, Agat, Inarajan, Umatac and Merizo)[93]. It is notable to point out that reconstruction of population density from inland archaeological sites in Guam actually shows a significant decline in Chamorro population between 1400 and 1500 CE, prior to Spanish contact, suggesting that intensification of ENSO-associated climate extremes may have been an additionally important control on population size[85].

As well as the disease impacts of colonization, the Spanish introduced maize, pigs (*Sus scrofa*) and the Philippine deer (*Rusa marianna*) to Guam in the 1660s and 1770s, respectively[94,95]. Research on the contemporary ecology of Guam Karst forests suggests that while the former does not have a significant impact on forest regeneration, foraging by the Philippine deer decreases seedling and vine abundance, which may have had opened the forest after its introduction and proliferation on the island[96].

West Caroline Islands – Republic of Palau

The Republic of Palau is located in the West Caroline archipelago, and comprises over 350 islands distributed across 150 km. The climate of the region is mildly seasonal, and the island supports moist tropical forest[97]. Initial occupation of volcanic islands in northern Palau, including the large island of Babeldaob, is

generally accepted as part of the expansion of Austronesian speakers between 2550 and 1050 BCE[98-102], though this remains contested[103]. Smaller, marginally habitable, low lying, uplifted limestone islands in southern Palau termed "rock islands"[104] appear to have witnessed cultivation from ~1050 BCE[105], though evidence for permanent occupation dates from 700 to 800 CE[102]. It is expected that early settlers in Palau were reliant on subsistence crops (e.g. yam, arrowroot, bananas, Tahitian chestnut (*Inocarpus fagifer*), and breadfruit) in addition to the harvesting of marine resources[104,105]. Construction of significant inland earthworks, thought to have been driven by population expansion, began from ~50 BCE, and included step terracing for dryland agriculture[104]. Changes in land-use practices in Babeldoab occurred from 700 to 1200 CE, including increased reliance on wetland (pond field) vs. dryland agriculture for the cultivation of taro[104]. In the rock islands, agricultural activity is muted from 950 CE, and the islands were eventually abandoned between 1450 and 1650 CE, presumably due to climatic limitations imposed by the LIA[9,13], including reduced precipitation and a decline in near-shore marine resources[104-106].

By 1783 the population of Palau was estimated at between 20,000 and 100,000[107] (but likely in the range of 25,000 and 50,000[104,108]). At this time, many plants were actively cultivated, including fruit-bearing trees – *Cocos nucifera* (coconut), *Terminalia catappa* (tropical almond), bananas, breadfruit, *Eugenia malaccensis* (Malay apple), and root crops (yam, taro and *Cyrtosperma merkusii* (syn. *C. chamissonis)* (giant swamp taro))[104]. The hillsides of the main islands were terraced for taro production[11].

Though Spanish records indicate the sighting and naming of Palau in the 1500s, European contact with Palauans was relatively late (seasonally-unfavourable winds and currents thwarted several attempts made by the Spanish to reach Palau from Guam and the Philippines[109]). The first western accounts from the island derive from notes made after the British *Antelope* shipwrecked off Palau in 1783 CE[110]. There is some evidence that smallpox impacted population levels at this time[111]. Ship traffic to the islands increased after 1790[107], and Spanish sovereign claim over Palau was made in 1885. Smallpox acutely impacted the Palauans in the late 19th century, causing a population decline from 8,000 to 3,700 in just 30 years[112] (which, by ~1885 CE, had already represented a total Indigenous population decline of between 80% and 95% over a century)[107]. Venereal disease also played an important role in chronic reduction of the population between the late 1800s and early 1900s[112].

There is little data addressing the impacts of the short-lived Spanish period (1885 to 1899 CE) on the landscape. Under the subsequent German period (1899 to 1914 CE), Indigenous labour was exploited for the establishment of coconut plantations and phosphate mining, resulting in forest clearance that intensified during the Japanese colonial period (between 1914 CE and World War 2)[113].

West Caroline Islands – Yap

As with Palau, Yap experiences an annual dry season in the boreal winter and mean annual precipitation of less than 3,000 mm. This climate envelope can support either tropical forest or tropical savanna[114]. Currently, the "natural" vegetation of the island is seasonally dry tropical forest[115]. Humans occupied Yap from as early as 450 to 150 BCE[116]. The steep geography of the island means that the type of land cultivation deployed in the arable lowlands was not possible in the uplands[117], and thus agriculture on the island was probably locally intensive rather than regionally extensive. Cultivated plants include the giant swamp taro (which was potentially grown in reclaimed coastal mangrove sites), *Pandanus*, arrowroot, taro, yams, coconut, bananas and breadfruit[118]. Climatic disruption (heightened ENSO) at ~1300 CE in Micronesia is speculated from pottery remains to have led to temporary abandonment of the main island for the more defendable Fais and/or Lamotrek islands[11,119]. Nonetheless, oral histories related to human occupation of Yap indicate that, prior to the 1850s, the island was so overcrowded that people were pushed into marginal settings, including mangrove swamps[117]. The population at this time could have been as high as 51,000[80].

Though the Spanish and Portuguese sighted islands in the western Carolines in the 1520s, the Spanish only established the first mission in Yap in 1731 CE and claimed sovereignty over the region in 1874 CE. European visitation of Yap increased in the 1800s – driven by the expansion of whaling and trading (including increased demand for the island's sea cucumbers and copra resources)[120]. Demographic data for Yap State during the early European contact period are scarce. However, it appears that a significant decline of the Indigenous population was underway during the nineteenth century, driven by several waves of epidemics, including influenza, smallpox, tuberculosis and venereal disease[120]. By 1899 CE the Indigenous population was just 7,808[117]. If the speculated pre-European population of Yap is correct[80], this would mean a drop in population by as much as 85% over a century.

East Caroline Islands – Kosrae & Pohnpei

The East Caroline Islands, including Kosrae and Pohnpei, are exceptionally wet (mean annual precipitation >5000mm/year), and support ever-wet rainforest. Fluctuation in sediment input interpreted from total organic carbon analysis of swamp archives from Kosrae indicate human occupation by 550 to 350 BCE[121]. However, this interpretation needs to be approached with caution given that variable sediment influx into lake basins has been attributed to ENSO-associated climate extremes in Southeast Asian tropical lakes elsewhere[122]. By 40 BCE to 125 CE, there is evidence of large-scale clearance of island vegetation with fire[123]. The presence of breadfruit and giant swamp taro within the archaeobotanical record indicates intensive agroforestry practice on the island from 400 to 600 CE[123], though it is notable to point out that giant swamp taro is likely endemic to the region[124]. Between 800 and 1200 CE, an increase in population is argued to have resulted in the emergence of a hierarchical social system[125]. Climatic disruption at ~1300 CE in Micronesia – driven by heightened ENSO – resulted in the adaptation of agricultural systems in Kosrae.

This included: 1) the active conversion of coastal saltwater lagoons to coastal wetlands – the latter of which has higher agricultural potential[11,126], and; 2) the settlement of smaller defensible offshore islands (Lelu), which were altered by use of walled settlement and land reclamation through infilling of reef flats[11,126].

The earliest evidence for human occupation of Pohnpei is from 950 to 350 BCE, and by 270 to 670 CE heightened charcoal suggests the adoption of swidden agriculture (the hyper-wet setting of these islands precludes "natural" fire)[127,128]. Permanent agriculture is evident from 400 to 1000 CE[127,128]. Intensive development and use of the Nan Madol complex, comprising offshore islets of basalt and coral, occurred between 950 and 1450 CE and is linked to social stratification and population growth on the islands[129,130].

Initial European contact with populations on Kosrae and Pohnpei was late and sporadic relative to other islands in Oceania. This is likely due to the geographic isolation of the islands, the lack of conventional natural resources that could be exploited for profit[131], and fear of the local people – driven by the killing of two Portuguese and Spanish missionaries that ventured to the islands in 1710 and 1731 CE[131]. In the early- to mid-1800s, European-led scientific visits, whaling operations, and, later, the establishment of Christian missions resulted in outbreaks of influenza, smallpox, and dysentery on the islands[112]. This had a large impact on local populations, including a 90% crash (3,000 to 300) between 1830 and 1880 CE on Kosrae[132], and a 50% population decline (10,000 to 5,000) between the 1840s and 1900 on Pohnpei[112]. Introduced venereal disease also resulted in infertility in both Pohnpei and Kosrae in the years after Spanish arrival, impeding population recovery even into the 20th century[112].

A review of historical records suggests that in addition to mass mortality, Europeans introduced the domestic pig to the islands in the 1840s (Pohnpei, Kosrae, and Chuuk)[133]. However, European land-use interventions appear to have otherwise been minimal[131].

Marshall Islands

Pollen and starch samples from archaeological sites reveal cultivation of taro, giant swamp taro and giant taro within the Marshall Islands between 50 BCE and 350 CE[134-136]. In some cases, this occurs soon after atoll uplift, indicating active manipulation of a marginally habitable environment by people[137].

European exploration of the Marshall Islands commenced in the 1500s although, while the Spanish claimed them in 1592 CE, intensive European contact appears minimal. Early records of diseases from visiting ships appear sparse and Francis and Hezel [112] suggest that the delay of significant foreign contact resulted in a relatively late, but significant, population declined of 30% (13,000 to 9,267) between 1855 and 1908 CE (when the Spanish sold the islands to the Germans).

SPANISH AMERICAS

Mexico & Central America

Prior to Spanish contact in 1519 CE, the tropical lowlands of North and Central America were extensively cleared and cultivated for the production of maize (*Zea mays*) (a cereal that is thought to have been (at least semi-) domesticated 9000 year BP in the Central Balsas River Valley, Mexico[138,139]), tomatoes (*Solanum*), beans *(Phaseolus*), squash (e.g. *Lagenaria siceraria, Cucurbita argyrosperma, C. moschata* and *C. pepo*)., chili (*Capsicum* spp), cacao (*Theobroma cacao*), cotton (*Gossypium hirsutum),* cassava/manioc (*Manihot esculenta*), *g*arden crops and agroforestry[140-144]. At time of Spanish occupation, the key cultural groups in Mexico and central America included those incorporated within the so-called Aztec Empire (Triple Alliance) (1428 to 1521 CE), Purépecha (Tarascan) State (~1300 to 1530 CE) and, to a lesser extent, remnant Mayan populations from the Terminal Classic Mayan city states.

Recent estimates of the pre-Iberian populations of central Mexico and the Yucatán range from 3 to 52 million (centered on an average of 20 million)[32]. Estimates for Central America, including Belize, Costa Rica, El Salvador, Guatemala, Honduras, Nicaragua and Panama, range from 4.75 to 6 million people[32]. Median pre-Columbian land-use (ha per capita) has been estimated as 1.1 for Mexico, and 1 for Central America[32]. These estimates derive from consideration of the both the dominant land-use type (a type of swidden agriculture called *milpa* that involves field cropping with maize, beans, and squash) and estimated yield across the range of landscapes incorporated into these regions[32]. A 90% population decline over the 16th century is estimated for both Mexico (including the Yucatán peninsula) and Central America following the introduction of novel diseases by the Spanish in the 1500s[32].

Purépecha (Tarascan) State & Triple Alliance (Aztec) Empire

Management of the landscape via terracing and irrigation was a common feature of Tarascan- and Triple Alliance- controlled regions prior to Spanish contact. Given the diverse range of environmental settings within this zone, land-use techniques were variable across space. Landscape heterogeneity created by coastal hills and plains allowed for a diverse range cultivation types as well as agroforestry, resulting in mosaics of forest, scrub and more open landscapes[144].

The coastal lowlands of Veracruz – running along the Gulf of Mexico and extending into Mayan territory – were subject to human disturbance from ~3750 to 3000 BCE[142,145], including the cultivation of maize[18,146]. At the time of Spanish contact, this region was managed as a patchwork of garden, forest and farming plots, each developed in accordance with the local microclimate and site aspect[147]. Northwest of Veracruz, the steep slopes of the Sierra Madre Oriental were, at the base, heavily terraced, and, towards the top, subject to shifting cultivation[144]. A pollen record from Laguna Azteca shows that the region underwent periodic pulses of intensified human clearance between 280 and 1420 CE[148].

West of Veracruz, the west-east striking Tran-Mexican Volcanic Belt (which extends to the Pacific coast), hosts the Mexican Valley (now Mexico City)[149]. Here, large lakes within topographic depressions, including Texcoco – an island in which hosted the capital of the Triple Alliance, Tenochtitlán – were integral to the development and dominance of the Empire. These lakes were used for fish, shellfish and hunting waterfowl, and extensively modified using *chinampa* cropping. This agricultural system involved the construction of artificial islands with layers of vegetative material that were cultivated with maize, beans, squash, tomatoes, and chili peppers[150]. Lake waters were corralled into extensive canals systems that become a key mode of city mobility within Tenochtitlán[150].

Beyond the northwest margin of Trans-Mexican Volcanic Belt, the Purépecha (or the Spanish termed "Tarascans") – an enemy state to the Triple Alliance – occupied the more water-limited environments of the Mexican Plateau. Like the Triple Alliance, this state was also centered around a large lake called Pátzcuaro[151,152]. Regionally, forests were retained in montane regions whereas soils below the forest line were terraced for rain fed irrigation[144]. Canals and wetland irrigation systems were concentrated around plateau swamps and lakes, predominantly for maize production[144]. Pollen records from the Mexican Plateau indicate land-use intensification in the first millennium BCE[18].

'Maya' lowlands

The Yucatán peninsula was extensively cultivated prior to Spanish occupation and, as with much of the North and Central American tropics, maize was a staple crop for the region. Pre-Spanish land-use was, however, variable across space and time. The wetter central and southern parts of the peninsula (Central Maya Lowlands – comprising present day northern Petén, south Quintana Roo and Campeche in Mexico, and adjacent parts of Belize and Guatemala[153]) were heavily peopled during the height of the Classic Mayan civilization whereas the coastal margins and inland waterways of the peninsula became increasingly managed in the centuries leading up to Spanish occupation as part of Terminal Classic and post-Classic occupations[144,153].

At the time of Spanish contact, people relied on coastal wetlands for salt trading[144]. Palynological data indicate that humans were clearing forests and cultivating maize, cotton, and chili in this region from ~2500 BCE[154]. In additional to coastal marine resources, pre-Spanish societies relied on crops from gardens, orchards and agroforestry[144]. Commonly cultivated plants included cacao, chili, maguey (*Avage*), cotton, avocado (*Persea americana*), nance (*Brysonima crassifoli*a), allspice (*Pimenta dioica*), guava (*Psidium guajava*), sapodilla (*Manilkara zapote*) and mamey zapote (*Calocarpum mammosum*)[144]. Shifting agriculture (*milpa*) was a common feature along village margins, and was used for growing maize, squash and beans[144]. Beyond these swidden plots, forest "buffers" were used to separate villages, and for agroforestry, deer hunting, and fuel[144]. Pollen data for the North Yucatán peninsula within the vicinity of

Merida (Mayan city of T'hó) show heightened forest disturbance and agricultural indicators after 950 CE[155]. A small afforestation signal between ~1300 and 1450 CE, and hiatus in crop pollen may suggest decreased land-use over this time period[155].

At the time of Spanish contact, the central Maya Lowlands, southeast Mexico and Honduras were variably cultivated (predominantly for maize) using slash-and-burn techniques, though the extent of intensity of agriculture is expected be significantly lower than was present in the region during the Terminal Classic Period[144,153]. Pockets of seasonal, fertile zones extending along the Pacific coast, including Soconusco, were vegetated with cacao orchards, from which the Aztecs extracted tribute[144]. Significant terracing was conducted on hillslopes where various types of maize, squash, beans and tobacco (*Nicotiana*) were grown[144].

The forests across the Central Mayan Lowlands were likely more open (or perhaps more accurately, patchy) during the height of the Classic period[156], the southern cities of which "collapsed" between the 8th and 10th centuries CE. This "collapse" has been attributed to progressive exhaustion of forest resources superimposed by drought between ~750 and 1100-1150 CE and is thus postulated to be followed by forest recovery. However, pollen data paint a more complex picture. For instance, while some records from the Copan polity in Honduras indicate forest depletion in the Late Classical Period[157], others reveal much earlier deforestation (900 BCE and 400 CE), and afforestation from 400 CE, suggesting active management of forest resources[158]. Pollen records from Colha, in the central-east Yucatán, show forest clearance and agricultural intensification after ~240 CE, and afforestation after city "collapse" at~800 CE[154].

Central America elsewhere

The region south of the Mayan heartlands, including the Gran Chiriquí Archaeological Region, was cultivated using shifting swidden techniques, which, in places, transitioned to more permanent forms of agriculture after 1000 CE[159].

Caribbean

It is estimated that the pre-Spanish, predominantly Taíno and Kalinago population of the Caribbean comprised 300,000 to 500,000 people, and that median land-use per capita was approximately 0.73 ha – an estimate assuming similar land-use practices in the Caribbean to those deployed in Mexico and mainland Central America[32]. Semi-permanent and permanent agriculture was common across most of the Caribbean islands prior to European contact. This included cultivation of diverse food staples using fertilized raised plots (*conuco*), slash-and-burn techniques, and, in places (e.g. Puerto Rico) stone terracing[160,161]. Cultivation centered on maize (from as early as 3000 BCE[162]), as well as cassava and sweet potato[160,162]. There is also evidence for the cultivation of cotton, tobacco, chilis, achira (*Canna* spp.), legumes (Fabaceae), and yams (Dioscoreaceae), as well as use of marunguey (*Zamia* spp.).

There is marked evidence for significant environmental change in the Caribbean associated with pre-Columbian land-use. For instance, heightened erosion, fire activity and forest clearance for maize, tubers and squash cultivation is apparent in Puerto Rico by 1150 CE[160]. This is generally consistent with evidence for maize cultivation from two lake sites in the Dominican Republic from 1060 CE[163]. Pollen data from the Bahamas indicate a sharp decline in forest taxa at 1080 CE[164], just after the first evidence of human occupation (900 to 1030 CE)[165], implying human-influenced forest opening. Palaeoecological evidence for agriculture and landscape modification in Haiti dates from 850 CE[166].

It is estimated that 99% of the pre-Spanish populations in the Caribbean were wiped out[32] – a consequence of disease, massacre, and Spanish exploitation for slave labour. Examples of the large upheaval in population dynamics from the Greater Antilles (the large islands in the Caribbean) in the centuries following Spanish occupation are as follows.

Puerto Rico, Hispaniola (now Haiti and the Dominican Republic), and Cuba – islands that the Spanish valued for their natural resources (the first two of which were exploited for mining, while the latter was important for plantations) – were subject to particularly high levels of depopulation and population replacement via the slave trade. The population of Hispaniola and Puerto Rico was heavily impacted by disease outbreak in 1497 CE[167], and the remaining population was diverted into Spanish gold mines. This resulted in the decline of *conuco* agriculture, famine, and, by 1522 CE, the Indigenous population of both islands was greatly reduced[168]. The continued demand for labour in Puerto Rico and Hispaniola resulted in the import of 34,000 slaves to these islands from the different areas of the Caribbean and surroundings between 1492 and 1550 CE[169]. Recent research on the post-Spanish period in Hispaniola has emphasized the significant resistance of both African slaves and the Taíno to Spanish control[170]. This led to the establishment of "escaped" maroon communities within areas of the island that the Spanish struggled to control due to geographic factors, including the steep topography of the island[170].

In Cuba, which was settled by the Spanish in 1514 CE at Havana, early massacres of the local population were common[171]. Many of those who managed to escape to the mountains were captured, placed into reserves and attempts were made to force the captives to work the land. These efforts were largely unsuccessful because of disease, resistance to slavery, or escape. As with Puerto Rico and Hispaniola, the Indigenous population of Cuba was devastated in the early 16th century[171,172]. Slaves were brought across to the islands to work the sugar and tobacco plantations – a practice that continued until 1867 CE[171].

The Bahamas, which were of little interest to the Spanish in terms of natural resources, were exploited for slave labour. This resulted in the entire removal of the Lucayan (Taíno) population (~5,000-40,000 people) in 1490 CE, resulting in the island being completely[169] or significantly[173] depopulated by 1515 CE – a phenomenon that apparently persisted for the next 130 years[169].

South America

Amazonian basin (including Llanos de Moxos ecoregion)

Recent estimates of pre-Spanish population within the Amazon drainage basin range from 8 to 20 million people[32]. Median land-use for this region 1492 CE is estimated to be 1.23 ha per capita[32]. This number is derived from assumptions made about intensive cultivation of localized, fertile Amazonian Dark Earths (ADEs) (see below) and less intensive, but potentially extensive, long-fallow cultivation of less fertile non-ADEs for cassava/manioc.

Amazon rainforests (including Amazonian Dark Earths)

There are several reviews covering the current knowledge regarding pre-Columbian land-use practices in lowland Amazon forest[32,174-177]. In summary, humans have been adapting to- and manipulating- Amazonian resources for millennia through the adoption of polycultural agroforestry focused on cultivation (*Cucurbita* sp., maize, rice, cassava, peanut (*Arachis hypogaea*) and chili) and manipulation of wild forest tree species[178,179]. Intensification of land-use in the lowland Amazon Basin appears to have occurred from 4,500 cal yrs BP[180] to 3,400 cal yrs BP[181] and again at 500 BCE[180], marked by the formation of anthropogenic soils termed Amazonian Dark Earths (ADEs)[174,176,182,183].

ADEs, which are thought to occur across ~154 063 km^2 of the lowland Amazon Basin[184], are concentrated in well-drained, floodplain settings in the central, east and west Amazon Basin, and have been enhanced with charcoal, ash and other organic material[174,185,186]. From ADE distribution, it has been hypothesized that pre-Columbian human occupation of the Amazon was concentrated in seasonal forests, or within 15km of floodplains rather than in ever-wet forests or in interfluvial regions. However, recent modelling suggests that ADEs do, in fact, occur within interfluvial zones[185], suggesting broader scale human use of lowland Amazonian landscapes than previously thought.

Forest types found on ADEs are compositionally distinct from their non-ADE counterparts, representing higher abundances of domesticated and edible plants including fruit trees (Agavaceae, C*aryocar, Byrsonima, Theobroma cacao* and Lecythidaceae) and various palms (*Mauritia, Attalea, Astrocaryum, Euterpe, Elaeis*, and *Oenocarpus*)[174,177,187-189]. Beyond ADEs, less productive soils were used to cultivate cassava[32]. Geoglyphs used within Western Amazonia between 50 BCE and 1300 CE (and potentially earlier), provide evidence for human-driven manipulation of the landscape in the upper Amazon Basin, including patchy, low-scale forest fragmentation[176,190-192]. In the central and western Amazon, intensive agriculture was, in places, abandoned (or reduced) at around 1250 CE[181], though there is evidence for cultivation of an open landscape in the western Amazon-Andean corridor until 1600 CE[193]. This is potentially related to people moving into more fragmented, less permanent settlements as the climate transitioned from more- to less- humid during the late MWP[26].

Iberian settlers (then representing the joint Crowns of Portugal and Castile) progressively invaded the Amazon along the Amazon River corridor and its tributaries (where missions were established) in the 17th century, and the first Iberian territory in the region, the State of Maranhão, was established in the northeast in 1621 CE[194]. The Amazon "frontier" was, at that time, porous – being surrounded by French, English and Spanish territories, and Indigenous populations dramatically shrunk where contact with European groups occurred[195]. However, because of the size of the Amazon, waves of endemic diseases, including measles and smallpox, profoundly devastated and redistributed Indigenous populations well into the 18th century[177,196].

After establishing a stronghold over the region in the 17th and 18th centuries, the Portuguese introduced cattle raising and extracted lucrative "spices" from the rainforests (termed *drogas do sertão*), including cacao, vanilla, and sarsaparilla, among other tropical forest medicines, foods and resources[197]. The profitability of these enterprises and later, rubber production (*Hevea brasiliensis*), was maintained though compulsory labour and enslavement of Indigenous populations[194], a process that resulted in the translocation of Indigenous populations, and overhaul of many societies across the Amazon[196].

Llanos de Moxos

Llanos de Moxos (or Beni savanna) represents a distinct, seasonally inundated savanna ecoregion that is punctuated by forest on islands along river corridors and on anthropogenic mounds dating from the early- to mid- Holocene[198]. This region is well-known for its preservation of extensive archaeological features, including raised fields, habitation mounds (upon which forest can grow in an environment that is otherwise too waterlogged), irrigation features, fish weirs and ring ditches[199-203], and has been proposed as an important, early center of plant domestication[198]. The main pre-Hispanic dietary staples appear to have been maize, squash, sweet potato, peanuts[198], "New World" elephant ears (taro) (*Xanthosoma*), and cassava[204]. Tree cultivars on the forest mounds included *Inga*[205], cacao[206], annatto *(Bixa orellana)* and *Ilex*[204]

The archaeological features of the Llanos are heterogeneous across space. For instance, there is limited evidence for raised platform agriculture (a human adaptation for which the Llanos de Moxos is well known) in the so-called 'Monumental Mound Region'[203] (southeast Llanos). This zone is rather characterized by drainage ditches and anthropogenic mounds that are up to 20m high[203]. Ring ditches, canals and causeways are important features within the northeast Llanos de Moxos[207] (bounded to the east by tropical rainforest). In the north, raised agricultural mounds were key for feeding villages that were each populated with up to 2,000 inhabitants[204]. These archaeological discrepancies have been hypothesized to result in differential use and manipulation of the landscape as discussed below[207].

Within the ring-ditch region, it has been posited that the construction of large earthworks and palisades from tree trunks, potentially for defense purposes, necessitated clearance of forest, presumably from adjacent

forest ecoregions, mounds or river terraces[207]. Pollen and charcoal records from area indicate evidence for active human manipulation of the landscape with fire from ~250 BCE[201], including for maize production from ~150 BCE to 100 CE[201,208]. The timing of adoption and later reduction of fire management appears to be spatially heterogeneous. For example, decreased use of fire (and subsequent forest encroachment) occurs at 710 CE at La Luna [201], 1430 CE at Laguna Granja[208], and there is no clear evidence for human fire-forest interactions at the Oricore site – a site due just east of La Luna, which, given its large size relative to the other sites, likely picks up a regional rather than a local signal of vegetation change[209].

Palaeoecological data from Laguna San José – a large lake within the Monumental Mound Region shows high levels of anthropogenic burning between 400 and 1280 CE, but no evidence of forest clearance/recovery associated with human demographic and economic changes[210]. Pollen and charcoal sequences from two oxbow lakes proximal to the El Cerro raised fields in the Central Llanos de Moxos, indicate a similar shift from higher- to lower- anthropogenic fire activity at ~1300 CE[205] to that seen in the eastern Llanos de Moxos records. Human management of the landscape appears to have decreased in the years before Spanish contact (1450 CE), as interpreted from an afforestation signal. Data from Lago Rogaguado[211], a large lake lying ~300km northwest of Laguna San José, shows very early cultivation of maize (6,500 yrs BP). However, there is no clear impact of pre-Columbian land cultivation on forest cover[211].

Within the north Llanos region (including the archaeologically rich area of Acre, Brazil[190] and Madre de Dios, Peru[192]), phytolith and geochemical data from soil profiles imply careful forest management[190] or minimal forest disturbance[192] by pre-Columbian people, with no significant evidence of extensive land clearance. This is somewhat consistent with data from two lake sites from the upper Llanos de Moxos that stretch along the southwest savanna-forest ecotone[212]. Here, increased human fire management occurred heterogeneously – 550 BCE to 950 BCE at Chalalán and ~950 CE to 450 CE at Santa Rosa, a likely result of mobile and extensive swidden farming[212]. While burning had a moderate impact on forest composition, it did not result in replacement of forest with more open ecosystem types[212].

Early Spanish contact with the Llanos de Moxos and its peoples was relatively late (between 1583 and 1622 CE).First Iberian interventions involved the forced resettlement of Indigenous populations into Jesuit mission towns from 1660 CE[213], and simultaneous introduction of cattle to the region[214]. This resulted in a notable drop Indigenous population size due to introduced disease, and the replacement of dietary protein from wild game to beef and dairy products[214]. Indigenous population decline between the pre-Spanish and mission period in the Llanos de Moxos is estimated at ~70% (350,000 to 100,000)[215].

Los Llanos (Orinoco Llanos)

Los Llanos is a savanna ecoregion within the Orinoco River catchment bounded to the northwest by the Andes, and to the southeast by Amazonian tropical forest. Humans occupied the region from the mid-Holocene[216], and there is evidence for increasingly intensive human use of the landscape over the past 2,000 years. This included swidden farming, and modification of the landscape to promote crop (typically maize and cassava) cultivation using raised mounds, ridged fields, and causeways [199,217,218]. The development of palm forests (including *Mauritia*) in the late Holocene may be an indicator of agroforestry in the region[219,220]. As with the Amazon Basin, land-use is expected to have been relatively low in the interfluvial zones, and more intensive along river channels[221].

The pre-Spanish population of Los Llanos and the Orinoco Valley is unclear, though has been estimated at ~200,000 to 500,000 people[221]. Known pulses of disease outbreak occurred in the late 16th century, though estimated population decline in Venezuela appears lower than that of the surrounding ecoregion. Here, the Indigenous population is estimated to have been as high as 200,000 to 300,000 people at ~1600 CE[221]. Spanish land-use in Los Llanos was largely impeded by the waterlogged conditions. They did, however, rear cattle and clear for agriculture (including for palms) in the 1500s[221]. In the 1600s, animal husbandry and agriculture intensified, and sugarcane, tobacco, cacao and coffee were cultivated in the Orinioco watershed[221].

Brazilian Cerrado

The role of climatic versus anthropogenic forces in the origins of the infertile, resource-poor scrub savannas (cerrados) of the central Brazilian Plateau has been debated. However, it is generally accepted that they were formed from climatic drivers[222]. In the late Holocene, sedentary, diverse Indigenous populations used cerrados for hunting, gathering, and seminomadic, swidden farming of maize, pumpkins, beans, cassava, and native fruits (*Caryocar brasiliense; Byrsonima crassifólia; Mauritia flexuosa; Hancornia speciosa*)[223],[224,225]. There is some evidence for the artificial creation of forest patches (*apetê*) using soil from termite mounds mixed with mulch[226], and more concentrated agricultural activity in riverine gallery forests and forest islands[199]. Additionally, it has been hypothesized that the marginal, beta-diverse ecotones at the cerrado-rainforest boundaries were subject to heightened disturbance, including clearance by fire[199]. This may have resulted in cerrado-creep into "natural" rainforest regions[199]. It is contended that population densities in the Brazilian Cerrado relate to climate drivers, with more arid conditions (e.g. the mid-Holocene dry period) and/or climatic instability leading to low populations until ~2,000 yrs BP[20,227].

Disease outbreaks within the Brazilian Cerrado likely occurred after settlement along the Atlantic coastal zone in the 1550 and 1560s. This, coupled with enslavement, is expected to have led to large population declines[111,228], though exact numbers have not been explicitly estimated for the Cerrado. It is possible that

there was Indigenous immigration to the cerrados from the Atlantic Forests as the Tupí attempted to escape Portuguese genocide in the coastal lowlands (see below section)[147]. Portuguese interest in using the Cerrado was limited due to its perceived lack of natural value[223].

Atlantic Forests

The Atlantic Forests – extending along the Atlantic coast of Brazil and biogeographically isolated from the Amazon rainforest by the Brazilian Cerrado and Brazilian Caatinga ecoregions (Fig. 2b) – witnessed human population increase and intensified land-use from 540 and 1050 CE[229]. This is attributed to the onset of more humid climate conditions in the region, permitting expansion (including human-mediated proliferation[230]) of Araucaria forest which provided a useful resource for people[229]. The timing of this climate shift coincides with population turnover associated with the Tupí invading from the west and assuming control of the region[147]. Pre-Iberian agriculture included cultivation of maize, cassava, beans, squash, sweet potatoes, peanuts, tobacco and cotton[147].

The Portuguese first landed in the Atlantic Forests in 1500 CE, and established colonies in 1530s at São Vicente and São Paulo[147]. Early changes to the landscape were pronounced, and involved overharvesting of forest resources, including the brazil nut tree (*Caesalpinia echinata*) other hardwoods, and ipecac (*Carapichea ipecacuanha* – a medicinal shrub); clearance for cattle raising and farming, including establishment of sugar cane and tobacco plantations, and; later, gold and diamond prospecting within Minas Gerais[147]. As with the rest of South America, diseases brought across by the colonists triggered loss of Indigenous life in the region in the mid-1500s. There are records of major smallpox epidemics in 1555 and 1563 CE, the latter of which spread from the coast to the inlands along river corridors[111], the plague, dysentery and measles[147].

In addition to disease impacts, the Portuguese actively engaged in the murder of the Tupí people after settlement, which had significant implications on Indigenous population size (many people were killed) and structure (those that were not killed fled or were forced into Jesuit missions)[147]. Survivors who managed to escape to the interior likely substituted pre-Iberian horticulture for more flexible and mobile food procurement via hunting and gathering[147]. By the 1570s, the Pre-Iberian Indigenous populations, once estimated at ~1 million, were reduced by at least 66%[147], and near entirely eliminated by the end of the 18th century[231].

Pacific Forests

The Pacific Forests are wedged between the Pacific coast to the west and the foothills of the Andes to the east (Fig. 2b). Pre-Spanish populations in the region relied on coastal and marine resources for protein[232]. Away from the coast, terrestrial land-use likely merged with the agricultural practices deployed in the lowland Andes (described below). Human impact in the region is evident from ~250 CE, which includes

cultivation of squash, cassava and, potentially, palm forests[219,233,234]. In the southern Chocó-Darién forest ecoregion – an extensive pluvial rainforest along the Pacific Coast of Columbia renowned for being one of the wettest places on earth – raised fields were used for agriculture to improve the quality of the water-logged soils[233].

Spanish interest in the Pacific Coast for mining of placer deposits increased in the mid-17th century and involved the enslavement of Indigenous Americans and imported African slaves. This resulted in many transient and some more permanent mining towns being constructed along river corridors including at Upper Chocó (North Colombia) and Barbacoas (South Colombia), the latter of which was linked to coastal settlements within the southern Pacific Coast Forests (Iscuandé, Guapí, and Tumaco)[235]. It is, however, notable to point out that early attempts by the Spanish to mine placer gold, including at the Tamaná and Upper San Juan rivers near Nóvita in Upper Chocó were met with fierce resistance by local Indigenous populations, and failed[236,237].

While local Indigenous populations were often forced to procure food for the mine workers[238], the extreme wetness of the Pacific Forests meant that the Spanish had little control of (or apparent desire to control) the region outside of the Upper Chocó and Barbacoas mining regions[236]. Most Spanish mine overseers preferred to settle in the more comfortable Andean uplands to the east[236]. As a consequence, areas in the Pacific forest became escape havens (e.g. Esmeraldas in the southern Ecuadorian Pacific Forests which grew after the grounding of Spanish slave ships in 1553 CE[235]) or sites of resistance. Here, escaped slaves and peasants cultivated maize and "Old World" crops (taro, sugar cane and breadfruit) on river levees for subsistence activities and markets[239]. Nevertheless, Spanish disease and attempts to control the region eventually led to large loss of Indigenous life. It is estimated that the populations within the Upper Chocó dropped from 35,000 to 3,850 people between the late 16th and late 17th centuries[236].

Andes

The central and northern Andes (i.e. sub/tropical Andes) support an extremely diverse range of environmental gradients. Very broadly, these can be split into four montane habitat categories that are relevant for discussing pre-Spanish land-use[232]. These are outlined below.

Constrained, lowland, seasonally dry forest pockets within riverine valleys carved into the Andes – including the Patía and Cauca Valleys in the North Andes – were exploited for placer gold and agriculture, the latter of which involved patch clearance and irrigation agriculture for arrowroot and maize[224]. Cultivation within dry forest valley sites commenced from ~4,000 cal. yrs BP and appears to have reduced in intensity at ~1000 CE, the reasons for which remain unclear[219].

The foothills running along the western and eastern flanks of the Andes (<2,300 m ASL) represent the lowermost "montane" zone. Though the agricultural potential of the water-limited western slopes is

constrained to valley settings, the eastern slopes, overlooking the Amazonian basin, receive plentiful rainfall and were home to human populations undertaking crop cultivation, foraging, and arboriculture, which may have included cherimoya (*Annona cherimolia*), papaya de monte (*Carica pubescens*), pepino (*Solanum muricatum*), tree tomato (*Cyphomandra betacea*), lúcuma (*Pouteria lucuma*), pacae (*Inga Feuillei*), ciruela de fraile (*Bunchosia armeniaca*), guava (*Psidium guajava*), achiote (*Bixa orellana*) and avocado (*Persea americana*)[224,232]. This zone appears to have been cultivated over the past ~5,000 years[219].

The mid-montane zone (2,300 to 3,500 m ASL) hosts fertile land and was thus heavily terraced and cultivated (sometimes using irrigation agriculture) for a range of crops[232]. These include maize, achira (*Canna edulis*), llerén (*Calathea allouia*), arrowroot (*Maranta arundinacea*), cassava, sweet potato, squash (*Cucurbita moschata*, *C. maxima* and *C. ficifolia*), chili, cacao[224] and palms (including *Acrocomia*)[224].

Crops suitable for cultivation above 2,300m include chenopods (quinoa (*Chenopodium quinoa*) and cañihua (*Chenopodium pallidicaule*)), amaranth (*Amaranthus caudatus*), lupin (*Lupinus mutabilis*), fava beans/haba (*Vicia faba*) and tubers (oca (*Oxalis tuberosa*), olluco (*Ullucus tuberosus*), mashwa (*Tropaelum tuberosum*), jicama (*Pachyrrhizus ahipa*) and potatoes (*Solanum tuberosum*))[224,232]. Specific landscapes within this zone, including the shores of Lake Titicaca, were heavily modified with raised fields for agriculture between ~500 and 1,000 CE[232,240].

Prior to Spanish contact, the Andean zone supported large populations that were sometimes linked through complex agricultural economies, including the Inka Empire in the central and south[224]. Populous regions within the northern Andes (which, being closer to the equator, has a higher proportion of forest at higher latitudes than the Southern Andes) included the Cauca Valley, Sabana de Bogotá, and Valley Alto Magdalena/Tierrandentro [241]. Humans within these regions engaged in intensive agriculture and metal mining[233].

Sedentary agriculture was established in the Cauca Valley after ~1,000 BCE, and involved landscape alteration including forest clearance, and construction of raised field mounds and drainage ditches[233]. Though there was social reorganization between 500 and 800 CE, large populations cultivated the area right up to Spanish contact[233].

The Sabana de Bogotá region was occupied by the Muisca – a complex confederation of chiefdoms – and comprises fertile uplands within the Colombian Andes (Fig. 2a). At the time of Spanish contact, the economically and politically powerful Muisca engaged in metal, gem and rock salt mining and intensive agrarian systems, including terrace and irrigation farming.

Large Indigenous populations inhabited the Alto Magdalena/Tierrandentro region (south of Sabana de Bogotá) and supported a high population density throughout the Classical Period (200 BCE to 900 CE; 22

and 44 persons per km^2 respectively)[233]. Despite these high populations, land-use under the intensive agricultural systems deployed in the region appears to have been under carrying capacity, resulting in the selective preservation of forest and fertile soils[233]. Post 900 CE abandonment of burial mounds and statues in the region is attributed to changes in sociopolitical organization (under a continuously high population), and the so-called "Recent Period" is associated with intensified agricultural systems including the construction of drainage works[233]. In additional to agriculture, Alto Magdalena/Tierrandentro societies engaged in metal and salt mining[233].

Spanish occupation and development of the Andes during and after the conquest of the Inka Empire (1532 – 1572 CE) – one of the most important Spanish campaigns in the region – focused on the control, privatization, settlement and development of the basin valleys[242], and concentrated exploitation of precious gem, metal, and silver extractive (e.g. mercury) materials[241]. Outside of the key mining sites, the landscape model applied to the Andes by the Spanish was one where uplands were seen as having "fugitive qualities" and were approached within land-use policies as empty spaces devoid of settlements and agricultural lands[242]. This is despite the fact that these highland zones were subject to high levels of pre-Spanish Indigenous agriculture (see above). As a result, a large focus was placed on urbanization and the resettlement of Indigenous populations into valley settlements (though this policy was met with a significant degree of resistance)[242].

Spanish contact had a big impact on reducing the Indigenous population of the Andes through disease (smallpox, measles, typhus, and influenza), brutal exploitation of workers for the mining industry, and fragmentation of the food supply[111,242]. This resulted in a population loss of between 50% and 80%[241,243]. The combined pre-Spanish population of the Andes and Pacific lowlands in the 1400s was estimated to be as high as 11.5 million people[243]. By 1620 CE, this population was estimated at 600,000[243]. The high value of the metal and gem deposits in the Andes, coupled with the large loss of Indigenous life meant that inter-Andean dry forest valleys (including Cauca) became centres for Indigenous and African slave communities, resulting in large-scale population turnover and social reorganization in the centuries following Spanish occupation[235].

Supplementary Text 3: Analysis of Iberian-induced ecosystem change (including site-by-site analysis of the pollen records included in the study)

This section, split into major geographic then ecoregional zones, provides a detailed analysis of the 28 Neotropical pollen records and 19 Asia-Pacific pollen, charcoal and phytolith records used for this study (listed and described in the Supplementary Data for this study). This includes:

1. Classification of pre-Iberian (1000 to 1500 CE) and post-Iberian vegetation and fire change following criteria detailed in the methods section of the manuscript.
2. Discussion of this change in the context of the original authors' interpretations.
3. Consideration of the viability of our interpretation in the context of the chronological models produced for each record. While the records extracted from Neotoma (i.e. the Spanish American sites) have up-to-date chronologies based on Bayesian models[244,245], some of the chronologies for the records extracted from the Spanish East Indies required updating. Where this was the case (and was possible based on the publication of the ^{14}C ages and errors), we remodeled the chronologies using the program Bacon[246]. In these cases, we provide a closer interpretation of the resulting age-depth model.
4. Analysis of our interpretations against other published pollen or palaeoecological records from each ecoregion that are not made available on Neotoma to assess (in)consistencies in our observations across space, and, where heterogeneities occur, consideration of why this may be the case. Given that we included all of the temporally relevant Spanish East Indies pollen records that we could find, this is only provided for the better-studied Neotropics.

SPANISH EAST INDIES

Northern Taiwan

Duck Pond[247] is located ~10 km inland from the Spanish colonial base Fort San Domingo (Fig. 1). The GAM that was run on the herb pollen percentages from this site (here taken as an estimate of changes in non-arboreal pollen) shows gradual forest opening from 800 CE (the start of the record) to a peak at the colonial Spanish-Dutch transition period between ~1633 and ~1740 CE (Fig. 5). Because this overarching trend represents a slow vs. rapid response to the onset of a cooler climate, high non-arboreal percentages at 1633 to 1740 CE appear to correspond to the onset of the LIA in the region. Vegetation change is thus interpreted as reflecting a climatic signal and is not classified as showing a floristic response to European colonization (Fig. 4). This interpretation aligns with those posed by the authors of the record[248].

We created an updated chronology for the Duck Pond record using the mean age-depth model predicted from 5 published ^{14}C dates[247] with the core top age set at -57 cal BP. The model was run using the following parameters: d.min =0; d.max=30; acc.mean=50; cc= IntCal13[cc1]; prob=0.95. Though the predicted age-

ranges for the dated points are broad (on the order of ~500 years), the model does show a near-linear age-depth model, and the mean model produces a reasonably well-constrained chronology for the record (Supplement Figure 2).

Dahu Lake[10] is located ~35km south of the former Spanish port at Keelung (Fig. 1) in what today is the coastal city of Yilan. The pollen PC1 data for this site (where negative loading corresponds to increased grass pollen) show, as with Duck Pond, decreased forest components between ~500 and 1300 CE (Fig. 5). Abrupt afforestation is apparent between 1300 to 1380 CE, which the original authors attribute to agricultural disruption due to extreme climate events (including flooding associated with heightened ENSO)[10]. The rapid deforestation signal at 1630 CE, coinciding with Spanish occupation (1626 to 1641 CE), is associated with an increase in cultivated taxa and thus clearance of the forest for agricultural activity[10]. This record has thus been classified as showing a pre-European afforestation signal, and deforestation associated with European colonization (Fig. 4). The chronology for Dahu lake is well constrained over the past 2,000 years (^{14}C samples = 4, ^{210}Pb samples = 10; no outliers/reversals)[10].

The Philippines

Palaeoecological data from ***Lake Paoay***[60] – a coastal site within northwest Luzon – is of limited use to this study as the sequence terminates just prior to (charcoal), or just after (pollen) Spanish occupation of Vigan in 1572 CE, the closest colonial settlement to the site. However, this site was included in this study due to the lack of other data from the Luzon (or the Philippines more broadly) – host of the capital of the Spanish East Indies, Manila, between 1571 and 1898 CE. Grass:AP data for Paoay indicate gradual opening of the forest from 50 BCE (Fig. 5). The most open landscape occurs at 1640 CE – the only post-Spanish settlement sample included in the dataset. This record has therefore been classified as showing a deforestation response to Spanish contact (Fig. 4), though more data is needed to clarify this interpretation. Interestingly, charcoal data from Paoay show that decreased fire activity occurs alongside forest opening from ~1000 CE, potentially related to a decrease in available biomass for burning, or a change in land-use strategies in the region. The chronology of the Paoay Lake sequence is well constrained over the past 2,200 years (n=5; no outliers/reversals)[60].

An unpublished charcoal dataset (prepared by J. Stevenson) from a 448cm-long sediment core from **Bululacao** – an upland lake surrounded by tropical cloud forests in North Luzon – shows a markedly different trend to the Paoay record. Here, fire activity in the landscape increases after 1000 CE, including after Spanish occupation of Luzon, and the record has been classified as showing a post-colonial increase in landscape burning (Fig. 5). This hints at asynchronous use of upland (Bululacao) vs. lowland (Paoay) resources in northern Luzon over the past 1,000 years. Ten organic macrofossil samples (flowers, buds, and leaves) were analysed using AMS 14C dating at the Australian National University Research School of

Earth Science to establish a chronology for the Lake Bululacao sediments. The depth, pretreatment and ^{14}C ages returned from these samples are presented in Supplement Table 3. In addition to the ^{14}C samples, 20 ^{210}Pb samples were analysed to constrain the age of the upper 20cm of the core sediments. The ^{14}C and ^{210}Pb ages were used to develop an age depth model for the core using Bacon[249] in R[250]. The model was run using the following parameters: d.min=0; d.max=430; acc.mean=10; cc=IntCal13[cc1]; prob=0.95. The resultant chronology is moderately well constrained over the past 2,000 years (^{14}C n = 3; ^{210}Pb n = 20) (Supplement Figure 3).

Wallacea

North Sulawesi (Minahasa)

A pollen record from ***Lake Tondano***[251] in North Sulawesi provides some insight into environmental dynamics associated with Iberian contact and Spanish occupation. Grass pollen and charcoal data indicate gradual forest opening between ~500 and 1470 CE, potentially associated with increased human activity in the catchment forest[251] (Fig. 5). There is no clear forest closure or decrease in fire activity associated with Spanish colonization of the region (which ramped up between 1606 and 1616 CE), or in the 600 years preceding Spanish occupation (Fig. 4). The pollen and charcoal records from this site have thus been classified as showing limited changes in afforestation and burning within the pre-Iberian or Iberian period (Fig. 4). The chronology of the sediment sequence from Lake Tondano is poorly resolved over the past 2,000 years (n=1)[251]. This is not considered to be particularly problematic in the context of the "no response" interpretation made for the record between 1000 and 2000 CE.

Maluku

Changes in grass pollen abundance from the ***Kao River Plain*** in Halmahera indicate forest expansion from 1200 CE (Fig. 5). This vegetation shift appears to coincide with decreased fire activity at the site, though this is not captured in the Kao charcoal GAM (Fig. 5). We have thus classified the pollen record as showing a pre-Iberian (pre 1511 CE) afforestation response, and the charcoal record as showing no response within either the Dutch-Iberian or pre-European period (Fig. 4).

We created an updated chronology for the Kao record using the mean age-depth model predicted from two published ^{14}C dates[251], with the core top age set at -45 cal BP. The model was run using the following parameters: d.min=0; d.max=1325; acc.mean=2; cc=IntCal13[cc1]; prob=0.95. The resultant model shows large uncertainty in the ages of sediments older than 1,000 cal BP (Supplement Figure 4). Due to the lack of chronological resolution for this record, we are cautious in our interpretation of pre-Spanish afforestation, but do note the 300-year discrepancy in the estimated timing of this change (1200 CE) and the timing of Iberian contact (1511 CE). Additionally, one of the two samples used in the model was dated at 410 ± 110 ^{14}C years, meaning that there is more certainty in the age-depth model around the timing of Iberian contact.

Micronesia

Mariana Islands - Guam

Records showing floristic response to colonization from the Marianas are sparse, and those that do exist are of insufficient temporal resolution to clearly ascertain landscape change over the past half millennia[252-255]. Nonetheless, a sequence from ***Laguas Wetland*** (IARII) in Guam[256], shows that grass pollen (taken to be an indicator of savanna) and charcoal decline at ~1650 CE. This implies increased forest cover after Spanish visitation and settlement on the island. Post-Iberian afforestation is however, classified tentatively for three reasons. First, the sampling resolution is insufficient for this shift to be captured in the pollen and charcoal GAMs produced for this core (Fig. 5). Second, the chronological model for Laguas (IARII) is poor over past 2,000 years (n=1 [260.5cm; 1,804±59 ^{14}C years]). Finally even if the chronology is reliable, the sampling gap between 1200 (low forest cover) and 1640 CE (high forest cover) means that afforestation may have preceded Iberian contact[256].

West Caroline Islands – Republic of Palau

Three records from Babeldaob – ***Lake Olbed*** (pollen and charcoal), ***Lake Ngerdok*** (pollen and charcoal), and ***Lake Ngardmau*** (charcoal only) – provide some insight into landscape change following Spanish contact in the late 1700s[98,257]. Grass pollen from Ngerdok, and charcoal data from all three sites indicate negligible shifts in fire and forest composition following Indigenous population decline between the 1700 and 1900 CE. The pollen data from Olbed do, however, show an afforestation signal after Spanish contact, though this shift is only reflected in a single sample modelled at 1780 CE, and thus is classified as showing post-Spanish afforestation with high uncertainty (Fig. 5). GAMs produced for the Olbed and Ngerdok pollen and charcoal records, which do not pick up data variability due to low sampling resolution, indicate progressive afforestation in the area over the past 2,000 years (Fig. 5). These records have been classified as showing a pre-Spanish afforestation signal (Fig. 4). The dates used to construct the chronologies for the Palauan palaeoecological records were not published in the paper from which the pollen and charcoal data was extracted[104]. Consequently, we are unable to assess the reliability of the chronological models produced from any of these sites.

West Caroline Islands – Yap

Grass pollen data from two dry forest sites on Yap – ***Fool Wetland*** and ***Thool Wetland***[258] – indicate afforestation after ~1700 CE. Both records show a decline in grass pollen after the island population was decimated due to the epidemics of 1850 to 1870 CE. This is captured in the Fool GAM curvature, but not the Thool GAM curvature (Fig. 5). Consequently, post-Spanish afforestation has been classified as uncertain/minor for Thool (Fig. 4). Charcoal data from Fool indicate that grass decline is consistent with a reduction of charcoal at the site, implying population decline. However, this is classified as minor/unclear

as the shift is not captured in the GAM produced for this record (Fig. 4). In addition to post-Spanish afforestation, a decrease in the abundance of grass pollen occurs at both sites between 1100 and 1350 CE – shifts that have been classified as indicating increased forest cover prior to Spanish occupation (Fig. 4).

We created updated chronologies for both the Fool and Thool wetlands. For Fool, we used the mean age-depth model estimated from two published ^{14}C dates[258] (240 ±50 ^{14}C years at 130 cm and 3340 ±80 ^{14}C years at 230 cm) with the core top assigned the age of -45 cal yrs BP. The model was run using the following parameters: d.min=0; d.max=335; acc.mean=20; cc=IntCal13[cc1]; prob=0.95. The resulting model rejected the younger ^{14}C age, favouring a linear age-depth profile. The chronology for the site is thus very poorly constrained over the past 2,000 years (Supplement Figure 5). We also used the mean age-depth model for Thool, which was estimated from three published ^{14}C dates[258] (140 ±70 ^{14}C years at 125 cm, 260±60 ^{14}C years at 215 cm and 2320±60 ^{14}C years at 330 cm) with the core top assigned the age of -45 cal yrs BP. The model was run using the following parameters: d.min=0; d.max=330; acc.mean=5; cc=IntCal13[cc1]; prob=0.95. The resultant age-depth model produced from this analysis excluded the middle ^{14}C date, and is thus poorly constrained (Supplement Figure 6).

East Caroline Islands – Kosrae, Pohnpei, Chuuk

The resolution of the pollen and charcoal records from wetland sediments in Pohnpei and Kosrae are too poor to infer floristic response to depopulation in the 1880s[121,123,124]. Though only slightly better resolved, charcoal data from an archaeological site (***PoC3***) at Pohnpei (Temwen) show a decline in fire activity after 1840 CE, simultaneous to disease introduction. This implies population decline and has been interpreted as a post-Spanish decrease in fire activity (Fig. 4). Ethnobotanical data from the same site show an increase in soil disruption (likely by pigs) and subsequent grassland expansion over the past 150 years[133], and have been interpreted as a deforestation response after Spanish contact (Fig. 5). Interestingly, these impacts are contrary to the afforestation that would be expected from disease-induced depopulation, potentially highlighting the important role of invasive species in controlling floristic response. The chronological resolution of PoC3 is moderate, with two dated samples over the past 1,000 years[133].

SPANISH AMERICAS

Mexico & Central America

Purépecha (Tarascan) State & Aztec Empire (Triple Alliance)

Study datasets

Lake Patzcuaro[142] is the only site from central Mexico that meets the selection criteria for inclusion in this study. This site, located northwest of the region occupied by the Triple Alliance, was the center of the capital of Purépecha prior to Spanish occupation[151]. NAP:AP data from Lake Patzcuaro record low values between 1500 and 1600 CE – a pattern reflected in a slight negative curvature of the GAM (Fig. 3). This implies possible afforestation following Spanish conquest and disease outbreak (~1521 CE), and has been classified as such in previous research[32]. However, the plant functional grouping (PFG) record shows remarkable stability in the full assemblage over the past 2,000 years (including the persistence of forest throughout), and breaks identified in cluster analyses of the data are weak (sum of squares <100) (Supplement Figure 1 – also included as an Extended Data figure for this paper).. The patterns reflected in the NAP:AP data are seemingly exaggerated, and thus afforestation response to depopulation is classified as minor for this site (Fig. 2). The chronological control for Patzcuaro is poor over the last 2,000 years (n=0). The youngest age sample measured for this core is 2890 yrs BP at 821.5 cm (Supplement Figure 6).

Supporting palaeoecological data

One of the issues with interpreting forest response to depopulation within Mexico and Central America lies in the fact that few pollen and charcoal records occur within the core region of the Triple Alliance or Purépecha Empires – the dominant regional powers prior to Spanish conquest. It is perhaps for this reason that none of the other pollen data from the region show an afforestation response to Spanish-induced population decline (which, as with other supporting data described in this section, were not included in this study due to either very poor data resolution or lack of data accessibility). For example, records from the Valle de Santiago of Guanajuato, which in the 16th century was populated with semi-nomadic Indigenous populations, do not show an afforestation response to Spanish conquest[145]. However, the response dynamics between sites in Guanajuato is interesting, with one record (Hoya San Nicolás) revealing a muted floristic response to Spanish occupation, and the other (Hoya Rincon de Parangueo) showing a definite increase in agricultural activities at this time[145]. In the case of Laguna Azteca – a Sierra Madre Oriental site – an afforestation signal is apparent from 1420 CE, a century prior to the Spanish conquest of the region[148]. A pre-Spanish afforestation signal is evident in pollen data from Laguna Pompal[259] – a site located in Veracruz at the intersection of Triple Alliance and Mayan territories – though in this case forest recovery occurs earlier, at 1300 CE. This record also shows heightened, rather than reduced, forest disturbance and fire

activity after Spanish arrival, possibly associated with the Spanish interest in the area for mining after 1518 CE.

Maya

Study datasets

Two sites – Cobweb Swamp[154] and Cenote San Jose Chulchaca[155] – were analyzed from the Mayan region. The former represents a Classical Mayan site near the Colha archaeological site north of Belize city, and the latter a north Yucatán site in the vicinity of Merida (previously T'hó).

The NAP:AP data from the **Cobweb Swamp** record are insufficiently resolved to produce anything other than a linear GAM showing gradual afforestation over the past 2,000 years (Fig. 3), suggesting limited abrupt forest change in response to Spanish occupation (or the "Classical" collapse). However, the PFG data quite convincingly show the replacement of an upland forest first with wet trees (mangroves) at ~1500 CE, and later lowland tropical dry forest trees Supplement Figure 1. This change is coincident with Spanish occupation, suggesting a degree of vegetation change associated with declining or changing human land-use (Supplement Figure 1). This represents the largest cluster break in the dataset (sum of squares = 250) (Supplement Figure 1). However, given the location of the site, the record is sensitive to shifts in sea level, which rose at the start of Little Ice Age at ~1450 CE[260], likely led to mangrove encroachment at the site, and can account for the increased wet tree pollen. The chronology for Cobweb Swamp is not very well constrained over the past 2,000 years (n=1) (Supplement Figure 8). We do note that the dated point of 536 yrs BP at 39cm depth is reasonably well-positioned for interpreting pre- and post- Spanish impacts (Supplement Figure 8). The uncertainties imposed by the chronology and sampling resolution of the data coupled with the mixed climate-human signals in the record mean that an afforestation response to Spanish-induced depopulation has been classified as minor/uncertain for this site (Fig. 2).

The **Cenote San Jose Chulchaca**[155] record shows a peak in NAP:AP coincident with the period ~1450-1600 CE, suggesting heightened landscape disturbance prior to and after Spanish occupation. This is coincident with a rise in dry forest (including secondary forest) taxa in the PFG record (Supplement Figure 1). Clustering of the pollen data show that the largest change in the PFG assemblage occurs prior to Spanish contact between 1420 and 1460 CE (sum of squares = 250) – a change representing an increase in lowland tropical forest taxa. This suggests pre-Spanish afforestation, but the chronological uncertainty means that this shift may have occurred post-contact. The chronology for Cenote San Jose Chulchaca is moderately well constrained over the past 2,000 years (n=3) (Supplement Figure 9).

Supporting palaeoecological data

While most datasets from the Classical Mayan heartlands reveal extensive pre-Spanish modification of the landscape, few show a consistent afforestation response to Spanish-caused Indigenous depopulation. Indeed,

an afforestation signal at these sites tends to more strongly reflect the human-environment dynamics associated with Mayan environmental management and changes in settlement patterns[18] than it does Spanish depopulation. For instance, pollen records from the Copán polity show that any signal of disease-related afforestation is muted by earlier (i.e. pre 900 CE) increases in forest cover (be they attributable to the collapse of this urban centre[157,261] or active forest management policies implemented by the ancient Mayans[158]). However, there does appear to be slight afforestation evident in the Petailla Swamp record after 595 ^{14}C yr BP (no age-depth model is presented for this core) which may relate to population decline linked to Spanish introduced-disease[261].

Central America elsewhere

Palaeoecological records within the area of Central America south of the Mayan region are sparse and none were available/eligible for inclusion in this study. A record from Laguna Zoncho, within the Gran Chiriquí Archaeological Region, shows a decline of agricultural activity in the ~200 years prior to Spanish occupation of the region[262]. This is attributed to the pressure that droughts imposed upon the pre-existing agricultural systems[262], perhaps consistent with pre-Spanish afforestation signal observed in the Cenote San Jose Chulchaca[155] and Laguna Pompal[259] records.

Caribbean

As Spanish land-use across the different islands was dependent on their perceived natural resource value, the impacts of the decimation of the Indigenous population on the landscape are expected to be variable across the region. For instance, population replacement though imported slaves (and continued land exploitation) occurred within Puerto Rico, Hispaniola and Cuba, whereas land cultivation in other islands (e.g. the Bahamas) was completely stopped following the removal of the entire populations. Though sparsely distributed, a few pollen records reveal ecological responses to Spanish disturbance in the Caribbean.

Study datasets

Pollen data from the Bahamas come from **Blackwood Sinkhole**[164] – a coastal site used to reconstruct hurricane activity. The NAP:AP ratios indicate forest opening at 1520 CE, reverting from an afforestation phase between ~1370 and 1520 CE. The PFG record shows that the 1520 CE NAP:AP shift is driven by an abrupt shift from a *Pinus*-dominant signal (a genera that uses a wind-dispersed pollination strategy) to a *Conocarpus*-dominant signal (a wetland tree that produces pollen far less prolifically than *Pinus*) (Supplement Figure 1). This shift indicates that interpretations of forest cover opening based on NAP:AP ratios are misguided, and rather reflect dispersal biases in the pollen record. Nevertheless, the timing of this shift is consistent with Spanish depopulation of the islands and, at face value, may be interpreted as wetland vegetation recovery following Spanish depopulation. However, the authors of the original study convincingly attribute this change to climate drivers (heightened moisture availability at the site)[164], and

wetland expansion is consistent with a sea level high stand[260]. Consequently, this floristic change is not attributed to Spanish occupation within this study (Fig. 2, Fig. 3). The chronological control of Blackwood Sinkhole is good with 7 dated samples (no reversals) over 2030 years (Supplement Figure 10).

Two pollen records were assessed from the Dominican Republic. The first, **Laguna Biajaca** – a lowland Cibao Valley site[112] – shows low NAP:AP values between the modelled ages of 1473 and 1607 CE[160] (though the noisiness of the dataset means that this change is not captured in the GAM produced for the data (Fig. 3)). PFG data reveal that this increase in forest taxa is driven by an increase in upland *Pinus* (a wind pollinated genus) pollen in the record and co-occurs with reduced fire activity in the landscape[160] (Supplement Figure 1). This suggests forest regrowth, potentially associated with Indigenous population decline, following Spanish occupation of Hispaniola (noting that the ~24-year discrepancy between 1473 CE and disease outbreak in 1497 CE is within the range of dating error for the record)[160]. The PFG data shows that this afforestation is associated with a 5th order cluster (sum of squares =395 – Supplement Figure 1). The record has therefore been classified as showing a minor/uncertain afforestation response to Spanish occupation of Hispaniola. One important aspect of this record is the larger shift in the PFG taxa and NAP:AP ~100 years after Spanish contact, including a large decrease in arboreal taxa relative to general herbs and crop taxa between 1607 and 1663 CE. This indicates heightened land clearance in the lowlands under Spanish control. The chronological control of Laguna Biajaca is poor to moderate with 5 dated points (2 age reversals) over 1020 years (Supplement Figure 11).

The second assessed site in the Dominican Republic, **Arrepentimientos** – an upland record[263] – shows a negative NAP:AP shift after 1500 CE. This change, represented by an increase in upland tree taxa relative to dry herbs, is subtle in the PFG record – i.e. it is not represented by a high order cluster break (sum of squares <200) (Supplement Figure 1) – and is insufficiently resolved to be picked up in the NAP:AP GAM (Fig. 3). It is thus classified as a minor afforestation signal (Fig. 2). The chronological model for Arrepentimientos is moderately well resolved over the past 2,000 years, with 5 dated points over the past 2300 years (1 reversal) (Supplement Figure 12).

Supporting palaeoecological data

NAP:AP data from a second lowland site located in the Cibao Valley, 40km west of Biajaca – ***Los Indios***[264,265] – also show an afforestation signal after Spanish contact. This change is attributed to forest recovery following Spanish disruption of the Indigenous population[136]. Pollen data from a second upland site (Valle de Bao) in the Dominican Republic, however, do not indicate forest recovery following Spanish occupation[266]. This suggests that pre-Spanish land-use and post-Spanish forest recovery may have been spatially constrained. Indeed, a fire record from Laguna Saladilla – a lowland site in the Dominican Republic, as well as Laguna Tortuguera – a lowland lake in Puerto Rico, show no changes in fire activity

following Spanish arrival[267]. Pollen and charcoal records from Lake Miragoane in Haiti indicate increased fire activity, reduced forest cover and increased erosion following Spanish arrival[267], suggesting deforestation rather than afforestation associated with Spanish contact[166,267,268].

South America

Amazon rainforests (including Amazonian Dark Earths)

Study datasets

Two pollen records from the lowland tropical rainforests of the Amazon were included in our analysis – one (Lake Caranga[178]) from the eastern basin, and the other (San Jorge[269]) from peatland forests within the Pastaza-Marañón Foreland Basin (PMFB) in Western Amazonia. Both occur within the vicinity of predicted ADEs[184]

The NAP:AP data from **Lake Carana**[178] – a record that clearly captures a local agricultural signal from ~4500 cal yrs BP – suggests forest opening rather than closing after 1500 CE, though this opening co-occurs with an overall increase in the NAP:AP variability. This shift is captured by the primary cluster boundary of the PFG record (1462 to 1523 CE; sum of squares = 290) marking a general decline in forest taxa relative to dry herbs while wet herbs remain stable (**Supplement Figure 1**). Interestingly, this shift occurs in concert with declining charcoal in the record, indicating decreased site management with fire, highlighting that assuming site abandonment results in forest encroachment in heavily forested regions may be too simplistic in the wet tropics. The chronology of the Lake Carana sequence is moderately constrained over the past 2300 years (n=4 including 1 outlier) (Supplement Figure 13).

The **San Jorge** record[269] derives from a domed peatland – an ecosystem that is particularly sensitive to changes in hydrology and effective moisture, and thus climate change and fluvial processes. There appears to be little information about use of peat forest by pre-Columbian populations, and there is no direct evidence of human land-use within this record. The NAP:AP data from San Jorge show increased relative abundance of forest taxa after 800 CE (Fig. 3) where reduced rates of peat formation permitted the establishment of a closed palm swamp forest[269]. The largest shift in the PFG record occurs between 1371 to 1443 CE, characterized by a large rise in arboreal taxa (palms and the pioneer Cecropia) relative to Pteridophytes (**Supplement Figure 1**). The next youngest sample after this break, modeled at 1515 CE, records zero non-arboreal pollen values. While this is likely associated with the onset of drier conditions in the western Amazon at the end of the MWP[26], which would have encouraged forest encroachment into the peatland, it is not possible to decouple this climate signal from a potential human- signal, and this shift has therefore been classified as showing an unclear/minor afforestation response within both the pre- and post-Spanish period. The 2,400-year San Jorge chronology is well constrained by five ^{14}C and 25 ^{210}Pb dates (Supplement Figure 14).

Supporting palaeoecological data

Previous research examining forest rebound after Spanish occupation in the context of carbon sequestration excludes datasets from the extensive region covered by lowland Amazon tropical forests[32]. This is possibly due to slow interior colonization of the Amazon by Europeans limiting the capacity for rapid disease transmission to inland Indigenous people. However, we do note that 1) the authors of this previous work place disease decline for the Amazon as a whole, at 90%; and 2) that the Amazon rainforest is one of the world largest carbon sinks, making omission of research from this area intriguing[32]. Several palaeoecological records from Amazon rainforest sites in the western Amazon basin (assumed to be less impacted by Indigenous populations)[184], the Amazon-Andean corridor along the far western margin of the basin[193], and the central and east basin, provide additional insight into forest dynamics over the past 2,000 years.

Pollen data from the Lake Huila[193] situated in the Amazon-Andean corridor in Ecuador (likely representing the eastern front of the Inka Empire (1400 to 1532 CE)) shows a signal of significant landscape opening and biomass burning associated with Spanish-Inka conflict between 1588 and 1700 CE, after which there is ~evidence for forest regrowth.

Lake Sauce[181] in the Peruvian (west) Amazon, shows a reduction or abandonment of maize cultivation at 1250 CE, resulting in pre-Spanish afforestation. Four other lake sites around Puerto Maldonado in Peru (Lake Werth, Parker, Vargas and Gentry)[179,270] record low charcoal concentrations after Spanish contact, likely indicating population decline. Pollen data from two of these lake sites show decreased grass concentrations at ~1450 CE (Lake Vargas and Lake Gentry) [131,180,] suggesting pre-Spanish afforestation. However, the other two sites – Lake Werth and Lake Parker – indicate afforestation in the century following Spanish contact. This implies that forest recovery following Spanish conquest was heterogenous across the western Amazon Basin. However, given 1) the proximity of these sites to the Inka Empire (which rose in the early 1400s); and 2) the poorly constrained chronologies for these sites over the critical time period between 1400 and 1600 CE[270], more in-depth analysis is necessary to fully tie down drivers of forest recovery.

Two lake sites from central Amazonia (Santa Maria, and Saracuri)[179] do not appear to show a clear vegetation or fire regime shift in response to Spanish occupation. A third site, Lake Gerai, does however, show a slight increase in arboreal pollen associated with a decrease in maize pollen following Spanish conquest[179]. This demonstrates that, as with the western Amazon, afforestation following depopulation was locally variable and patchy, even within the supposedly more heavily cultivated regions of the Amazon rainforest.

A record from Arapujá Lake in the east Amazon shows forest opening from ~1150 CE (consistent with the onset of drier conditions in the region). A spike in non-arboreal taxa after 1500 CE, suggests, as with the Carana record, deforestation after Iberian contact[26].

The discrepancies between the east, and central/west afforestation signals may relate to different human-climate interactions in the two regions, with the population dynamics in the east influenced by increased aridity after 1150 CE[26].

Llanos de Moxos

Study datasets

Two pollen records from the Llanos de Moxos were included in our analysis. These sites are both located in the ring-ditch region, proximal to the ecotone between the flooded savannas and Amazonian rainforests.

The NAP:AP signal from the **Laguna Granja** record[209] – extracted from a small oxbow lake near the Bella Vista Village archaeological site – shows an afforestation signal between 1353 and 1436 CE. This change corresponds with the largest cluster break in the PFG taxa (sum of squares = 195), marking the partial replacement of dry herb taxa with forest and wet herb taxa (**Supplement Figure 1**). The chronology for Granja is moderately well constrained over the past 2,000 years (n=3) (Supplement Figure 15). This site is thus classified as showing pre-Spanish afforestation (Fig. 2). While this aligns with the original authors' interpretations[208,209], it contradicts with assessments made of a post-Spanish afforestation signal in a recent review[32].

Laguna Orícore – also proximal to the Bella Vista Village archaeological site – is a much larger site than Laguna Granja, and thus is expected to better capture regional forest change[209]. NAP:AP (Fig. 3) and PFG (**Supplement Figure 1**) data from this site indicate that forest cover has been reasonably stable since ~200 CE when the region became more humid. This indicates that climate has been the primary influence on vegetation structure at this site[209]. The chronology for Oricore shows a linear age-depth trend over the past 5680 yrs BP, though the youngest dated sample is 1930 yr BP at 17.5cm (Supplement Figure 16). This means that this record is not ideal for tying down change points over the past 2,000 years. However, given the stability of forest cover at this site, this not considered especially problematic for our interpretation. We have classified this site as showing no afforestation signal within either the pre-Spanish (1000 to 1500 CE) or Spanish (1500 to 1600 CE) period (Fig. 2).

Supporting palaeoecological data

The Llanos de Moxos is one of the better-studied regions in South America from a palynological perspective. Records from nine additional sites mirror the same responses from those observed in the Oricore and Granja records – i.e. limited afforestation over the past 2,000 years (San José[210], Lago

Rogaguado[211], Chalalán[212], Santa Rosa[212], Acre soil profiles[190] and Madre de Dios soil profiles[192]), or afforestation preceding Spanish contact (La Luna[201], El Cerrito[205] and Laguna Frontera[205]). Overall, this provides relatively compelling evidence that Spanish arrival and depopulation did not trigger significant forest regrowth within the Llanos de Moxos. The Spanish did, in places, alter the landscape in the years after arrival through the introduction of novel weeds and crops (e.g. oats (*Avena*) and sorrel (*Rumex acetosella*)[211]) and potentially intensifying cacao cultivation on forest mounds[206]. However, the use of "traditional" raised field agriculture persisted well into Spanish rule[205].

Los Llanos

Study datasets

NAP:AP and PFG data for **Laguna Carimagua**[271] indicate slight forest closure between 1000 and 1200 CE succeeded by opening, and no clear forest response to Spanish-caused depopulation between 1500 and 1600 CE (Fig. 3; **Supplement Figure 1**). This record has thus been classified as showing a pre-Iberian afforestation signal (Fig. 2). The chronological model for Laguna Carimagua is very poor over the past 2,000 years. There are 6 samples taken between 0 and 1,100 yrs BP, including 3 reversals that were excluded from the age-depth model (Supplement Figure 17).

Laguna Mozambique[220] NAP:AP and PFG data show afforestation from 1350 CE, which occurs alongside a replacement of a potentially anthropogenic Mauritia swamp forest (included as a tropical evergreen forest tree in the PFG groupings) with lowland tropical forest taxa (**Supplement Figure 1**). A second, larger, temporary afforestation response between 1532 and 1595 CE provides compelling evidence for depopulation as a driver of increased forest cover. This record has been classified as showing both a pre- and post- Iberian afforestation signal (Fig. 2). The chronological model for Laguna Mozambique over the past 2,000 years is moderately good, with three samples between the core top and 240 cm (2187 yr BP) (Supplement Figure 18).

Supporting palaeoecological data

In addition to the sites assessed in this study, two other records from southwestern Los Llanos lakes (Chenevo[220] and Las Margaritas[272]), and one record from the north Los Llanos (Los Briceño[217]), provide insight into vegetation dynamics associated with Spanish conquest. There is evidence for a subtle afforestation signal in pollen data from Lagunas Chenevo after Spanish occupation[220] while pollen data from Las Margaritas[272] (which terminates at 300 CE) do not show any afforestation response after Spanish conquest. Los Briceño, an oxbow lake proximal to the El Cedral archaeological region, shows a dramatic increase in grass pollen and charcoal at the expense of forest tree taxa following land-use intensification and clearance by fire at ~50 CE. This change persists over the following 2 millennia, including in the centuries following Spanish conquest. This likely reflects a persistent ecological state shift from forest to savanna that

could not be reversed even if human disturbance were removed or decreased following Spanish conquest. This record highlights issues associated with assuming linear ecological responses to perturbation or perturbation removal, particularly within grassland biomes.

Cerrado

Study datasets

The NAP:AP and PFG data from the **Lagoa Campestre de Salitre** peat mire bog in the central-southeast Cerrado[273] indicate progressive Pre-Iberian afforestation from 1272 to 1380 CE driven by an increase in tropical evergreen taxa (Fig. 2; **Supplement Figure 1**). This shift occurs just prior to the post-MWP transition from drier to more humid conditions in the region[26]. The lowest point in the NAP:AP curve (and peak in tropical evergreen taxa) coincides with the "Great Dying" period, and this record has thus been classified as showing "minor afforestation" in response to Spanish contact (Fig. 2). The chronological model produced for this record over the past 2,000 years is poor, with the youngest sample from this sequence (18cm depth) returning an age of 3060 ^{14}C BP (3219 to 3368 cal yrs BP at 2σ) (Supplement Figure 19).

Supporting palaeoecological data

Two sites within the southeast Cerrado region, Lagoa Olhos D'água[20] and Lagoa dos Mares[20], show an increase in arboreal taxa between >1350 and 1550 CE, reflecting a similar signal to that seen in the Lagoa Campestre de Salitre record (i.e. pre-Iberian and early Portuguese period afforestation). Again, this afforestation may be driven by increased humidity in the region from ~1350 CE.

Atlantic Forests

Study datasets

NAP:AP and clustered PFG data from **Lago do Pires**[274] show an increase in tropical forest taxa between 969 and 1107 CE (Fig. 3; **Supplement Figure 1**), coincident with the onset of more humid conditions at the start of the MWP[26]. There is no clear evidence for an afforestation signal associated with Iberian caused depopulation. The largest change in the pollen record is the decline in forest taxa during the industrial period (1816 to 1916 CE), associated with intensified clearance of the landscape[274]. This record has been classified as showing a pre-Iberian afforestation signal (Fig. 2). Aside from a similar signal of deforestation between 1858 to 1994 CE, the pollen data from **Morro de Itapeva**[275] show forest stability over the past 2,000 years (Fig. 3; **Supplement Figure 1**). The chronological models for both Atlantic Forest sites included in this analysis are poor over the past 2,000 years (n=1) (Supplement Figure 20; Supplement Figure 21).

Supporting palaeoecological data

Serra Campos Gerais[276], a bog within the lowland Atlantic rainforests, shows expansion of Araucaria forest under wetter conditions from ~500 CE. There is no clear forest cover or fire-regime change in response to

Iberian contact despite the presence of maize in the local environment prior to Spanish contact. As with the other Atlantic forest records, there is heightened anthropogenic disturbance (selective deforestation and cropping) in recent centuries. The age-depth models from a series of other pollen records from the region[277] are insufficiently resolved to determine vegetation response to Iberian colonization. However, all tend to show forest stability until recent centuries, which are associated with heightened fire and deforestation[277].

Pacific Forests

Study datasets

PFG data and the NAP:AP GAM from **Laguna Piusbi**[234] show forest stability over 2,000 years (**Supplement Figure 1**; Fig. 3). The largest shift in PFG data occurs between 1349 and 1434 CE and is associated with a very subtle increase in dryland vs. wetland trees, potentially linked to tectonic uplift. This record has been classified as showing no forest response within the pre-Spanish (1000 to 1500 CE) or post-Spanish (1500 to 1600 CE) period (Fig. 2).

NAP:AP data from **Laguna El Caimito**[278] indicate increased forest cover after ~1390 CE, and this shift has been classified as a pre-Spanish afforestation signal. However, clustering of the PFG pollen data from this record show that this change is accompanied by a decrease in wetland trees between 1388 and 1403 CE (**Supplement Figure 1**). This change mirrors the nature and timing of vegetation change seen in the Piusbi record and indicates that afforestation may be driven by regional tectonic uplift.

The chronological models for Laguna Piusbi and Laguna El Caimito are moderately well constrained over the past 2,000 years (n=3; Supplement Figure 22; Supplement Figure 23).

Supporting palaeoecological data

Other records from the Pacific Forests[279,280] show a similar patterns of forest stability in response Spanish occupation. Sites within the northern part of the Chocó region do, however, indicate an increase in disturbance proxies after Spanish occupation[280].

Andes

<2,000m ASL: Tropical Dry Forest Valleys (Cauca and Patia)

Four sites – Quilichao swampland[281], La Teta[281], Pantano de Genagra swampland[282] and Piagua[283] occur within the Cauca River Dry Forest Valley or within lower montane forest above the Cauca dry forests. One site – Patia Swamp[284] – occurs within the Patía Valley dry forest ecoregion.

The record (**Patía-2**) analysed from Patia Swamp[284] represents the lowest elevation site (750m ASL) assessed from the Andes. The NAP:AP data show a stepwise increase in forest elements from ~1000 to 1201 CE, and 1343 to 1437 CE (Fig. 2). Noting that the chronology for this record is poorly constrained over the past 2,000 years (n=1; Supplement Figure 24) the earlier of these shifts may reflect an afforestation response

to social reorganization after 900 CE (coincident with onset of more arid conditions[25] which should encourage expansion of a more open rather than closed environment). The afforestation at 1343 to 1437 CE is consistent with the onset of wetter conditions during the LIA[25]. The PFG records shows an increase in lowland tropical forest (including secondary and dry forest types) in the record after ~1500 CE. Though this change is not captured as a primary or secondary shift in cluster analysis of the data (**Supplement Figure 1**), it hints at the recovery of some forest elements in the lowlands coincident with Columbian depopulation. This record is thus assessed to show a pre-Spanish and minor Spanish afforestation signal (Fig. 2).

NAP:AP data from **Quilichao swampland**[281] show afforestation between ~1350 and 1550 CE after which forest cover stabilizes until the post Industrial period (~1875 CE). Afforestation occurs after sustained, high NAP:AP values during an arid MWP, suggesting climatic drivers of overarching forest change. However, the low NAP:AP values between 1524 and 1544 CE correspond to a rise in tropical/temperate evergreen species in the PFG data – defined by the primary cluster break (sum of squares = 690) (**Supplement Figure 1**). This record is thus interpreted to show a pre-Spanish and minor Spanish afforestation signal (Fig. 2). The chronology for Quilichao-1 is well constrained over the past 2,000 years (n=5) (Supplement Figure 25).

NAP:AP and PFG data from **La Teta**[281] – a second swamp site proximal to Quilichao, indicate afforestation from ~1200 CE (Fig. 3), and has been classified as showing a pre-Iberian afforestation signal (Fig. 2) There is no increase in forest cover following Spanish contact. Rather, an increase in pteridophytes between 1573 and 1664 CE, may reflect intensified disturbance (**Supplement Figure 1**). The chronological model for the past 2,000 years is moderately well constrained (n=3) (Supplement Figure 26).

Like La Teta, the pollen data from the **Pantano de Genagra swampland**[282] – a site located above the Cauca dry forests within the Northwestern Andean montane forests – show afforestation from ~1200 CE (1134 to 1242 CE) associated with the replacement of generalist herbs with wetland herbs and forest taxa (Fig. 3; **Supplement Figure 1**). There is no evidence for afforestation in response to Spanish occupation, and the GAM produced for the site indicates gradual, linear afforestation over the past 2,000 years (Fig. 3). This site has thus been classified as showing a pre-Spanish afforestation signal. The chronological model for the Pantano de Genagra record over the past 2,000 years is poor (n=1) (Supplement Figure 27)

The NAP:AP record from ***Piagua swampland***[283] – a site proximal to Genagra – shows a marked decrease in forest elements between ~1220 and 1360 CE, after which arboreal taxa increase. Particularly low NAP:AP values occur from ~1430 to 1720 CE (Fig. 3). The PFG data for this site indicate that the shift from high to low NAP:AP is associated with a slight (~4%) increase in dry forest trees/shrubs and a large (~30 to 50%) increase in wetland herbs at the expense of dry herbs (**Supplement Figure 1**). This shift may be attributable to the strengthened South American Monsoon over the Andes during the LIA. However, the record has been

classified as showing pre-Spanish and Spanish (minor) afforestation (Fig. 2). The Piagua chronological model is moderately well constrained over the past 1,900 years (n=3) (Supplement Figure 28).

2,000 to 3,000m ASL: Eastern Cordillera real montane forests

Laguna De La Cocha[285] and Laguna Cocha Caranga[286] occur within the Eastern Cordillera montane forest within southern Columbia and northern Peru.

The NAP:AP data from **Laguna de la Cocha** indicate high amplitude fluctuations in forest cover between 902 and 1546 CE, possibly associated with human disturbance of the site. These shifts are not captured in the GAM and are thus not interpreted as pre-Spanish afforestation (or deforestation) signals. After 1546 CE, the NAP:AP data show distinct afforestation, potentially linked to forest regrowth after Spanish-caused depopulation (Fig. 3). However, the timing of this shift coincides with a strengthened SASM during the LIA[25], and PFG data reveal reorganization of very local taxa (including a sharp decline in wet herbs and large increase in pteridophytes (**Supplement Figure 1**)), suggesting that drivers of this change may relate to climatically driven shifts in site hydrology. Consequently, the vegetation change at 1546 CE is interpreted as a minor/uncertain afforestation signal associated with Spanish occupation (Fig. 2). The chronology for Laguna De La Cocha is reasonably well constrained over the past 2,000 years (n=3) (Supplement Figure 29))

Laguna Cocha Caranga[286] occurs with the Ecuadorian Andes, south of the other Andean sites included in this analysis, and at the boundary of pre-Spanish Inka territory (Fig. 2). NAP:AP data from this site show gradual afforestation from 50 BCE to 1200-1400 CE, followed by a very slight deforestation signal between 1400 CE and the youngest sample (Fig. 3). PFG data reveal that the largest biome-scale change in the record occurs at 860 and 920 CE, associated with an increase in dryland (including forest) taxa, and a decrease in charcoal and disturbance indicators at the site (**Supplement Figure 1**). This likely reflects a decline in human activity, or perhaps deliberate reforestation of the site[287]. This record has been interpreted as showing a pre-Spanish afforestation signal (Fig. 2). The chronology of Laguna Cocha Caranga is poor over the past 2,000 years (n=1) (Supplement Figure 30).

Upland Andean sites (> 3,000m ASL)

Four records were assessed from the upper Andes – three within upper montane forest ecoregions (Llano Grande Mire[288], Paramo de Piedras Blancas[289] and Páramo de Agua Blanca[290,291]), and one in the Andean páramo, just above the forest line (Laguna de los Bobos[292]).

Paramo de Piedras Blancas[289] is the most northerly site assessed from the Andes and is located in the Venezuelan Andes Montane Forests. NAP:AP data from this site show a gradual deforestation signal from the start of the record (~610 CE) to 1450 CE (with a large peak at 1370 CE), followed by progressive

afforestation (Fig. 3). Assessment of the PFG data, however, reveal very low forest pollen percentages throughout (AP<5% of count), and an exceptionally high number of indeterminate pollen grains, particularly after 1450 CE, coinciding with decreased NAP:AP values (**Supplement Figure 1**). The unclear parent of the unknown pollen type/s, which comprise up to ~70% of the pollen count (1570 CE) means that we have classified this record as showing no forest response (Fig. 2). The chronology of Paramo de Piedras Blancas is poorly constrained over the past 2,000 years (n=2) (Supplement Figure 31).

NAP:AP and PFG data from **Llano Grande Mire**[288] show a stepwise increase in NAP:AP at 547 to 639 CE (associated with a hydrologically driven increase in dryland taxa), and at 1310 to 1700 CE (Fig. 3; Supplement Figure 1). This latter peak is attributed to the deforestation of evergreen tree taxa (*Quercus, Podocarpus* and *Weinmannia*). NAP:AP ratios remain high over the Spanish occupation period. This record is assessed as showing no afforestation response between 1000 and 1600 CE (Fig. 2). The chronology of from Llano Grande Mire is poorly constrained over the past 2,000 years (n=1) (Supplement Figure 32).

The NAP:AP GAM from **Páramo de Agua Blanca**[213,214], within the southern part of the pre-Spanish Muisca confederation[290,291] (Fig. 2a, shows a gradual, linear deforestation trend over the past 2,000 years (Fig. 3). The PFG data indicate that this change is associated with an increase in generalist herbs at the expense of pteridophytes and montane forest taxa between 1542 and 1630 CE (**Supplement Figure 1**). The timing of this shift coincides with removal of crop pollen from the record, suggesting, if anything, a deforestation signal associated with Spanish occupation. The chronological control of this record over the last 2,000 years is poor (n=1) (Supplement Figure 33). Because of this uncertainty, and the subtlety of post-Spanish deforestation, this record has been classified as showing no afforestation response between 1000 and 1600 CE, and no deforestation response to Spanish occupation (Fig. 3).

NAP:AP and PFG data from ***Laguna de los Bobos***[292], also within the bounds of the pre-Spanish Muisca confederation, show a post-MWP increase in forest tree taxa at ~1300 CE (Fig. 3). A slight dip in NAP:AP at ~1520 CE may reflect minor forest recovery and/or afforestation in response to increased SASM strength during the LIA. This record has been classified as showing both a pre-Spanish (1000 to 1500) and minor/uncertain post-Spanish (1500 to 1600 CE) afforestation response (Fig. 2). The chronology of Laguna de los Bobos is moderately well constrained (n =3) (Supplement Figure 34).

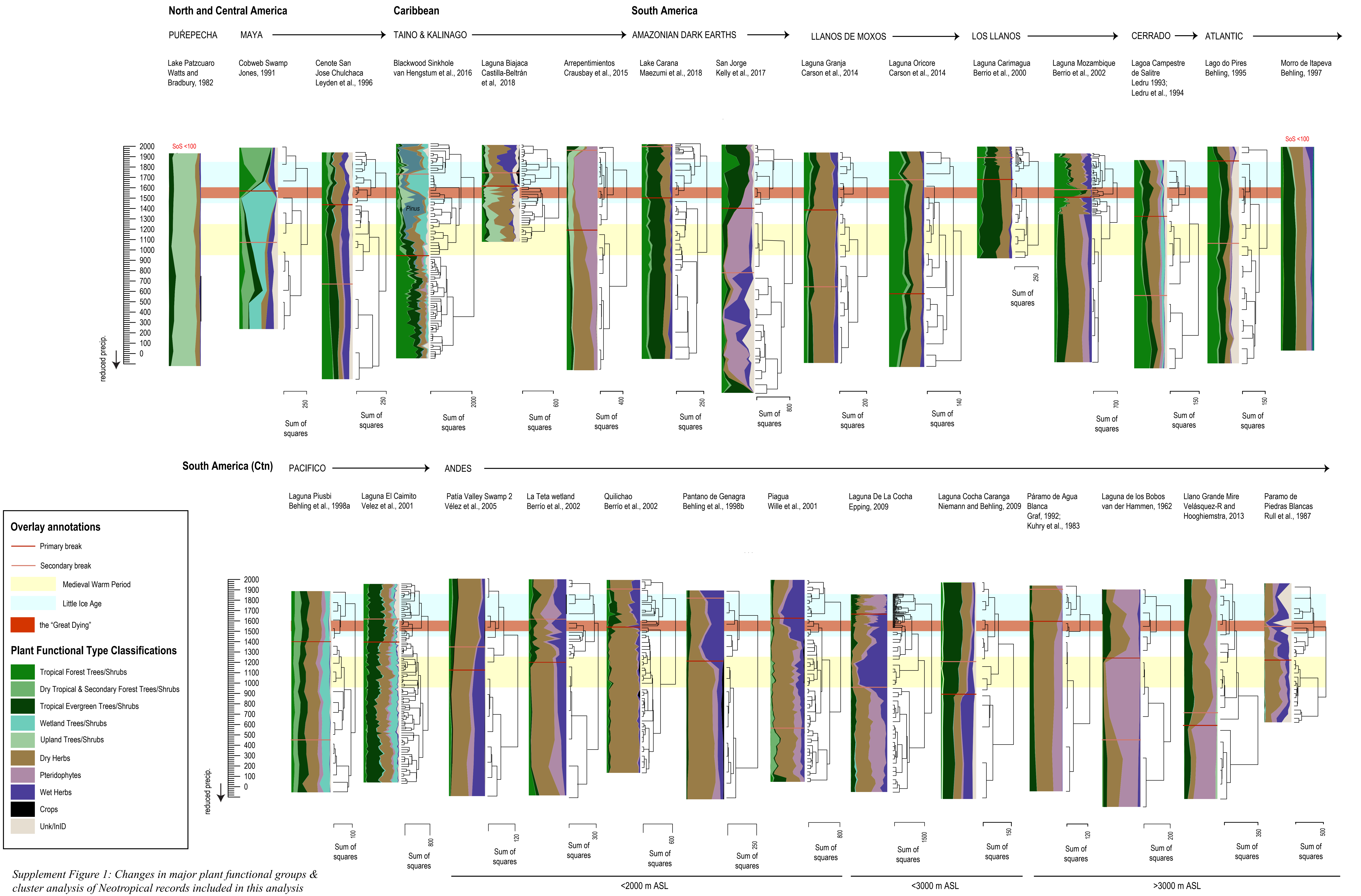

Supplement Figure 1: Changes in major plant functional groups & cluster analysis of Neotropical records included in this analysis

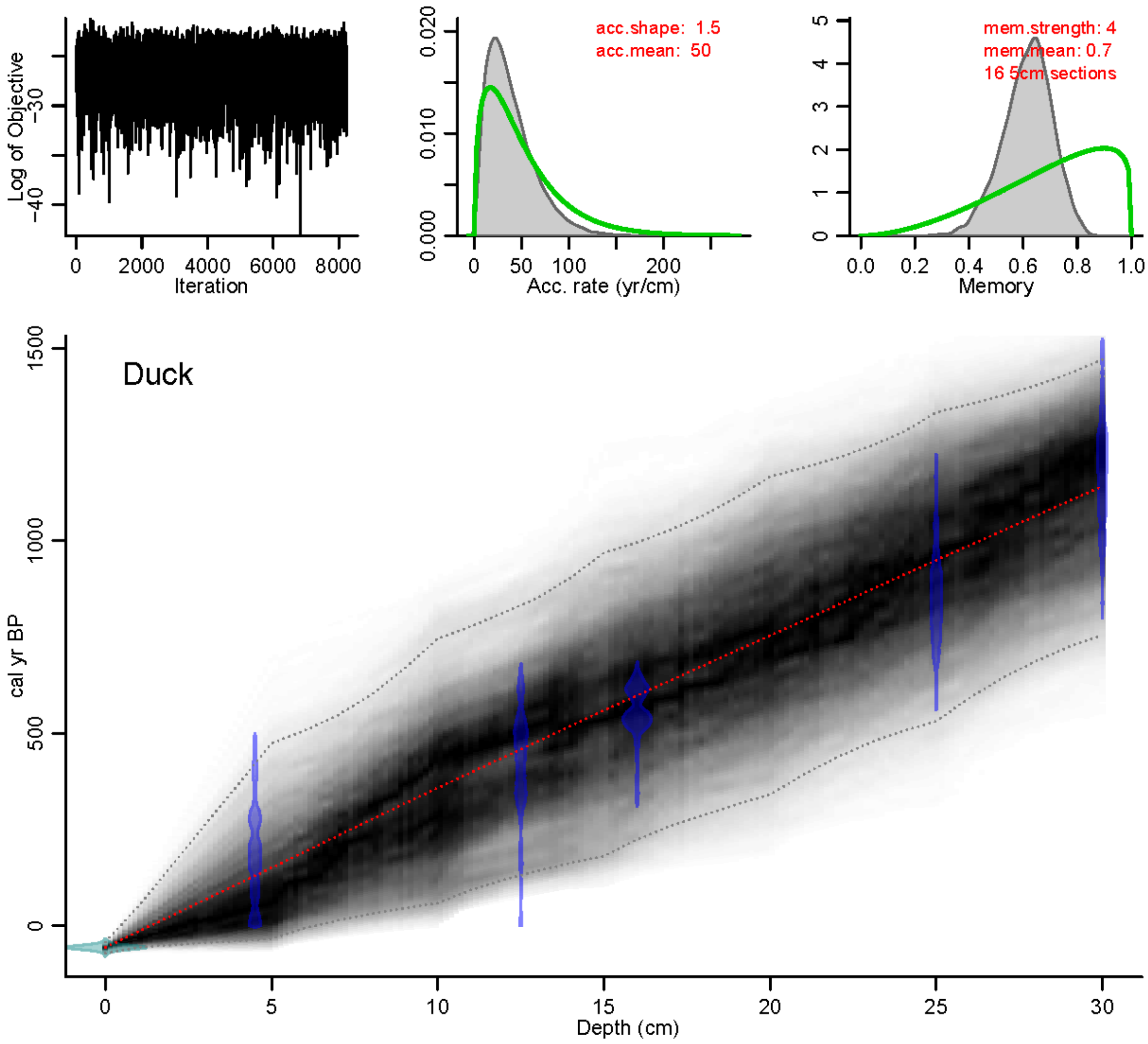

Supplement Figure 2: updated chronological model for Duck Pond.

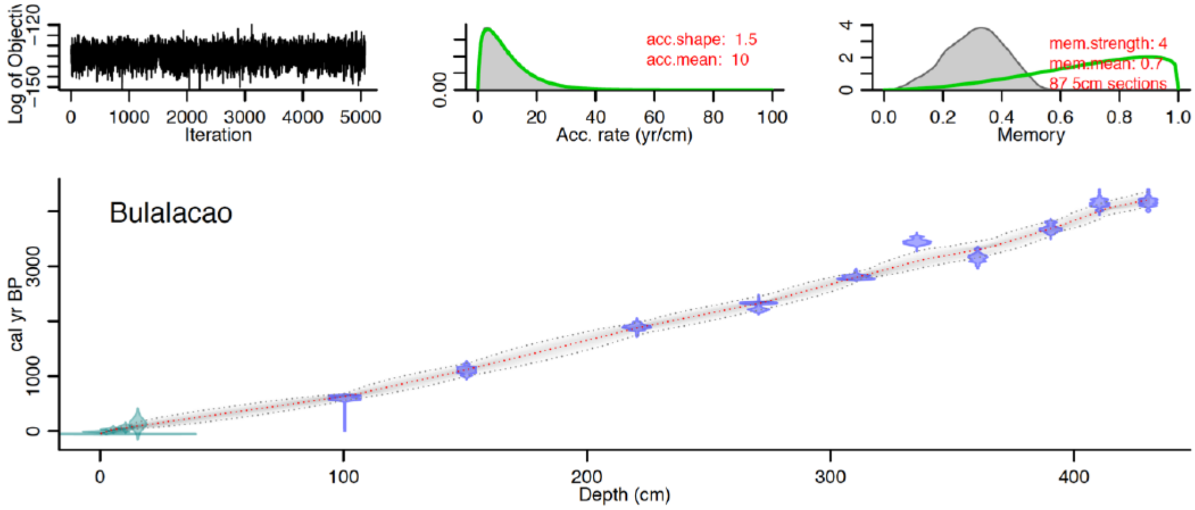

Supplement Figure 3: Lake Bulalacao chronological model (unpublished).

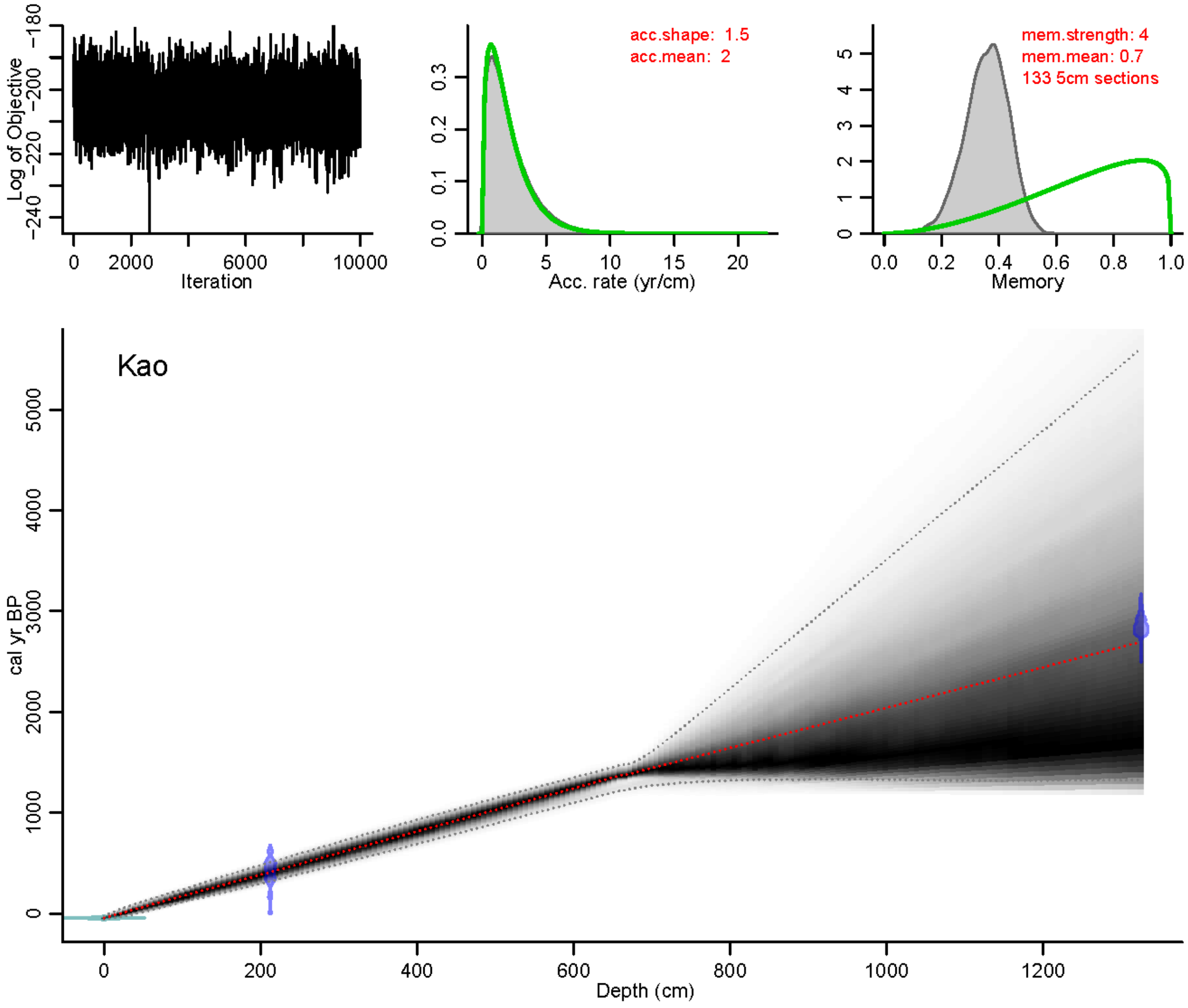

Supplement Figure 4: updated chronological model for Kao River Plain.

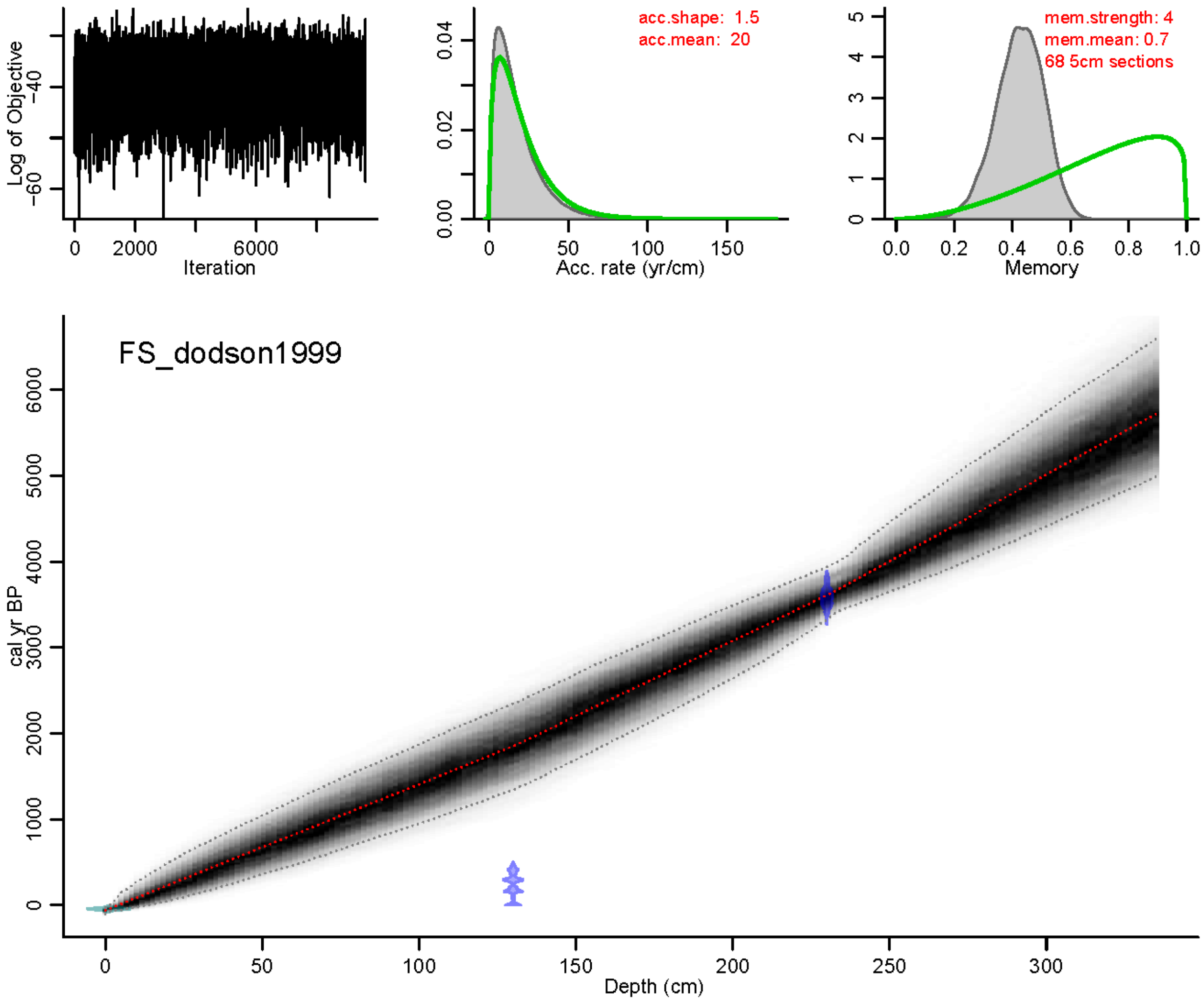

Supplement Figure 5: updated chronology for Fool Wetland (Dodson, 1999).

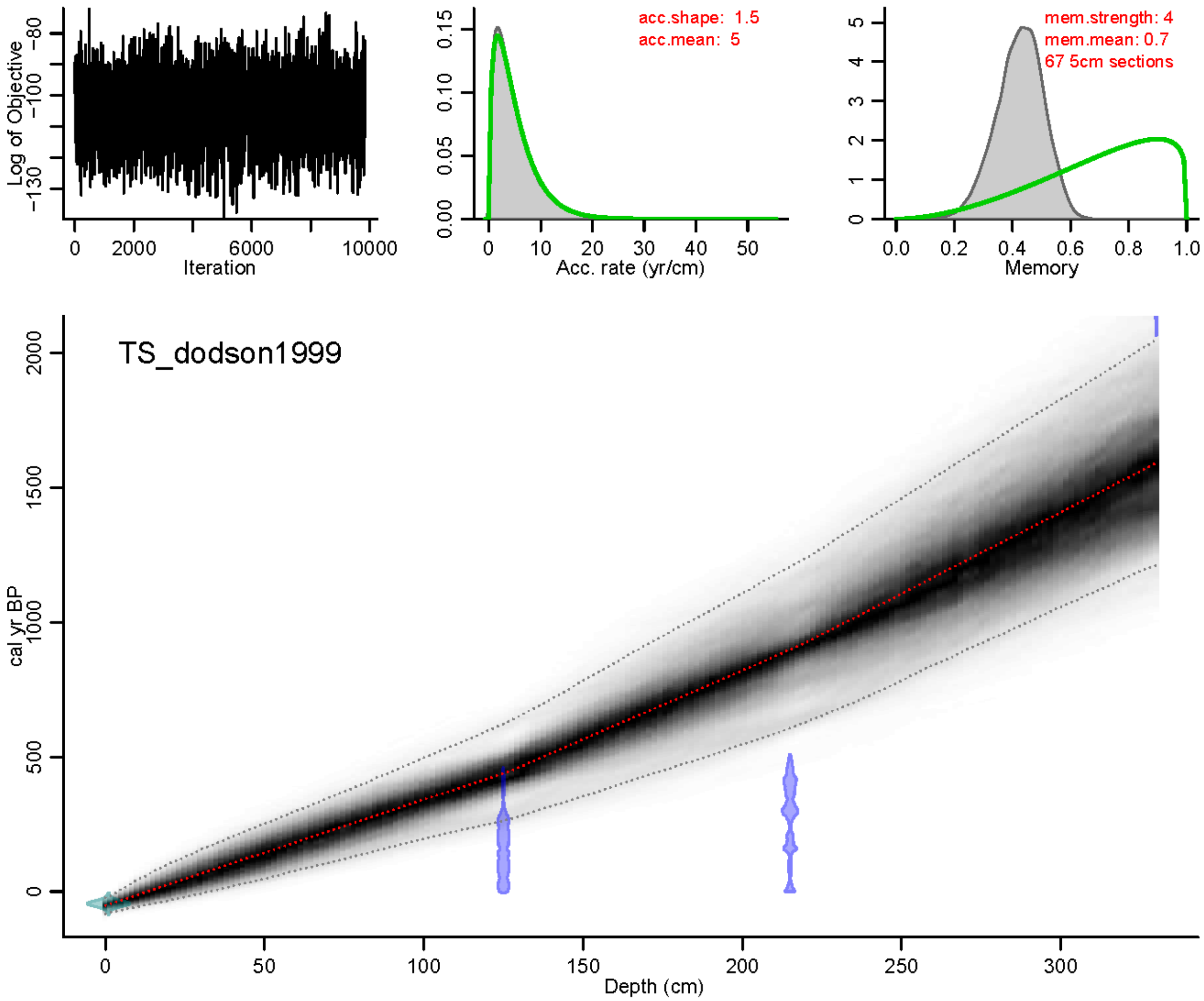

Supplement Figure 6: updated chronology for Thool Wetland (Dodson, 1999).

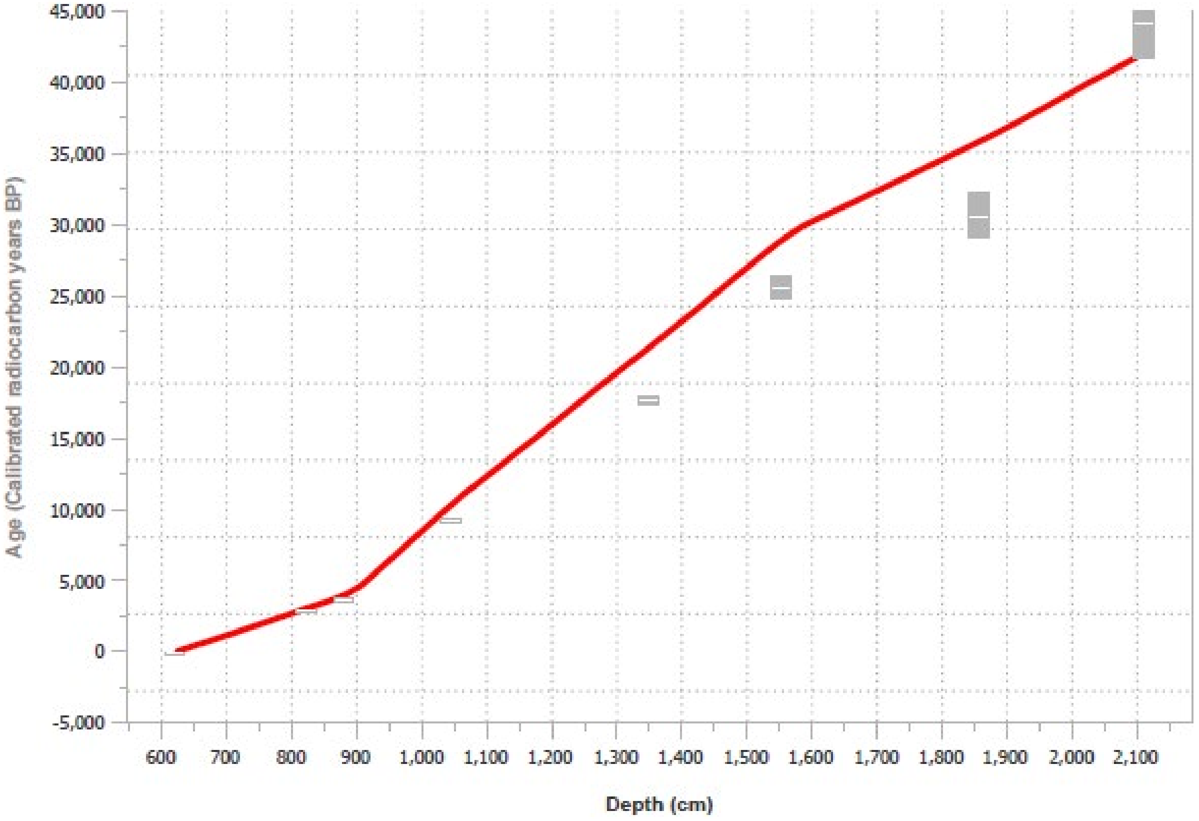

Supplement Figure 7: Lake Patzcuaro chronological model (LADP). Graph extracted from Neotoma database 07/2020[244]

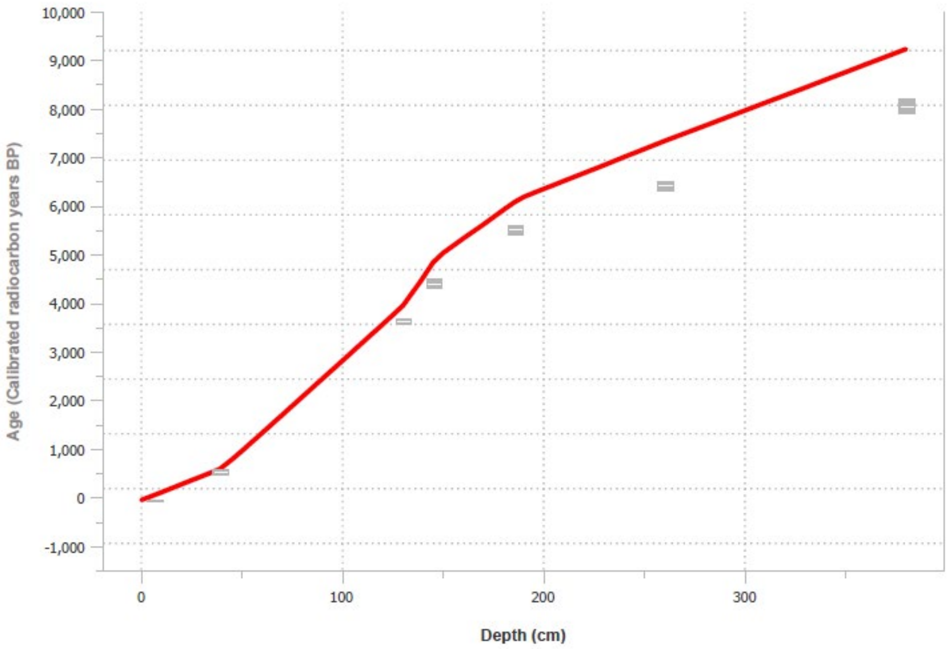

Supplement Figure 8: Cobweb Swamp chronological model (Neotoma1). Graph extracted from Neotoma database 07/2020[244]

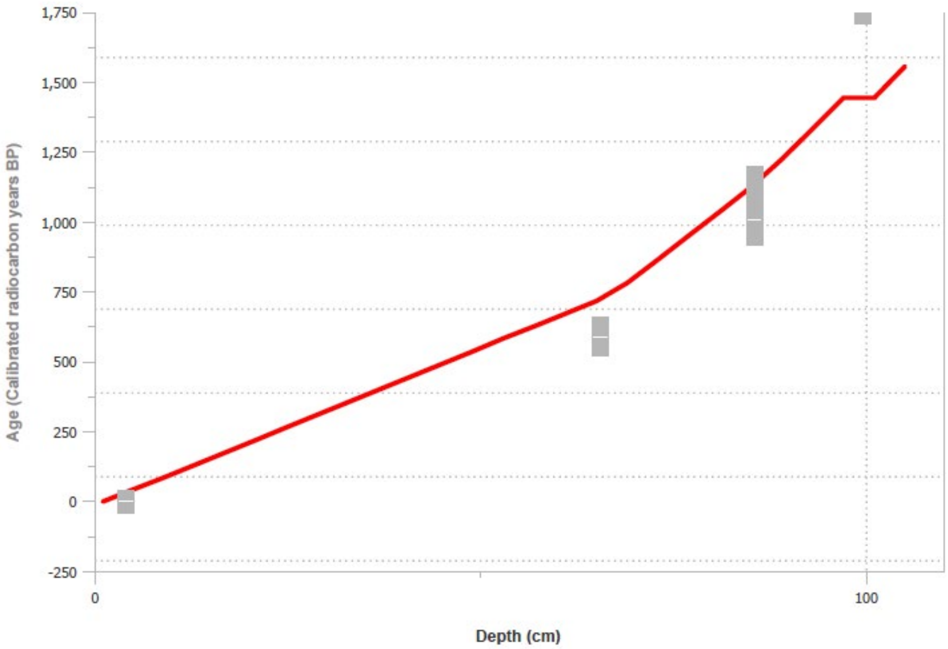

Supplement Figure 9: Cenote San Jose Chulchaca chronological model (LAPD1). Graph extracted from Neotoma database 07/2020[244]

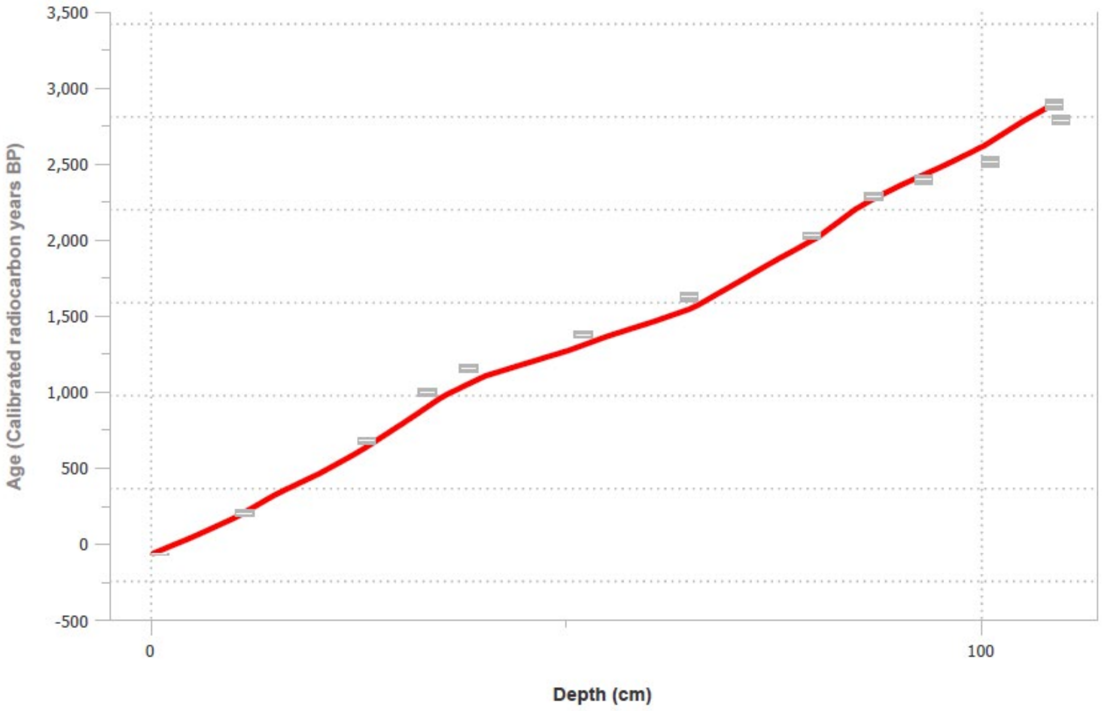

Supplement Figure 10: Blackwood Sinkhole chronological model (van Hengstum et al. 2016). Graph extracted from Neotoma database 07/2020[244]

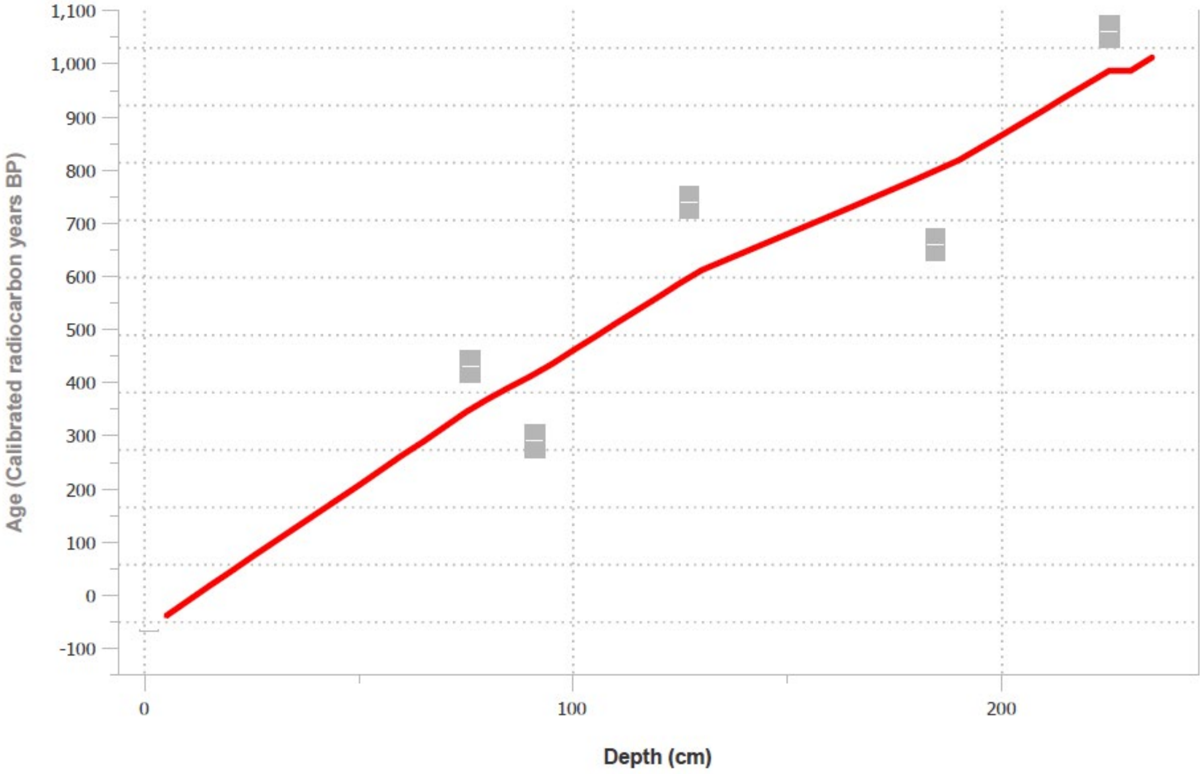

Supplement Figure 11 Laguna Biajaca chronological model (LADP-1). Graph extracted from Neotoma database 07/2020[244]

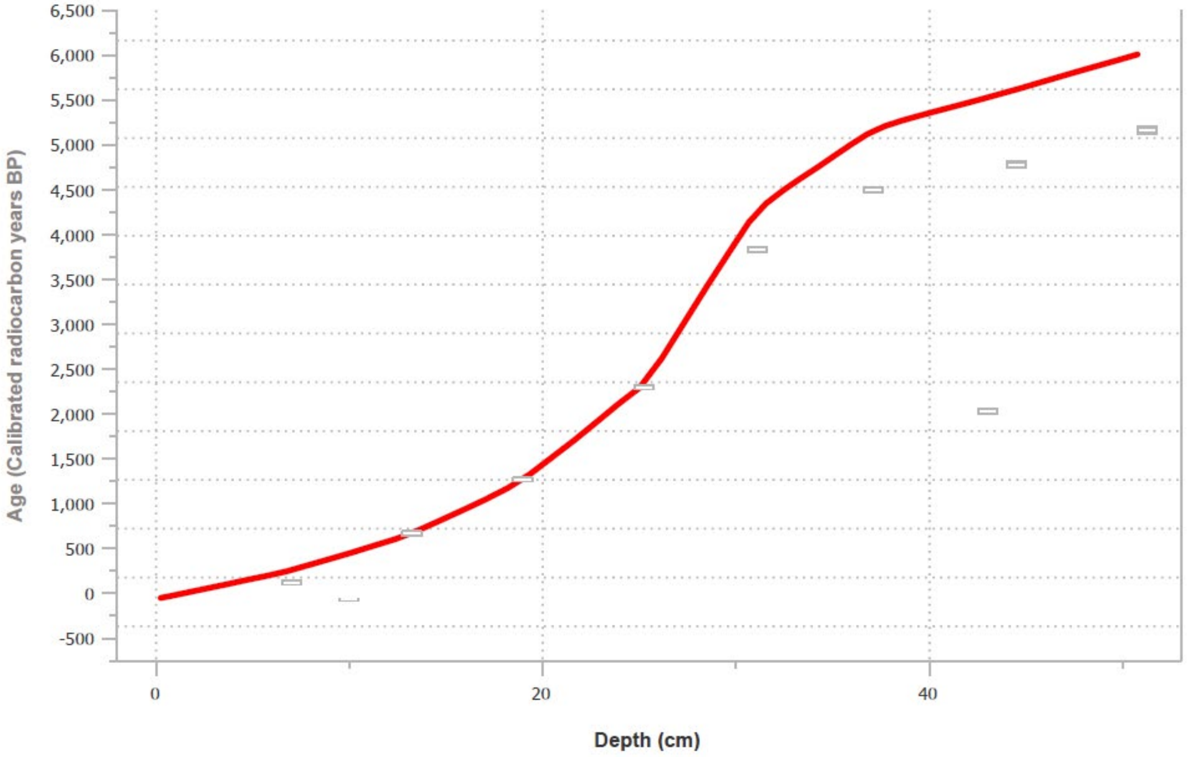

Supplement Figure 12: Arrepentimientos chronological model (Neotoma 1) Graph extracted from Neotoma database 07/2020[244]

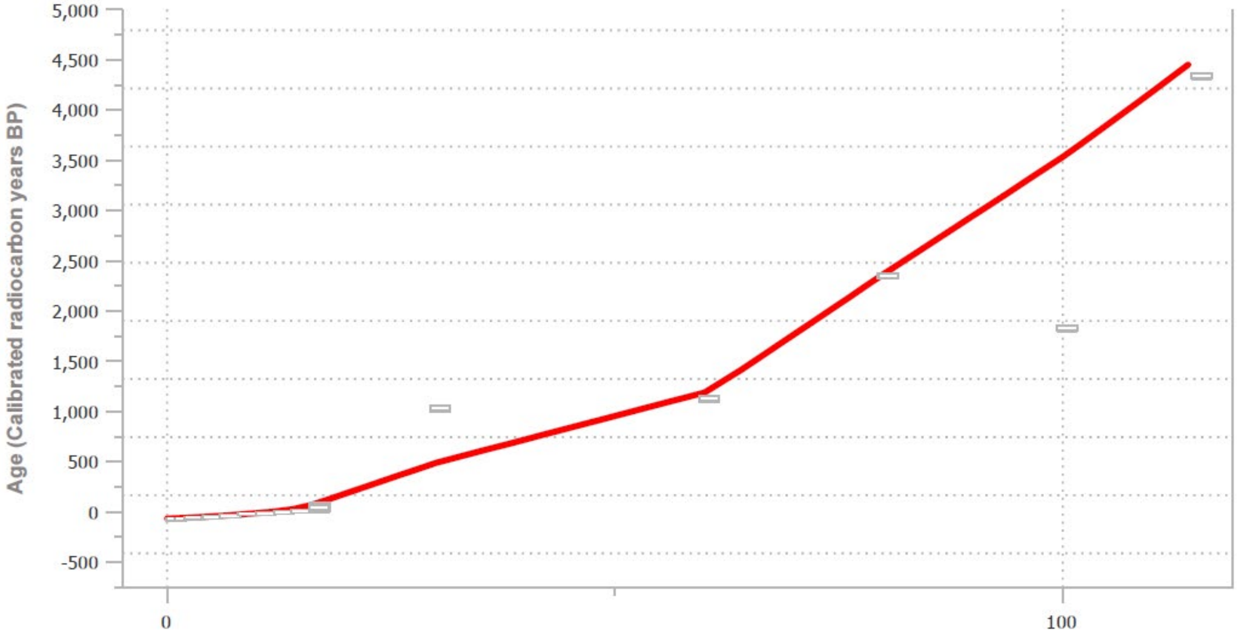

Supplement Figure 13: Lake Carana chronological model (Maezumi et al 2018). Graph extracted from Neotoma database 07/2020[244]

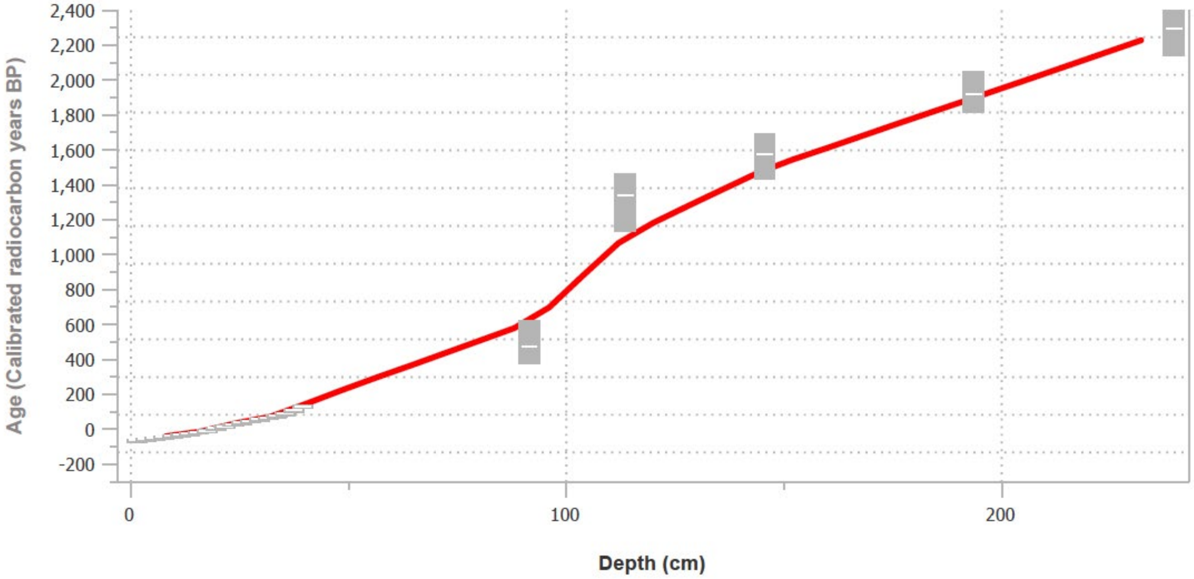

Supplement Figure 14: San Jorge chronological model (Neotoma 1). Graph extracted from Neotoma database 07/2020[244]

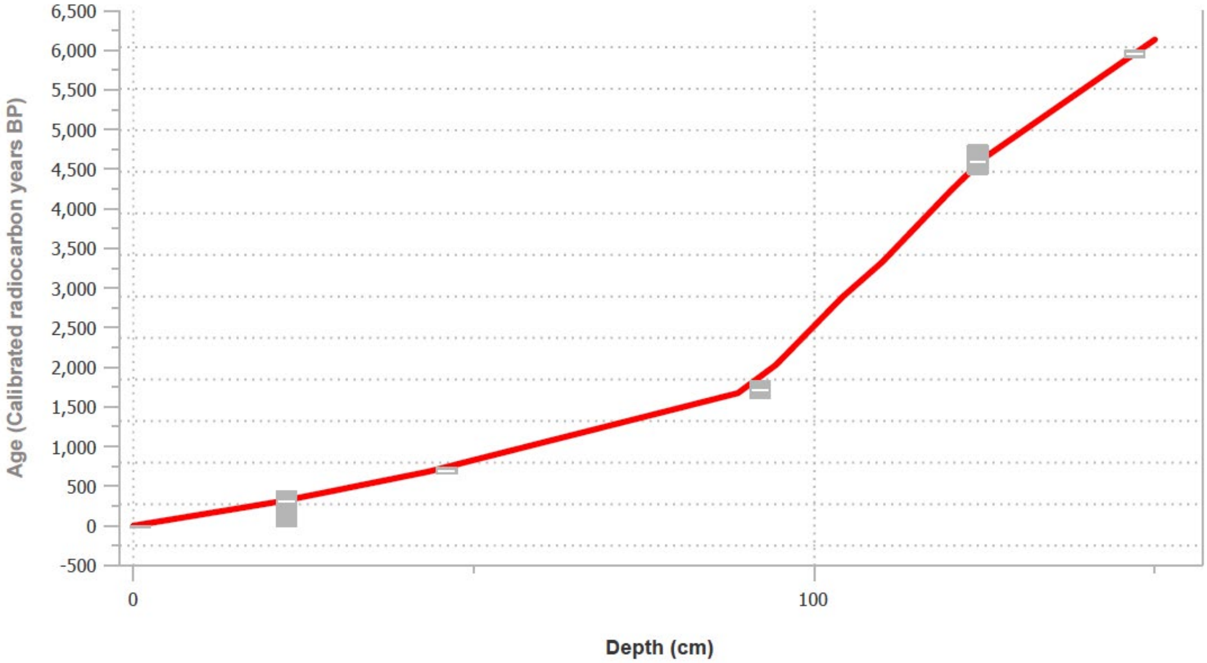

Supplement Figure 15: Laguna Granja chronological model (Carson et al. 2014). Graph extracted from Neotoma database 07/2020[244]

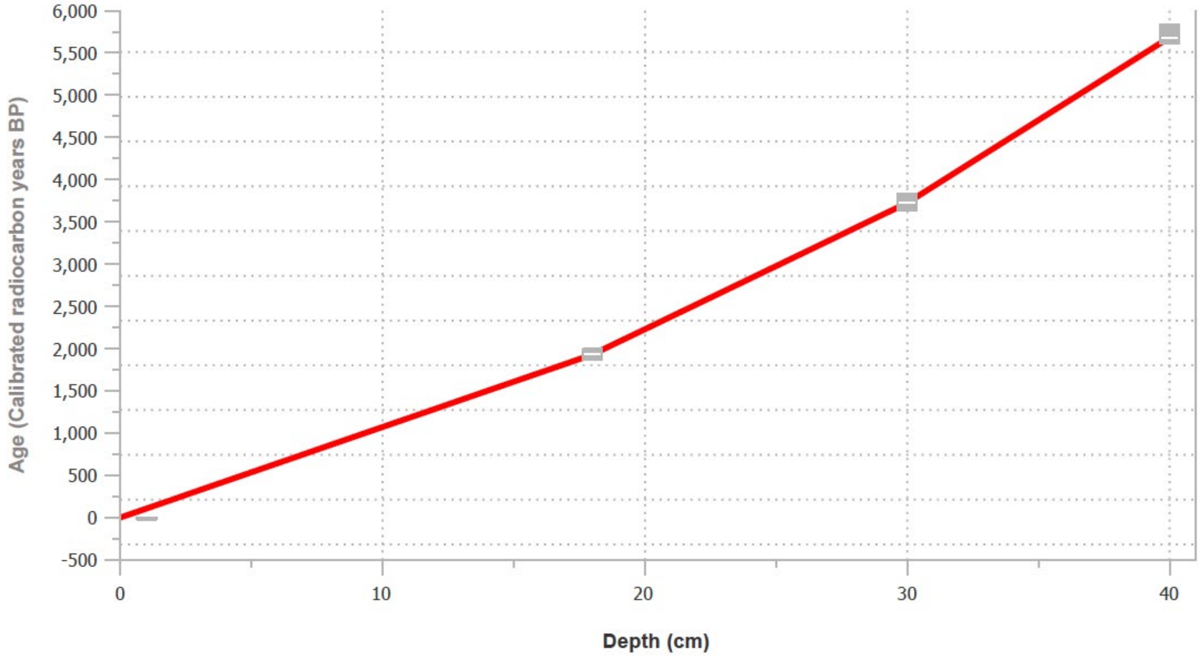

Supplement Figure 16: Oricore chronological model (Carson et al. 2014). Graph extracted from Neotoma database 07/2020[244]

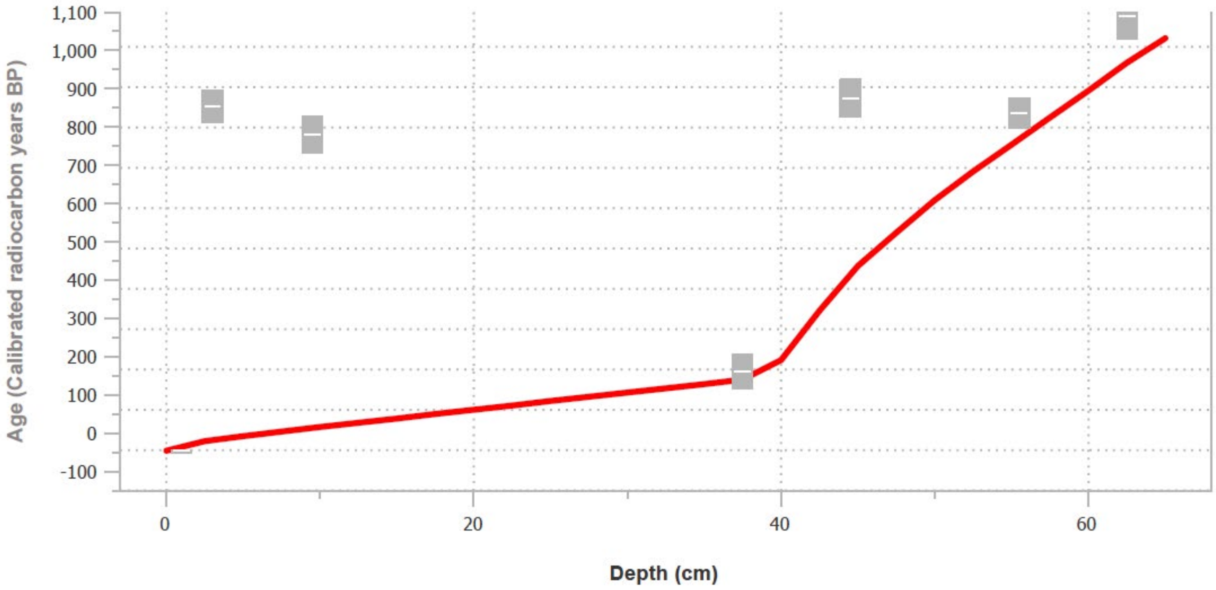

Supplement Figure 17: Laguna Carimagua chronological model (DANS1). Graph extracted from Neotoma database 07/2020[244]

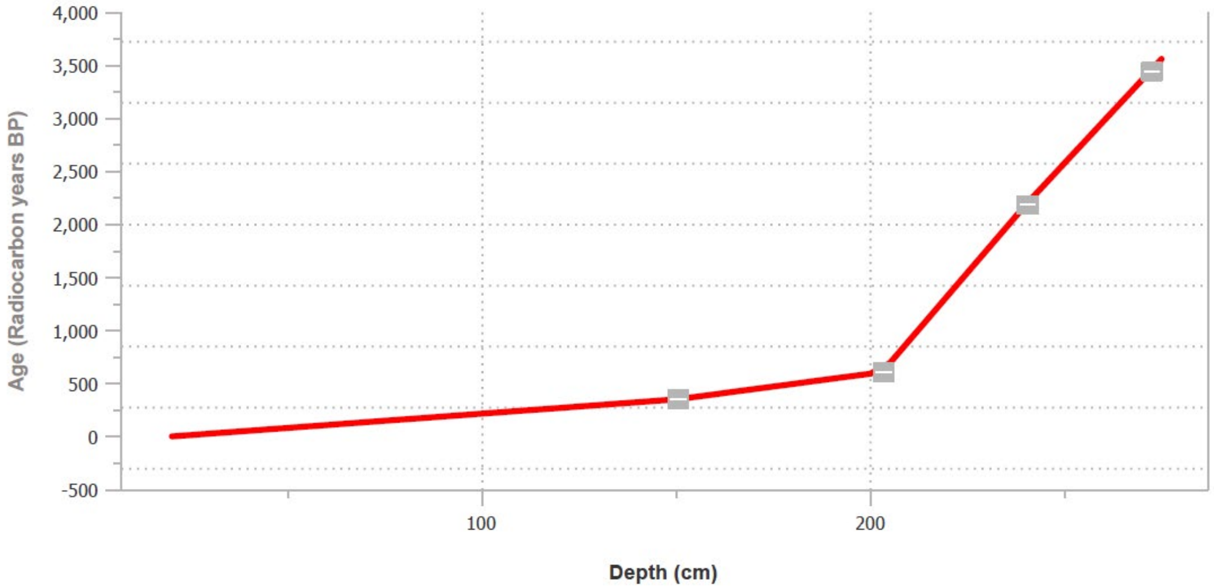

Supplement Figure 18: Laguna Mozambique chronological model (DANS1). Graph extracted from Neotoma database 07/2020[244]

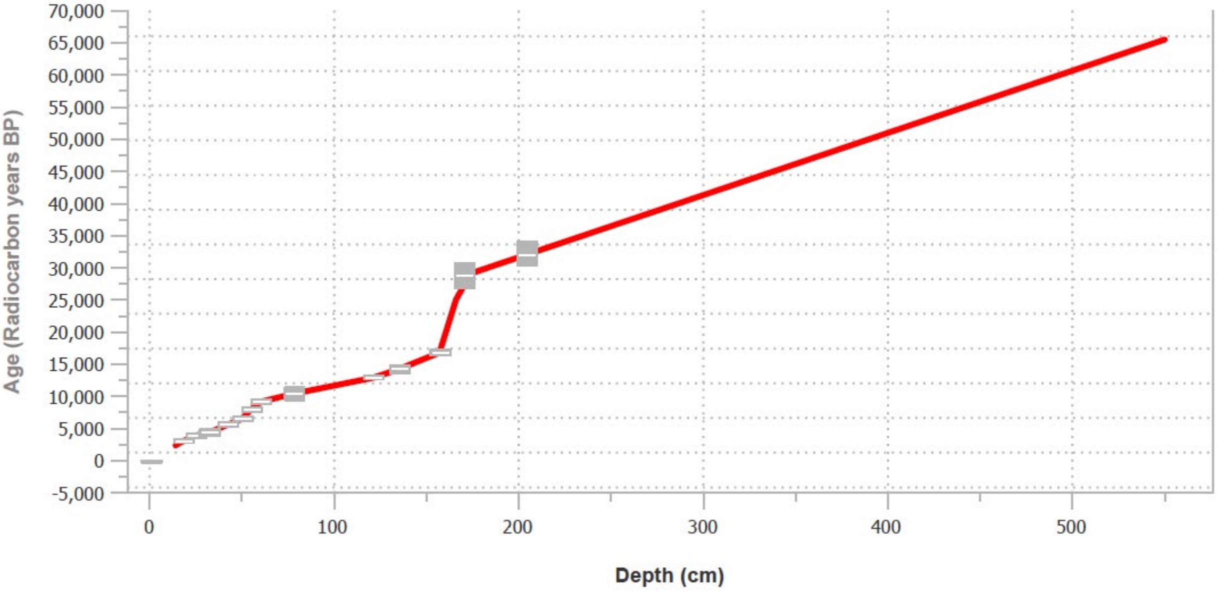

Supplement Figure 19: Lagoa Campestre de Salitre chronological model (DANS 1). Graph extracted from Neotoma database 07/2020[244]

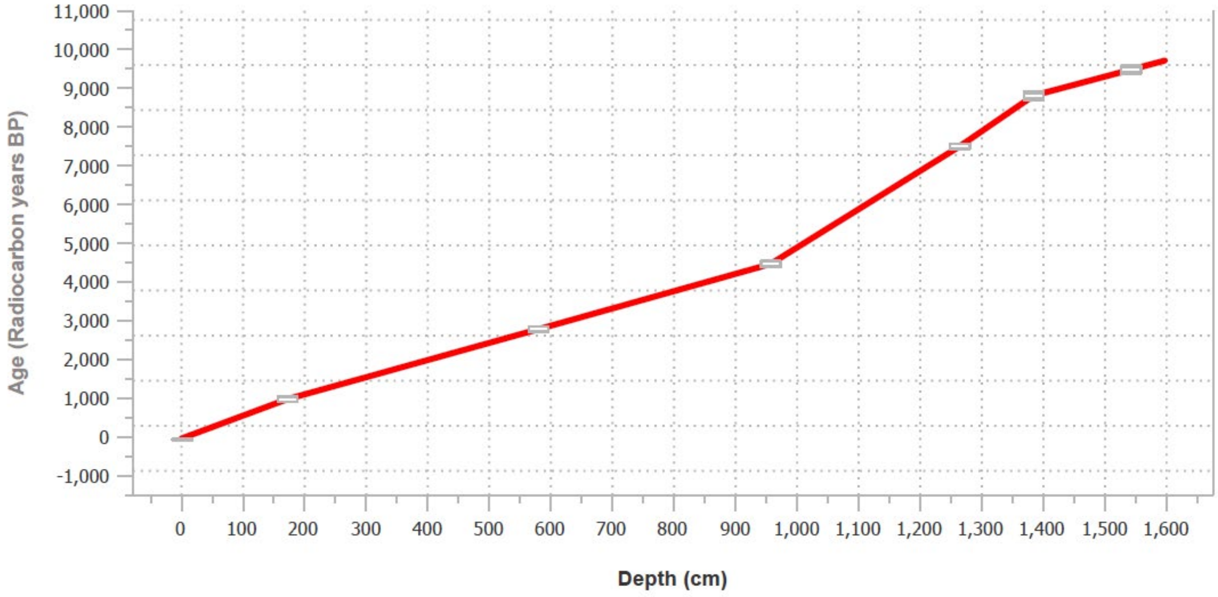

Supplement Figure 20: Lago do Pires chronological model (LADP 1). Graph extracted from Neotoma database 07/2020[244]

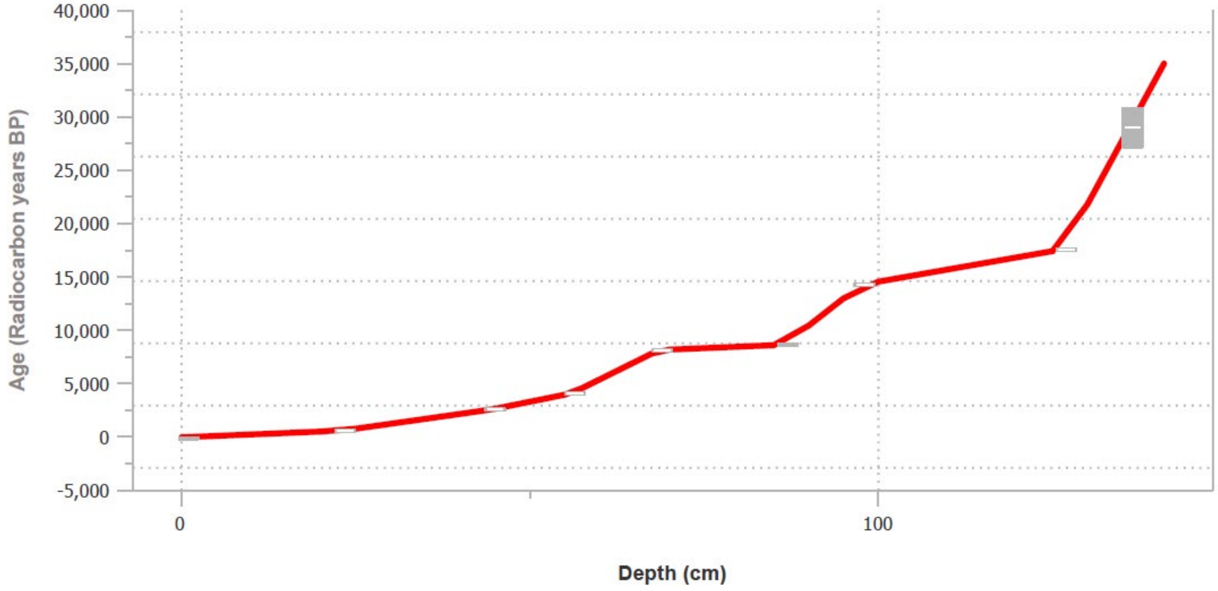

Supplement Figure 21: Morro de Itapeva chronological model (Behling 1). Graph extracted from Neotoma database 07/2020[244]

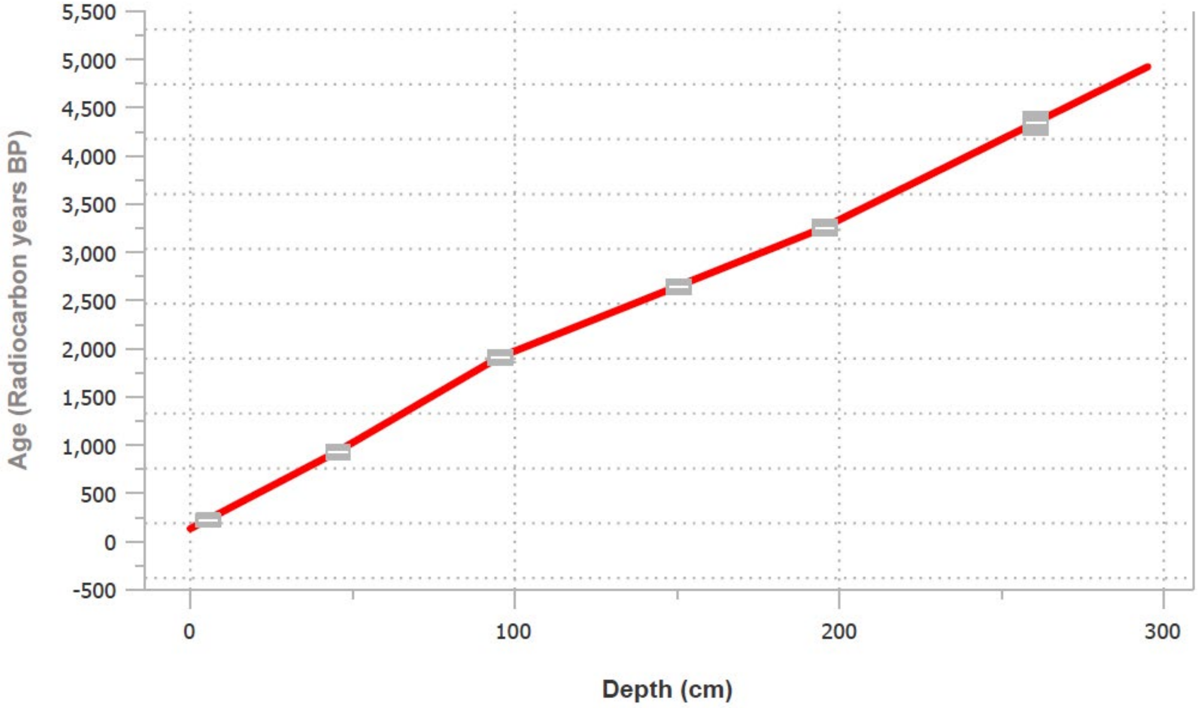

Supplement Figure 22: Laguna Piusbi chronological model (Behling et al 1998). Graph extracted from Neotoma database 07/2020[244]

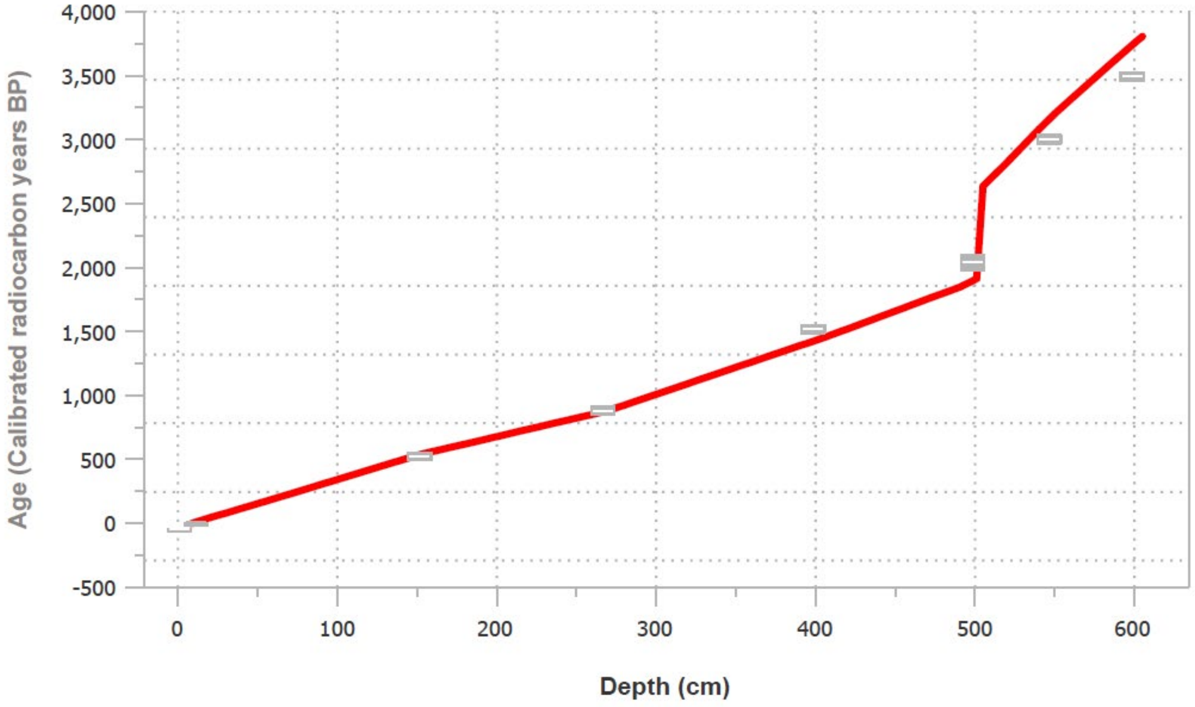

Supplement Figure 23: Laguna El Caimito chronological model (DANS 1). Graph extracted from Neotoma database 07/2020[244]

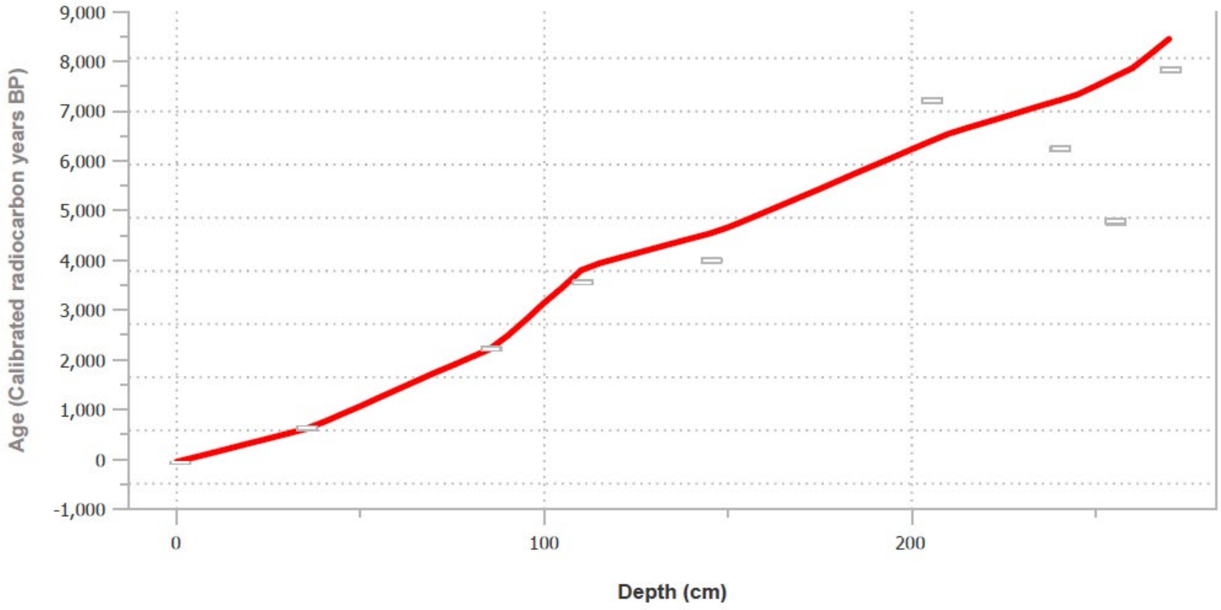

Supplement Figure 24: El Patía-2 chronological model (DANS 1). Graph extracted from Neotoma database 07/2020[244]

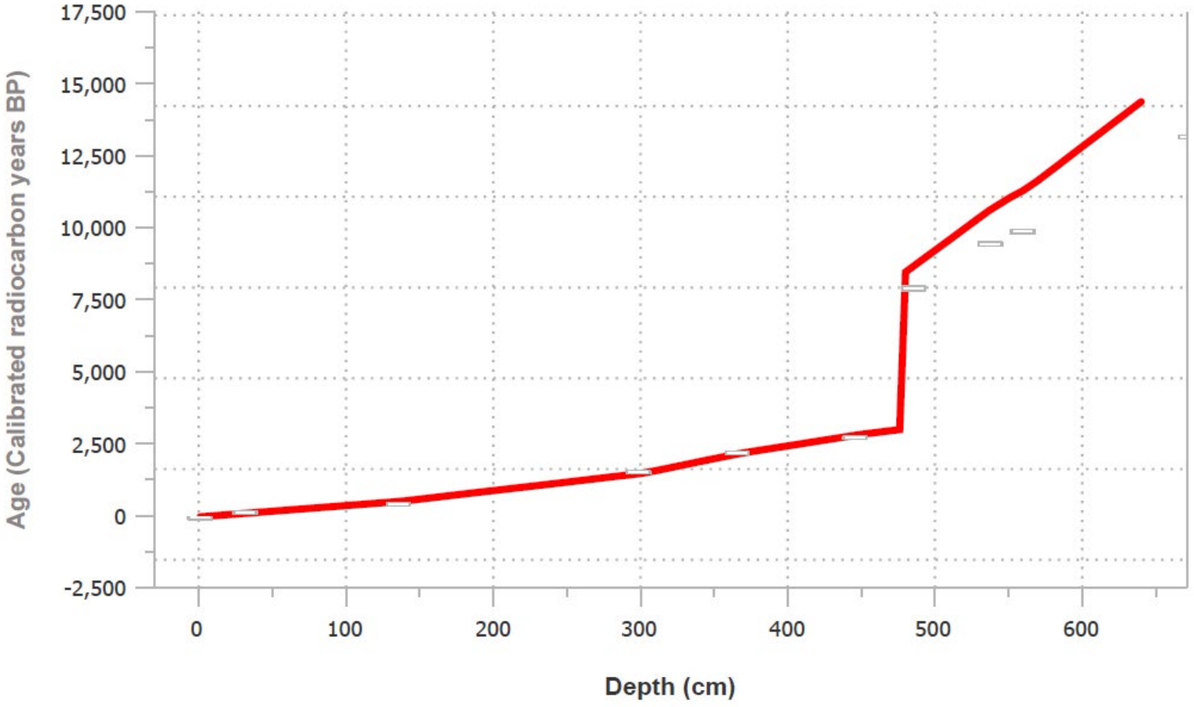

Supplement Figure 25: Quilichao-1 chronological model (DANS 1). Graph extracted from Neotoma database 07/2020[244]

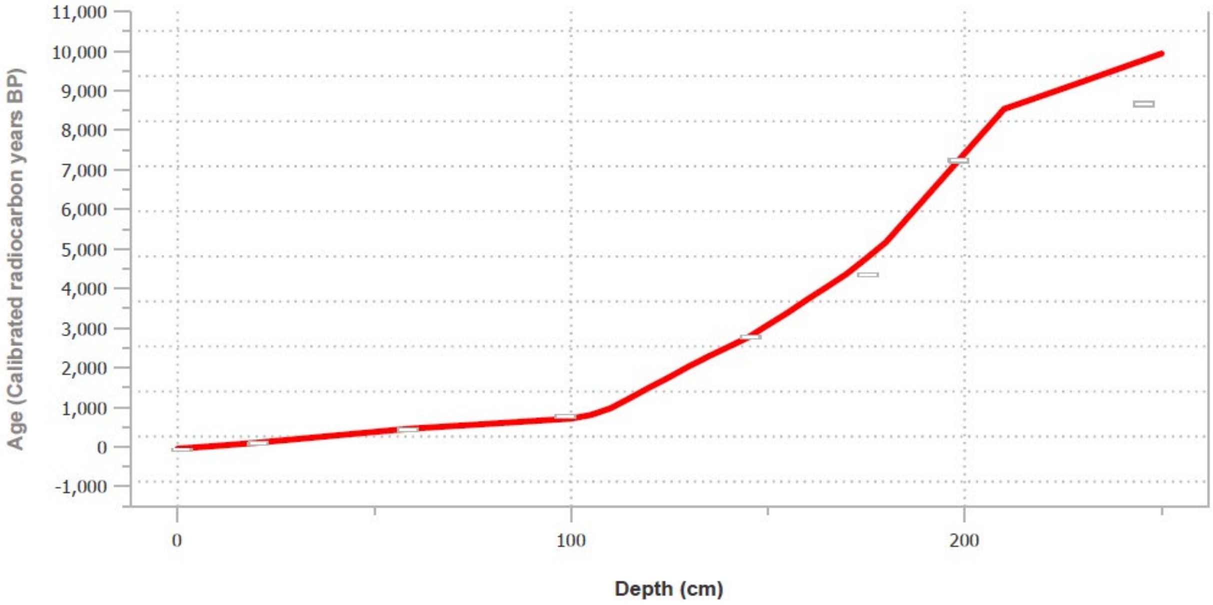

Supplement Figure 26: La Teta chronological model (DANS 1). Graph extracted from Neotoma database 07/2020[244]

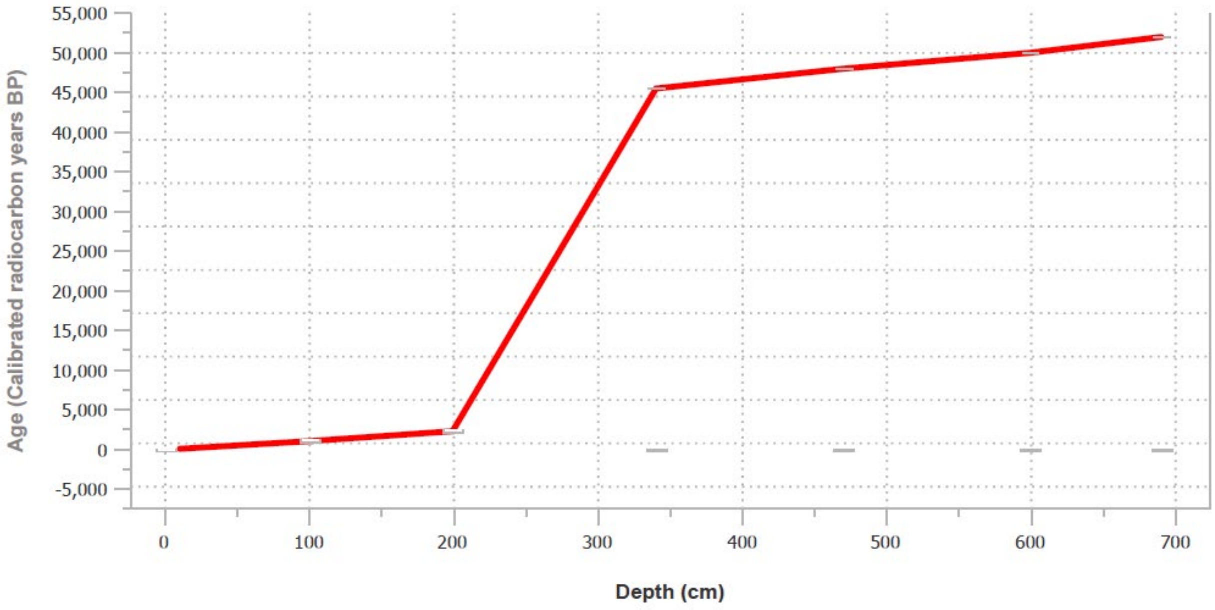

Supplement Figure 27: Pantano de Genagra chronological model (DANS 1/Wille et al. 2001). Graph extracted from Neotoma database 07/2020[244]

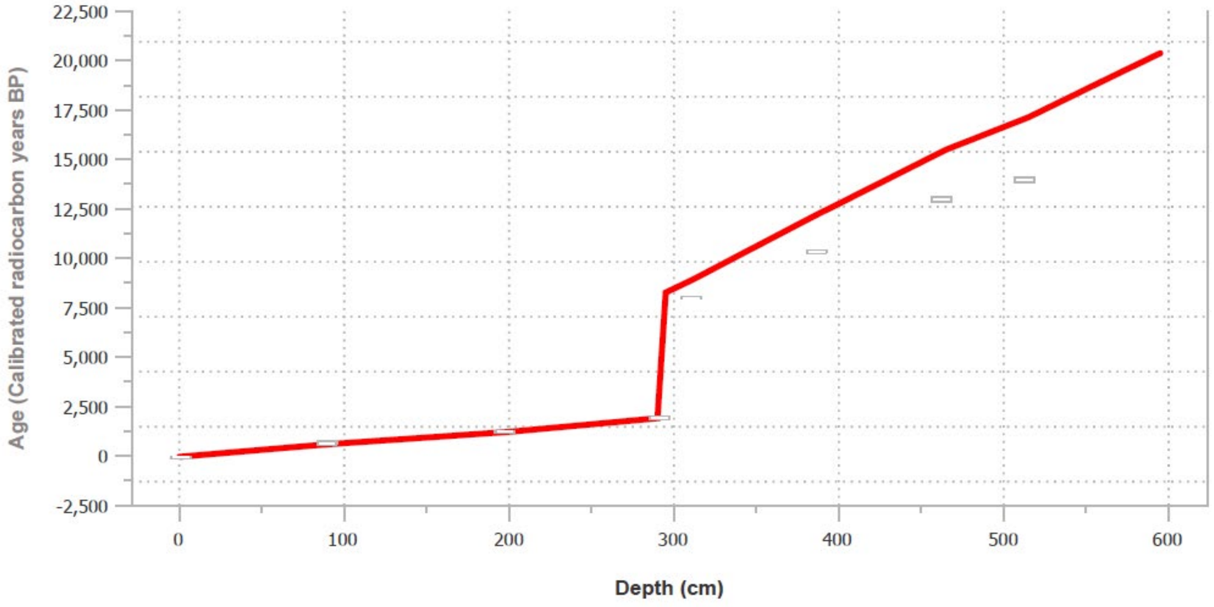

Supplement Figure 28: Piagua chronological model (DANS 1). Graph extracted from Neotoma database 07/2020[244]

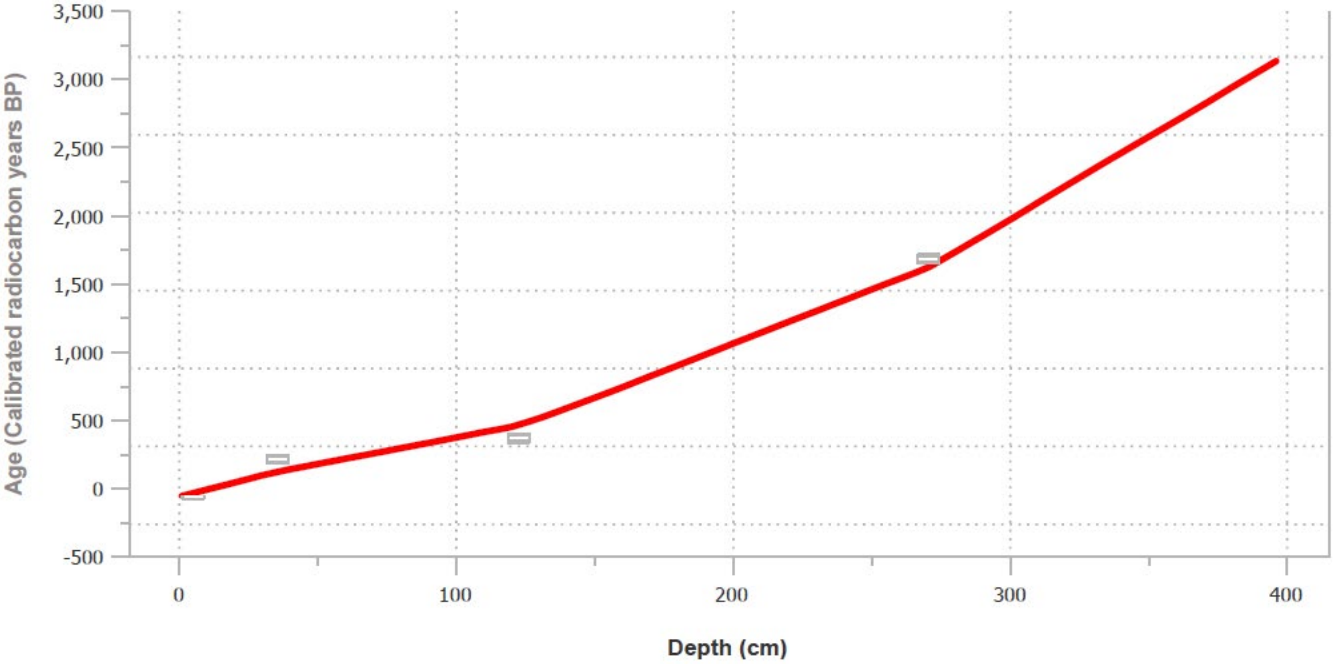

Supplement Figure 29: Laguna De La Cocha chronological model (DANS 1). Graph extracted from Neotoma database 07/2020[244]

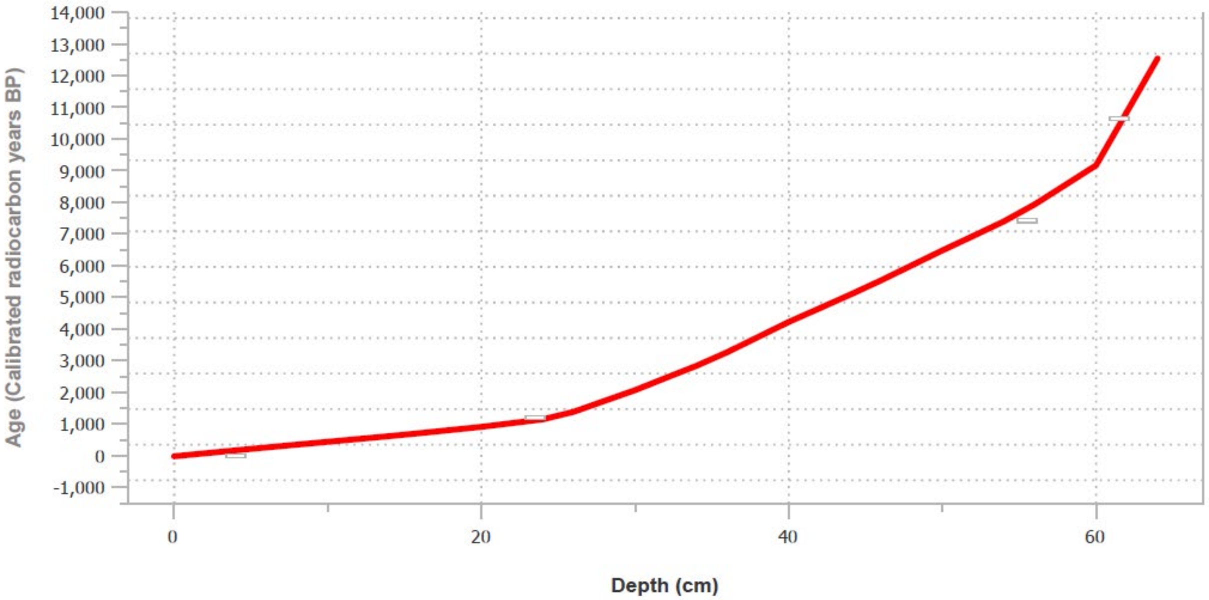

Supplement Figure 30: Laguna Cocha Caranga chronological model (LAPD 1). Graph extracted from Neotoma database 07/2020[244]

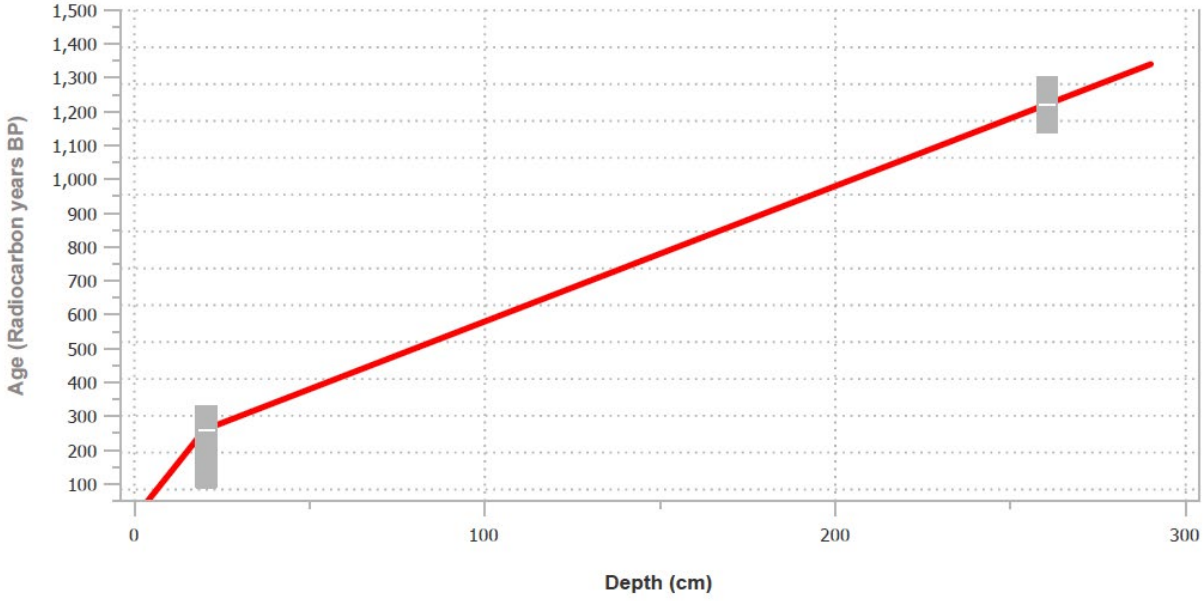

Supplement Figure 31: Paramo de Piedras Blancas (Rull et al 1987). Graph extracted from Neotoma database 07/2020[244]

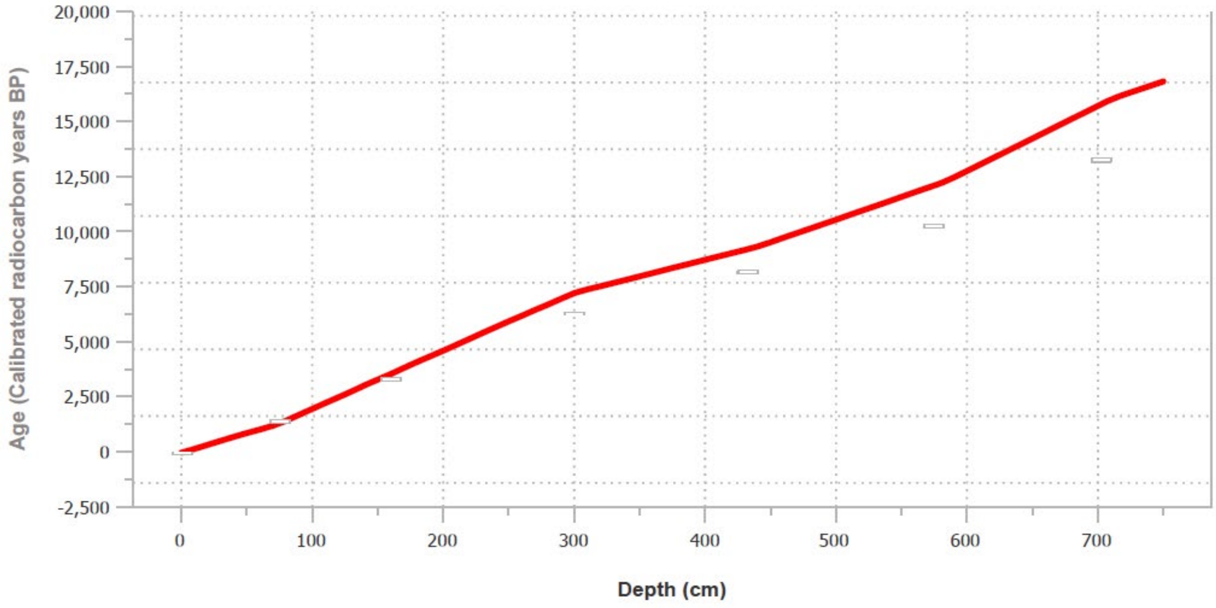

Supplement Figure 32: Llano Grande Mire chronological model (LAPD 1). Graph extracted from Neotoma database 07/2020[244]

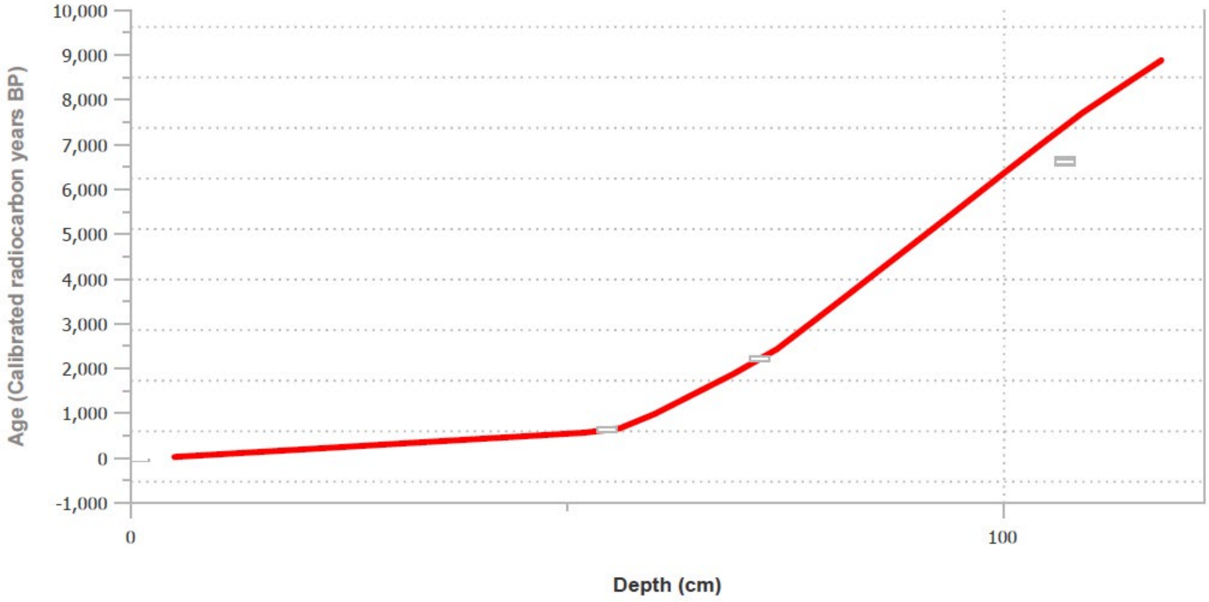

Supplement Figure 33: Páramo de Agua Blanca (DANS 1). Graph extracted from Neotoma database 07/2020[244]

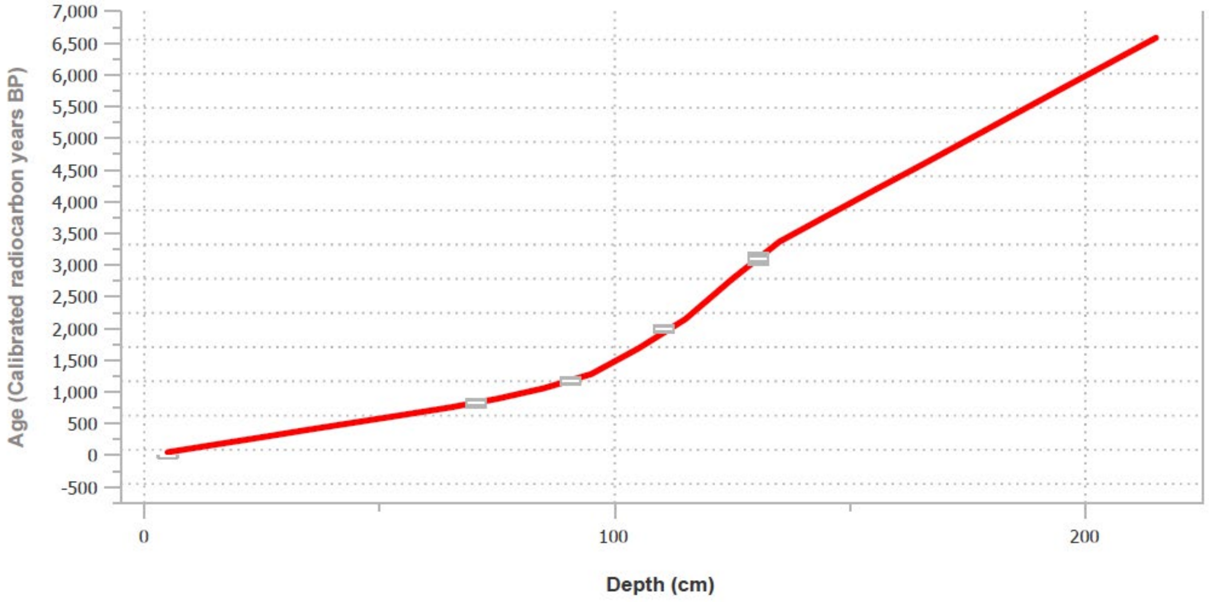

Supplement Figure 34: Laguna de los Bobos chronological model (DANS 1). Graph extracted from Neotoma database 07/2020[244]

Supplementary Tables

Supplement Table 1: Summary table of pre-Iberian land-use, the timing of Iberian contact and settlement, and Iberian-induced land-use- and population- change within the Spanish East Indies

Region	Pre-Iberian Land-use	Iberian control	Iberian contact	Post-Iberian land-use	Post-Iberian population dynamics
North Taiwan	Cultivation (rice, millet, tubers, cereals); foraging for tropical fruits; fishing & hunting (deer & game)[44]; resource trading between groups[43,47].	1626-1641 CE	1582 CE – Shipwreck of a Galleon going from Macau to Japan[48].	Focused on defense & establishing trading port with China[43].	Pulses of Dutch-introduced disease after 1632 CE. Population at 1640-1650 CE ~100,000[49]. Indigenous population at 1654 ~50,000[43].
The Philippines	Cultivation including swiddening, terracing & irrigation (taro, yam, rice, tubers, banana, coconut, sugarcane); foraging for tropical fruits; fishing & hunting; resource trading	1565-1898 CE	1521 CE – Spanish sighting of the Philippines; landing at Cebu	Introduction of land tenure; development of agricultural economies[50]. In places, resistance to Spanish rule led to socio-political and economic restructuring of Indigenous populations[65,66].	Between 1565 and 1591 CE the population of Luzon declined ~40% from from >1.0.5 million to 638,760[62,63]. Largest losses were recorded in Spanish-administered cities in Luzon and Visayas[71,72]
Maluku	Swidden cultivation ((?) *Coix, Cenchrus*, millet, and *Sorghum*[75]).<br>Selective cultivation of sago and coconut palms; resource trading; harvesting of marine resources.	1522-CE (Portuguese presence)<br>1606-1663 CE (Spanish presence)	1511 CE – Portuguese contact at Ternate; 1512 CE – Portuguese contact at Banda and Amboina	Focus on trading cloves & nutmeg in Spice Islands & Banda Islands (Dutch controlled)<br><br>Elsewhere, Spanish encouraged cultivation of 'New World' crops maize cassava; "New World" elephant ears (taro)	Unclear; 17% decline in central Maluku between 1634 and 1674 CE[77].

				(*Xanthosoma*); sweet potato[73].	
North Sulawesi	Swidden dryland cultivation and local wet-rice cultivation; hunting.	Between 1606 and 1616 – 1657 CE – Spanish presence[69]	1530 CE – Portuguese contact 1549 CE – Spaniards brought to Wenang.	Cultivation of maize; rice export	Unclear; a conservative estimate of 50,000 has been made for the region at both the start and end of the 17th century[71]. There appears to be a clear decrease in this number between 1644 and 1679 CE due to disease and impacts of Europeans on local social and economic structures[71].
Mariana Islands (Guam)	(?) swidden agriculture instigated from 950-350 BCE; "farm plots" at time of Spanish occupation; cultivation and agroforestry (banana, breadfruit, coconut, other fruit trees, sugarcane, swamp taro, taro and yam[56,86]); active soil improvement; harvesting of marine resources.	Claimed in 1565, Spanish presence from 1658 CE; settled between 1668-1815 CE.	1521 CE – Magellan made landfall in Guam[56]	Translocation of Chamorro into consolidated towns in southern Guam under *reducción* policies; introduction of maize, pigs and the Philippine Deer[94,95].	70% decline between 1668 (24,000-28,000 and 1699 (8100 people)[92]; complete depopulation of islands in the North Marianas (Aside from Rota) in 1700s[63]
West Caroline Islands (Palau)	Cultivation and agroforestry (tropical almond, banana, breadfruit, Malay apple, Tahitian chestnut, yam, taro, arrowroot and giant swamp taro[104,118]); harvesting of	1885-1899 CE (attempted) Spanish sovereign claim over Palau	1500s – European sighting & naming of Palau<br><br>1783 CE – British *Antelope*	1790 – British present chief with cattle, sheep, goats, pigs, poultry, ammunition[131]	1783 CE – 25000-50000[104,108];<br>55% decline (8,000 to 3700) in between ~1855 and 1885;<br>80% to 95% decline between 1783 and 1885[107]

	marine resources; terracing and wetland agriculture for taro.		shipwrecked off Palau	Large environmental impacts in the post-Spanish German and Japanese period (1899 to early 1900s), including forest clearance for coconut plantations and phosphate mining[113].	
West Caroline Islands (Yap)	Seasonal swidden agriculture; cultivation of salt-tolerant giant swamp taro (alluvial settings), arrowroot, taro, yams, *Pandanus,* coconut, bananas, breadfruit[118].	1731 CE – first Spanish mission.<br>1869 CE – German trading station<br>1874 to 1899 CE – Spanish sovereign claim over Yap	1525 CE (Portuguese)[293]	Copra and sea cucumber trade	Early to mid-1800s – as high as 51,000; 1899 CE – 7808[117] (~85% population decline over a century).
East Caroline Islands (Kosrae & Pohnpei)	Coconut, banana, breadfruit, taro, giant swamp taro, yams, arrowroot[118].	Spanish/ Portuguese missionaries in 1710 and 1731 CE killed[131]	1525-1526 CE – Portuguese (sighting?)[293]	Little direct disruption to land cultivation; introduction of livestock and the pig.	Kosrae –1830 to 1880 CE (peak whaling) – 90% decline (3000 to 300[132]); Pohnpei – 1840 to 1900 CE – 50% decline (10,000 to 5,000)[112]

Supplement Table 2: Summary table of pre-Iberian land-use, the timing of Iberian contact and settlement, and Iberian-induced land-use- and population- change within Spanish America

**Region**	**Pre-Iberian Land-use**	**Iberian control**	**Iberian contact**	**Post-Iberian land-use**	**Post-Iberian population dynamics**
Mexico (including Purépecha & Aztec [Triple Alliance])	Cultivation (including *milpa* swiddening) of maize[142] cacao, beans[143], squash (e.g. *Lagenaria siceraria, Cucurbita argyrosperma, C. moschata* and *C. pepo*)[140], tomatoes, gardens, fruit trees[141,144].	1521-1821 CE	1519 CE	Gold plundering & prospecting[147]; commercial pastoralism[147]; monocropping wheat, tobacco & sugarcane on flat areas (vs. pre-Spanish polyagriculture on terraced uplands)[147]; animals plowing (increased soil erosion)[147]; introduction of cattle, chickens, pigs, sheep, goats, oxen, horses, mules, burros.	3 to 52 million (centered on 20 million, including Yucatán peninsula); 90% decline after 1520. Smallpox arrives in 1520 CE and spreads inland; >50% of Aztec population died[111]
Central America other (Maya;) & Central America (other)	Salt mining; cultivation (including *milpa* swiddening and orchard-gardens) of cacao, chili, beans, squash, maize; selective cultivation of seasonal native plants (maguey, cotton, avocado, nance, allspice (*Pimenta dioica*), guava, sapodilla, mamey zapote)[144,159]; hunting[144].	1527 CE	1517 CE		
Caribbean	Fishing; cultivation (often using *conocu* raised plots and swiddening) of cotton, maize, cassava, sweet potato, chili pepper, achira, legumes, and yams; harvesting wild resources, most notably marunguey[294]	First Spanish settlement: 1494 CE - La Isabella & 1498 CE - Santo Domingo (Hispaniola); 1508 CE – Caparra (Puerto Rico);	1492 CE – Hispaniola, Cuba, Bahamas; 1494 (Cuba);	Cattle ranching[147]; development of commercial sugar and tobacco plantations; introduction of wheat, exotic fruit trees, grapevines; sheep, goats, pigs, cattle, dogs; harvesting brazil nut trees; gold mining[147]; demand for gold/ food/ spice tributes[147].	Smallpox arrives in Hispanola (1507 CE; major outbreak in 1517 CE), Puerto Rico (1518 CE), and Cuba (1519 CE), removing 50% of the population[111]

		1514 CE – Havana (Cuba).			
Amazonian tropical forests	Cropping (including low intensity fire) of *Cucurbita* sp., maize, rice, cassava, peanut and chili[32,174,178]. Selective forest tree cultivation (and plant exclusion) within ADEs, including fruit trees (Agavaceae, C*aryocar, Byrsonima, Theobroma cacao* and Lecythidaceae) and various palms (*Mauritia, Attalea, Astrocaryum, Euterpe, Elaeis*, and *Oenocarpus*)[174,187-189].	1570-1600 CE - Establishment of Jesuit missions in Amazonia; 1616 CE establishment of Forte do Presépio (Portuguese)	1500 CE – Bahia, Brazil (Portuguese) 1541 CE – penetration of Amazon from Ecuador via Rio Napo.	Rubber tapping and harvesting of brazil nut tree (*Caesalpinia echinata)*. Impact limited to Amazon delta and riparian zones; extraction of *drogas do sertão*)[197]	90% population decline[32]. Smallpox in Brazil in 1555 CE[111]; Large epidemic in 1563 spreads from the coast inland along river corridors[111].
Los Llanos	Cultivation of maize[217] and cassava[199] using swidden farming, raised mounds, ridged fields, and causeways[218].	Sparse settlement in the Orinoco Valley by 1570 CE[221].	1498 CE – Orinoco Valley, Venezuela (Spanish) [221].	1500s - Cattle grazing and palm cultivation; 1600s – intensive husbandry and cultivation of sugarcane, tobacco, cacao and coffee in the Orinoco watershed[221]..	Exact estimates are unclear but, likely in the 20-60% range. The estimated pre-Spanish population of Venezuela is ~200,000 to 500,000 (pre-Spanish), and the population at the end of the 16th century is placed at 200,000 to 300,000[221]. Major smallpox epidemic in 1580 CE[221].
Llanos de Moxos	Raised mound cultivation of maize, squash, sweet potato, peanuts[198], *Inga*[205], taro, cassava, "New World" elephant ears/ taro (*Xanthosoma);*	Jesuit missions established from mid-1600s[213].	Protracted contact from 1583 CE[213]	Establishment of cattle and dairy farming; forced relocations of Indigenous populations into missions[214].	Indigenous population decline between the pre-Spanish and mission period in the Llanos de Moxos is estimated at

	annatto *(Bixa orellana), Ilex*[204]; wild game hunting; construction of large earthworks and palisades for defensive purposes[207].				~70% (350,000 to 100,000)[215].
Brazilian Cerrado	Maize, squash, beans, cassava and native fruits (*Caryocar brasiliense; Byrsonima crassifólia; Mauritia flexuosa; Hancornia speciose)*; artificial cultivation of fertile/ forest plots[226].	First mining settlements in early 1700s[228].	Mid 1550 CE – Portuguese search Cerrado for mineral resources and slaves[228].	Limited (may have served as a refuge for Indigenous groups who were being actively killed and enslaved by the Portuguese in the Atlantic Forest region[147]).	Disease outbreak in the mid-1500s spread inland from the Atlantic coast (see below)[111]. Population estimates are unclear.
Brazilian Atlantic Forests	Hunting and fishing; cultivation (including swiddening) of cassava, beans, peanuts, tobacco, and maize.	1530 CE - São Vicente; São Paulo.<br>1549 CE - Salvador	1500 CE (Atlantic Forests)	Hunting for pelts; overharvesting of plant resources including the brazil nut tree, other hardwood (for fuel & ship building) and ipecac; clearance for cattle raising and farming (inc. tobacco and sugar cane plantations); gold/diamond mining (Minas Gerais)[147]; active genocide of Indigenous populations forced transition of “traditional” cultivation to a more flexible and mobile system[147].	Large scale epidemics in the mid-1500s, including smallpox (1555 CE[111]; 1563, which spread from the coast inland along river corridors[111]), hemorrhagic dysentery, influenza, the plague, smallpox, and measles[147].<br>Population decline >66% by 1570 CE[147].
Pacific Forests	Marine harvesting and fishing; cultivation (including, in places, raised field agriculture) of squash, cassava and, potentially, palm	1630s – Barbacoas conquered.	Early (and failed) mining attempts between 1573	16th and 17th century gold, silver and platinum mining in the Upper Chocó (North Colombia) and Barbacoas (South Colombia);	Population decline within the Spanish mining areas dropped 90%, from 35,000 to 3,850 between the late

	forests[219,233,234]; placer gold mining.		and mid-1590 CE[236,237]	The harsh environmental conditions imposed by the pluvial rainforests meant that Spanish had little control of the Pacific Forests outside of the mining regions[236]; black and Indigenous "maroon" communities cultivated maize and Old World crops (taro, sugar cane and breadfruit) on river levees for subsistence activities and markets[239].	16th and late 17th centuries[236]; little is known about the population of the Pacific Forests outside of this region.
Andes (500-3,500 m ASL)	Aboriculture (cherimoya. papaya de monte, pepino, tree tomato, lúcuma, pacae, ciruela de fraile, guava, achiote and avocado[224,232]; cultivation (using terraces) of maize, achira, llerén, arrowroot, cassava, sweet potato, squash, chili and cacao[224,232]; metal mining[233].	1537 – Popayán established; 1538 CE – Bogotá established.	Conquest of Muisca between 1537 and 1543 CE (New Grenada); Conquest of Inka Empire between 1532 and 1570 CE	Precious gem and metal (including silver and gold placer deposits in the Cauca) mining; cattle, sheep and horse grazing[241]; large-scale population turnover and social reorganisation[235].	16th century Indigenous population decline in the northern Andes estimated at 50 to 70%[241]. This value may, however, be conservative. For example, the combined population of the Andes and Pacific lowlands in the 1400s was estimated as up to 11.5 million people. By 1620, this population was estimated at 600,000 (a drop of 80%)[243].<br><br>Smallpox reaches the Andes in 1524-1527 CE[111]
Andes (2,300-3,500m ASL)	Terracing for maize and other crops (as above); metal mining[233].				
Andes >3,500-4,000m ASL)	Cultivation of upper montane crop specialists including quinoa, cañihua, amaranth, lupin, fava beans/haba, oca, ulluco, potatoes, mashwa, jicama[224,232]; metal mining[233].				

Supplement Table 3: Sample ID, depth, and 14C age of samples extracted from 445 cm long sediment core extracted from Lake Bululacao, the Philippines. Samples were analysed for ^{14}C at the Australian National University Research School of Earth Sciences radiocarbon laboratory

Sample ID	Depth (cm)	Sample type & pre-treatment	$F^{14}C$	F^{14} error (±)	^{14}C age	^{14}C error (±)
17407	100	Plant (leaf, flower bud) macrofossils pre-treated with HCl-NaOH-HCl (A-B-A) washes	0.9247	0.0029	630	35
17409	150		0.8635	0.0027	1180	35
17410	220		0.7851	0.0027	1945	35
17411	270		0.7523	0.0025	2285	35
17412	310		0.7154	0.0026	2690	35
17413	335		0.6693	0.0023	3225	35
17414	360		0.6894	0.0023	2985	35
17416	390		0.6527	0.0023	3425	35
17417	410		0.6250	0.0023	3775	35
17418	430		0.6239	0.0022	3790	35

References

1 Wang, Y. *et al.* The Holocene Asian monsoon: links to solar changes and North Atlantic climate. *Science* **308**, 854-857, doi:10.1126/science.1106296 (2005).

2 Field, E. *et al.* Coherent patterns of environmental change at multiple organic spring sites in northwest Australia: Evidence of Indonesian-Australian summer monsoon variability over the last 14,500 years. *Quaternary Science Reviews* **196**, 193-216, doi:https://doi.org/10.1016/j.quascirev.2018.07.018 (2018).

3 Moffa-Sanchez, P., Rosenthal, Y., Babila, T. L., Mohtadi, M. & Zhang, X. Temperature Evolution of the Indo-Pacific Warm Pool Over the Holocene and the Last Deglaciation. *Paleoceanography and Paleoclimatology* **34**, 1107-1123, doi:10.1029/2018pa003455 (2019).

4 Rosenthal, Y., Linsley, B. K. & Oppo, D. W. Pacific Ocean Heat Content During the Past 10,000 Years. *Science* **342**, 617, doi:10.1126/science.1240837 (2013).

5 Oppo, D. W., Rosenthal, Y. & Linsley, B. K. 2,000-year-long temperature and hydrology reconstructions from the Indo-Pacific warm pool. *Nature* **460**, 1113-1116 (2009).

6 Lu, Z., Liu, Z., Zhu, J. & Cobb, K. M. A Review of Paleo El Niño-Southern Oscillation. *Atmosphere* **9**, doi:10.3390/atmos9040130 (2018).

7 Zhang, P. *et al.* A Test of Climate, Sun, and Culture Relationships from an 1810-Year Chinese Cave Record. *Science* **322**, 940-942 (2008).

8 Shi, F., Li, J. & Wilson, R. J. A tree-ring reconstruction of the South Asian summer monsoon index over the past millennium. *Scientific reports* **4**, 6739, doi:10.1038/srep06739 (2014).

9 Sachs, J. P. *et al.* Southward movement of the Pacific intertropical convergence zone AD 1400–1850. *Nature Geoscience* **2**, 519-525, doi:10.1038/ngeo554 (2009).

10 Wang, L. C. *et al.* Late Holocene environmental reconstructions and their implications on flood events, typhoon, and agricultural activities in NE Taiwan. *Climate of the Past* **10**, 1857-1869, doi:10.5194/cp-10-1857-2014 (2014).

11 Nunn, P. Environmental Catastrophe in the Pacific Islands around A.D. 1300. *Geoarchaeology* **15**, 715-740 (2000).

12 Newton, A., Thunell, R. & Stott, L. Climate and hydrographic variability in the Indo-Pacific Warm Pool during the last millennium. *Geophysical Research Letters* **33**, doi:10.1029/2006gl027234 (2006).

13 Richey, J. N. & Sachs, J. P. Precipitation changes in the western tropical Pacific over the past millennium. *Geology* **44**, 671-674, doi:10.1130/g37822.1 (2016).

14 Yan, H. *et al.* Dynamics of the intertropical convergence zone over the western Pacific during the Little Ice Age. *Nature Geoscience* **8**, 315-320, doi:10.1038/ngeo2375 (2015).

15 Dey, R., Lewis, S. C., Arblaster, J. M. & Abram, N. J. A review of past and projected changes in Australia's rainfall. *Wiley Interdisciplinary Reviews: Climate Change* **10**, doi:10.1002/wcc.577 (2019).

16 Flantua, S. G. A. *et al.* Climate variability and human impact in South America during the last 2000 years: synthesis and perspectives from pollen records. *Climate of the Past* **12**, 483-523, doi:10.5194/cp-12-483-2016 (2016).

17 Hodell, D. A., Curtis, J. H. & Brenner, M. Possible role of climate in the collapse of Classic Maya civilization. *Nature* **375**, 391.

18 Goman, M. & Byrne, R. A 5000-year record of agriculture and tropical forest clearance in the Tuxtlas, Veracruz, Mexico. *The Holocene*, 83-89 (1998).

19 Iriarte, J. & Behling, H. The expansion of Araucaria forest in the southern Brazilian highlands during the last 4000 years and its implications for the development of the Taquara/Itararé Tradition. *Environmental Archaeology* **12**, 115-127, doi:10.1179/174963107x226390 (2007).

20 Raczka, M. F., De Oliveira, P. E., Bush, M. & McMichael, C. H. Two paleoecological histories spanning the period of human settlement in southeastern Brazil. *Journal of Quaternary Science* **28**, 144-151, doi:10.1002/jqs.2597 (2013).

21 Lachniet, M. S., Bernal, J. P., Asmerom, Y., Polyak, V. & Piperno, D. A 2400 yr Mesoamerican rainfall reconstruction links climate and cultural change. *Geology* **40**, 259-262, doi:10.1130/g32471.1 (2012).

22 Wu, H. C. *et al.* Changes to Yucatan Peninsula precipitation associated with salinity and temperature extremes of the Caribbean Sea during the Maya civilization collapse. *Sci Rep* **7**, 15825, doi:10.1038/s41598-017-15942-0 (2017).

23 Rojas, M., Arias, P. A., Flores-Aqueveque, V., Seth, A. & Vuille, M. The South American monsoon variability over the last millennium in climate models. *Climate of the Past* **12**, 1681-1691, doi:10.5194/cp-12-1681-2016 (2016).

24 Novello, V. F. *et al.* Two Millennia of South Atlantic Convergence Zone Variability Reconstructed From Isotopic Proxies. *Geophysical Research Letters* **45**, 5045-5051, doi:10.1029/2017gl076838 (2018).

25 Bird, B. W. *et al.* A 2,300-year-long annually resolved record of the South American summer monsoon from the Peruvian Andes. *Proc Natl Acad Sci U S A* **108**, 8583-8588, doi:10.1073/pnas.1003719108 (2011).

26 Azevedo, V. *et al.* Medieval Climate Variability in the eastern Amazon-Cerrado regions and its archeological implications. *Sci Rep* **9**, 20306, doi:10.1038/s41598-019-56852-7 (2019).

27 Behling, H., Cohen, M. C. L. & Lara, R. J. Studies on Holocene mangrove ecosystem dynamics of the Bragança Peninsula in north-eastern Pará, Brazil. *Palaeogeography, Palaeoclimatology, Palaeoecology* **167**, 225-242, doi:https://doi.org/10.1016/S0031-0182(00)00239-X (2001).

28 Hodell, D. A. *et al.* Climate change on the Yucatan Peninsula during the Little Ice Age. *Quaternary Research* **63**, 109-121, doi:10.1016/j.yqres.2004.11.004 (2005).

29 Polissar, P. J. *et al.* Solar modulation of Little Ice Age climate in the tropical Andes. *Proceedings of the National Academy of Sciences* **103**, 8937-8942, doi:10.1073/pnas.0603118103 (2006).

30 Ledru, M. P. *et al.* The Medieval Climate Anomaly and the Little Ice Age in the eastern Ecuadorian Andes. *Climate of the Past* **9**, 307-321, doi:10.5194/cp-9-307-2013 (2013).

31 Cai, W. *et al.* Climate impacts of the El Niño–Southern Oscillation on South America. *Nature Reviews Earth & Environment* **1**, 215-231, doi:10.1038/s43017-020-0040-3 (2020).

32 Koch, A., Brierley, C., Maslin, M. M. & Lewis, S. L. Earth system impacts of the European arrival and Great Dying in the Americas after 1492. *Quaternary Science Reviews* **207**, 13-36, doi:10.1016/j.quascirev.2018.12.004 (2019).

33 Blust, R. The prehistory of the Austronesian-speaking peoples: A view from language. *Journal of World Prehistory* **9**, 453-510, doi:10.1007/BF02221119 (1995).

34 Bellwood, P. *First farmers: The origins of agricultural societies*. (Blackwell, 2005).

35 Deng, Z. *et al.* The first discovery of Neolithic rice remains in eastern Taiwan: phytolith evidence from the Chaolaiqiao site. *Archaeological and Anthropological Sciences* **10**, 1477-1484, doi:10.1007/s12520-017-0471-z (2017).

36 Corlett, R. *The Ecology of Tropical East Asia*. (Oxford University Press, 2014).

37 Spriggs, M. Archaeology and the Austronesian expansion: where are we now? *Antiquity* **85**, 510-528. (2011).

38 Barton, H. The Case for Rainforest Foragers: The Starch Record at Niah Cave, Sarawak. *Asian Perspectives* **44**, 56-72 (2005).

39 Hunt, C. O. & Rabett, R. J. Holocene landscape intervention and plant food production strategies in island and mainland Southeast Asia. *Journal of Archaeological Science* **51**, 22-33, doi:10.1016/j.jas.2013.12.011 (2014).

40 Tromp, M. *et al.* Exploitation and utilization of tropical rainforests indicated in dental calculus of ancient Oceanic Lapita culture colonists. *Nat Hum Behav* **4**, 489-495, doi:10.1038/s41562-019-0808-y (2020).

41 Maxwell, J. J., Howarth, J. D., Vandergoes, M. J., Jacobsen, G. E. & Barber, I. G. The timing and importance of arboriculture and agroforestry in a temperate East Polynesia Society, the Moriori, Rekohu (Chatham Island). *Quaternary Science Reviews* **149**, 306-325, doi:10.1016/j.quascirev.2016.08.006 (2016).

42 Klamer, M. The dispersal of Austronesian languages in Island South East Asia: Current findings and debates. *Language and Linguistics Compass* **13**, doi:10.1111/lnc3.12325 (2019).

43 Chiu, H. *The colonial 'civilizing process' in Dutch Formosa 1624-1662* PhD thesis, Leiden University, (2008).

44 Hung, H. & Carson, M. T. Foragers, fishers and farmers: origins of the Taiwanese Neolithic. *Antiquity* **88**, 1115-1131 (2014).

45 Borao, J. E. & Hung, H. C. *Recovering the Past of Jilong: New Archaeological Findings from Heping Island of Northern Taiwan*. (SMC Publishing Inc, 2015).

46 Kuo, S. in *New Frontiers in the Neolithic Archaeology of Taiwan (5600–1800 BP)* Vol. 3 *The Archaeology of Asia-Pacific Navigation* (Springer).

47 Cruz Berrocal, M. *et al.* A Comprised Archaeological History of Taiwan through the Long-Term Record of Heping Dao, Keelung. *International Journal of Historical Archaeology* **22**, 905-940, doi:10.1007/s10761-017-0453-y (2018).

48 Mateo, B. & Eugenio, J. *The Spanish experience in Taiwan, 1626-1642: the Baroque ending of a Renaissance endeavor*. (Hong Kong University Press, 2009).

49 Andrade, T. *How Taiwan Became Chinese: Dutch, Spanish, and Han Colonization in the Seventeenth Century*. (Columbia University Press, 2008).

50 Amano, N., Bankoff, G., Findley, D. M., Barretto-Tesoro, G. & Roberts, P. Archaeological and historical insights into the ecological impacts of pre-colonial and colonial introductions into the Philippine Archipelago. *The Holocene*, doi:10.1177/0959683620941152 (2020).

51 Bellwood, P. *The prehistory of the Indo-Pacific archipelago*. 2 edn, (University of Hawai'i Press, 1997).

52 Denham, T. in *The Oxford Handbook of Prehistoric Oceania* (eds T. L. Hunt & E. E. Cochrane) 48-68 (2018).

53 Bellwood, P. *First Islanders: Prehistory and Human Migration in Island Southeast Asia*. (John Wiley and Sons, 2017).

54 Acabado, S. The Archaeology of Pericolonialism: Responses of the "Unconquered" to Spanish Conquest and Colonialism in Ifugao, Philippines. *International Journal of Historical Archaeology* **21**, 1-26, doi:10.1007/s10761-016-0342-9 (2016).

55 Amano, N., Piper, P. J., Hung, H.-c. & Bellwood, P. Introduced Domestic Animals in the Neolithic and Metal Age of the Philippines: Evidence From Nagsabaran, Northern Luzon. *The Journal of Island and Coastal Archaeology* **8**, 317-335, doi:10.1080/15564894.2013.781084 (2013).
56 Dixon, B., Schaefer, R. & McCurdy, T. Traditional Chamorro Farming Innovations During the Spanish and Philippine Contact Period on Northern Guam. *Philippine Quarterly of Culture and Society* **38**, 291-321 (2010).
57 Griffin, A. & Griffin, B. in *Adaptive Strategies and Change in Philippine Swidden-based Societies* (ed H. Olofson) 55-72 (PDM Press, 1981).
58 Dyble, M., Thorley, J., Page, A. E., Smith, D. & Migliano, A. B. Engagement in agricultural work is associated with reduced leisure time among Agta hunter-gatherers. *Nat Hum Behav* **3**, 792-796, doi:10.1038/s41562-019-0614-6 (2019).
59 Junker, L. L. *Raiding, Trading, and Feasting: The Political Economy of Philippine Chiefdoms*. (Ateneo de Manila University Press, 2000).
60 Stevenson, J., Siringan, F., Finn, J. A. N., Madulid, D. & Heijnis, H. Paoay Lake, northern Luzon, the Philippines: a record of Holocene environmental change. *Global Change Biology* **16**, 1672-1688, doi:10.1111/j.1365-2486.2009.02039.x (2010).
61 Newson, L. A. Disease and immunity in the pre-Spanish Philippines. *Social Science & Medicine* **48**, 1833-1850, doi:https://doi.org/10.1016/S0277-9536(99)00094-5 (1999).
62 Newson, L. A. Conquest, pestilence and demographic collapse in the early Spanish Philippines. *Journal of Historical Geography* **32**, 3-20, doi:https://doi.org/10.1016/j.jhg.2004.08.001 (2006).
63 Newson, L. A. *Conquest and Pestilence in the Early Spanish Philippines*. (University of Hawaii Press, 2009).
64 Bankoff, G. in *The horse as cultural icon: the real and the symbolic horse in the early modern world* (eds Peter Edwards, Karl AE Enenkel, & Elspeth Graham) 91-120 (Brill, 2011).
65 Acabado, S. B. *et al.* The Short History of the Ifugao Rice Terraces: A Local Response to the Spanish Conquest. *Journal of Field Archaeology* **44**, 195-214, doi:10.1080/00934690.2019.1574159 (2019).
66 Olofson, H. *Adaptive strategies and change in Philippine Swidden-based societies*. (Forest Research Institute, 1981).
67 Phelan, J. L. *The Hispanization of the Philippines : Spanish aims and Filipino responses 1565-1700*. (University of Wisconsin Press, 1959).
68 Larkin, J. A. Philippine History Reconsidered: A Socioeconomic Perspective. *The American Historical Review,* **87,**, 595-628 (1982).
69 Wigboldus, J. S. A History of the Minahasa c. 1615-1680. *Archipel* **34**, 63-101, doi:10.3406/arch.1987.2374 (1987).
70 Wuisang, C. E. V. & Jones, D. S. in *World Planning Schools Congress 4-8 July 2011.*
71 Henley, D. *Fertility, food and fever : population, economy and environment in North and Central Sulawesi, 1600-1930*. (KITLV Press, 2005).
72 Bellwood, P. *Terra Australis 50: The Spice Islands in Prehistory*. (ANU Press, 2019).
73 Ellen, R. The human consequences of deforestation in the Moluccas. *Civilisations: Revue internationale d'anthropologie et de sciences humaines* **44**, 176-193 (1997).
74 Suparan, P., Dam, R. A. C., van der Kaars, S. & Wong, T. E. Late Quaternary tropical lowland environments on Halmahera, Indonesia. *Palaeogeography, Palaeoclimatology, Palaeoecology* **171**, 229-258, doi:https://doi.org/10.1016/S0031-0182(01)00247-4 (2001).

75 Visser, L. E. *Social and territorial aspects of swidden cultivation in Sahu, Eastern Indonesia*. (Foris Publications, 1989).

76 Villiers, J. Manila and Maluku: Trade and Warfare in the Eastern Archipelago 1580 – 1640. *Philippine Studies* **34**, 146-161 (1986).

77 Reid, A. The Seventeenth-Century Crisis in Southeast Asia. *Modern Asian Studies* **24**, 639-659 (1990).

78 Kratoska, P. H. *South East Asia, Colonial History: Imperialism before 1800*. Vol. 1 (Routledge, 2001).

79 Carson, M. T. *Archaeological Landscape Evolution: The Mariana Islands in the Asia-Pacific Region*. (Springer, 2016).

80 Hung, H. *et al.* The first settlement of remote oceania: The Philippines to the Marianas. *Antiquity* **85**, 909-926 (2011).

81 Athens, J. S. & Ward, J. V. Holocene Vegetation, Savanna Origins and Human Settlement of Guam. *Records of the Australian Museum Supplement* **29**, 15-30 (2004).

82 Carson, M. T. & Kurashina, H. Re-envisioning long-distance Oceanic migration: early dates in the Mariana Islands. *World Archaeology* **44**, 409-435 (2012).

83 Dye, T. & Cleghorn, P. L. Prehistoric Use of the Interior of Southern Guam. *Micronesica Supplement* **2**, 261-274 (1990).

84 Graves, M. W. Organization and Differentiation within Late Prehistoric Ranked Social Units, Mariana Islands, Western Pacific. *Journal of Field Archaeology* **13**, 139-154, doi:10.2307/530217 (1986).

85 Hunter-Anderson, R. L. Cultural Responses to a Late Holocene Climatic Oscillation in the Mariana Islands, Micronesia: Lessons from the Past. *Research in Human Ecology* **17**, 148-159 (2010).

86 Horrocks, M., Peterson, J. & Carson, M. T. Pollen, Starch, and Biosilicate Analysis of Archaeological Deposits on Guam and Saipan, Mariana Islands, Northwest Pacific: Evidence for Chamorro Subsistence Crops and Marine Resources. *The Journal of Island and Coastal Archaeology* **10**, 97-110, doi:10.1080/15564894.2014.921960 (2015).

87 Rogers, R. F. *Destiny's Landfall: A History of Guam*. 380 (University of Hawaii Press, 1995).

88 Athens, J. S. Latte Period Occupation on Pagan and Sarigan, Northern Mariana Islands. *The Journal of Island and Coastal Archaeology* **6**, 314-330, doi:10.1080/15564894.2011.555806 (2011).

89 Hunter-Anderson, R. Savanna anthropogenesis in the Mariana Islands, Micronesia: re-interpreting the palaeoenvironmental data. *Archaeol. Oceania* **44**, 125-141 (2009).

90 Moore, D. R. Archaeological Evidence of a Prehistoric Farming Technique on Guam. *Micronesica* **38**, 93-101 (2005).

91 Athens, J. S. & Leppard, T. P. Settlement and Subsistence in the Remote Western Pacific: Archaeological and Radiocarbon Data from Alamagan, Northern Mariana Islands. *Journal of Field Archaeology* **44**, 109-125, doi:10.1080/00934690.2019.1571856 (2019).

92 Shell, R. J. The Marianas Population Decline: 17th Century Estimates. *The Journal of Pacific History* **34**, 291-305 (1999).

93 Carrell, T. *Maritime history and archaeology of the Commonwealth of the Northern Mariana Islands*. (Ships of Exploration and Discovery Research, 2009).

94 Conry, P. J. *Ecology of the wild (feral) pig (Sus scrofa) on Guam: . Technical Report. .* (Guam Division of Aquatic and Wildlife Resources, Guam Department of Agriculture, 1989).

95 Wiles, G. J., Budend, D. W. & Worthington, J. History of introduction, population status, and management of Philippine deer (Cervus mariannus) on Micronesian Islands. *Mammalia* **63**, 193-215 (1999).

96 Gawel, A. M., Rogers, H. S., Miller, R. H. & Kerr, A. M. Contrasting ecological roles of non-native ungulates in a novel ecosystem. *R Soc Open Sci* **5**, 170151, doi:10.1098/rsos.170151 (2018).

97 Olson, D. M. *et al.* Terrestrial Ecoregions of the World: A New Map of Life on Earth: A new global map of terrestrial ecoregions provides an innovative tool for conserving biodiversity. *BioScience* **51**, 933-938, doi:10.1641/0006-3568(2001)051[0933:Teotwa]2.0.Co;2 (2001).

98 Athens, J. S. & Ward, J. V. *Palau Compact Road, Archaeological Investigations, Babeldaob Island, Republic of Palau. Phase I: Intensive Archaeological Survey. Report Prepared for the US Army Engineer District, Honolulu, Ft. Shafter, Hawaia*. Vol. IV: Holocene Paleoenvironment and Landscape Change (International Archaeological Research Institute, 2005).

99 Dickinson, W. R. & Athens, J. S. Holocene Paleoshoreline and Paleoenvironmental History of Palau: Implications for Human Settlement. *The Journal of Island and Coastal Archaeology* **2**, 175-196, doi:10.1080/15564890701623639 (2007).

100 Welch, D. J. Archaeological and Palaeoenvironmental Evidence of Early Settlement in Palau. *Indo-Pacific Prehistory Association Bulletin 22* **Melake Papers, Volume 6**, 161-173 (2002).

101 Clark, G., Anderson, A. & Wright, D. Human Colonization of the Palau Islands, Western Micronesia. *The Journal of Island and Coastal Archaeology* **1**, 215-232, doi:10.1080/15564890600831705 (2006).

102 Clarke, G. R. A 3000-Year Culture Sequence for Palau, Western Micronesia. *Asian Perspectives* **44** (2005).

103 Denham, T. Early farming in Island Southeast Asia: an alternative hypothesis. *Antiquity* **87**, 250-257 (2013).

104 Masse, W. B., Liston, J., Carucci, J. & Athens, J. S. Evaluating the effects of climate change on environment, resource depletion, and culture in the Palau Islands between AD 1200 and 1600. *Quaternary International* **151**, 106-132, doi:10.1016/j.quaint.2006.01.017 (2006).

105 Farley, G., Schneider, L., Clark, G. & Haberle, S. G. A Late Holocene palaeoenvironmental reconstruction of Ulong Island, Palau, from starch grain, charcoal, and geochemistry analyses. *Journal of Archaeological Science: Reports* **22**, 248-256, doi:10.1016/j.jasrep.2018.09.024 (2018).

106 Clark, G. & Reepmeyer, C. Last millennium climate change in the occupation and abandonment of Palau's Rock Islands. *Archaeol. Oceania* **47**, 29–38 (2012).

107 Gorenflo, L. J. Demographic Change in the Republic of Palau. *Pacific Studies* **19**, 37-106 (1996).

108 Semper, K. *The Palau Islands in the Pacific Ocean (translated by Berg, M).* (Micronesian Area Research Center, University of Guam, 1982).

109 Callaghan, R. & Fitzpatrick, S. M. On the Relative Isolation of a Micronesian Archipelago during the Historic Period: the Palau Case-Study. *International Journal of Nautical Archaeology* **36**, 353-364, doi:10.1111/j.1095-9270.2007.00147.x (2007).

110 Force, R. W. Leadership and Cultural Change in Palau. *Fieliana Anthropology* **50** (1960).

111 Fenner, F., Henderson, D. A., Arite, I., Ježek, Z. & Ladnyi, I. D. in *Smallpox and its Eradication* 209-244 (World Health Organisation, 1988).

112 Francis, X. & Hezel, S. J. Disease in Micronesia: A Historical Survey. *Pacific Health Dialogue* **16**, 11-25 (2010).

113 Hughes, J. D. Palau: A parable for the twenty-first century. *Capitalism Nature Socialism* **16**, 85-88, doi:10.1080/10455750500108336 (2006).

114 Staver, A. C., Archibald, S. & Levin, S. A. The Global Extent and Determinants of Savanna and Forest as Alternative Biome States. *Science* **334**, 230-232 (2011).

115 Olson, D. M. *et al.* Terrestrial Ecoregions of the World: A New Map of Life on Earth. *BioScience* **51**, 933-938, doi:10.1641/0006-3568(2001)051[0933:TEOTWA]2.0.CO (2001).

116 Napolitano, M. F., Fitzpatrick, S. M., Clark, G. & Stone, J. H. New Investigations of Early Prehistoric Settlement on Yap, Western Caroline Islands. *The Journal of Island and Coastal Archaeology* **14**, 101-107, doi:10.1080/15564894.2017.1335661 (2017).

117 Hunt, E. E., Kidder, N. R. & Schneider, D. M. The Depopulation of Yap. *Human Biology* **26**, 21-51 (1954).

118 Hunter-Anderson, R. L. A Review of Traditional Micronesian High Island Horticulture in Belau, Yap, Chuuk, Pohnpei, and Kosrae. *Micronesica* **24**, 1-56 (2001).

119 Masse, W. B. Radiocarbon dating, sea-level change and the peopling of Belau. *Micronesica* **Supplement 2**, 213–230 (1990).

120 Gorenflo, L. J. Regional Demographic Change in Yap State, Federated States of Micronesia. *Pacific Studies* **14** (1991).

121 Ward, J. V. Palynology of Kosrae, Eastern Caroline Islands: Recoveries from pollen rain and holocene deposits. *Review of Palaeobotany and Palynology* **55**, 247-271, doi:https://doi.org/10.1016/0034-6667(88)90089-9 (1988).

122 Hamilton, R., Penny, D. & Hua, Q. A 4700-year record of hydroclimate variability over the Asian monsoon intersection zone inferred from multi-proxy analysis of lake sediments. *Global and Planetary Change* **174**, 92-104, doi:10.1016/j.gloplacha.2018.12.009 (2019).

123 Athens, J. S., Ward, J. V. & Murakami, G. M. Development of an agroforest on a Micronesian high island: prehistoric Kosraean agriculture. *Antiquity* **70**, 834-846, doi:10.1017/S0003598X00084106 (1996).

124 Athens, J. S. & Stevenson, J. Pohnpei Coring Records: The Natural Distribution of Cyrtosperma chamissonis and Neolithic Charcoal Particles. *Journal of Pacific Archaeology* **3**, 35-48 (2012).

125 Kirch, P. V. *On the road of the winds: an archeological history of the Pacific Islands before European contact*. (University of California Press, 2000).

126 Athens, J. S. *Landscape archaeology: prehistoric settlement, subsistence, and environment of Kosrae, eastern Caroline Islands, Micronesia.* . (International Archaeological Research Institute, 1995).

127 Haun, A. E. *Prehistoric Subsistence, Population, and Sociopolitical Evolution on Ponape, Micronesia,* Ph.D thesis, University of Oregon, (1984).

128 Ayers, W. S. & Haun, A. E. in *Pacific Production Systems: Approaches to Economic Prehistory Australian National University Research School for Pacific Studies Occasional Pacific Studies Occasional Paper 18* (eds D. Yen & M.J.M. Mummery) 211-227 (Australian National University Press, 1990).

129 Seikel, K. S. Mortuary contexts and social structure at Nan Madol, Pohnpei. . *Journal of Island and Coastal Archaeology* **6**, 442e460. (2011).

130 Ayers, W. S., Seikel, K. S. & Levin, M. Archaeological Remains at Angeir-Karian, Nan Madol, Pohnpei, Federated States of Micronesia, with Supplemental Studies at Sokehs and Temwen, Pohnpei. (Pohnpei State Historic Preservation Program, Pohnpei, 2009).

131 United States Office of the Chief of Naval Operations. *West Caroline Islands*. (Office of the chief of naval operations, Navy Department, 1943).

132 Gorenflo, L. J. Demographic change in Kosrae state, Federated States of Micronesia. *Pacific Studies* **16**, 67-118 (1993).

133 Levin, M. J. & Ayres, W. S. Managed agroforests, swiddening, and the introduction of pigs in Pohnpei, Micronesia: Phytolith evidence from an anthropogenic landscape. *Quaternary International* **434**, 70-77, doi:10.1016/j.quaint.2015.12.027 (2017).

134 Horrocks, M. & Weisler, M. I. Analysis of Plant Microfossils in Archaeological Deposits from Two Remote Archipelagos: The Marshall Islands, Eastern Micronesia, and the Pitcairn Group, Southeast Polynesia. *Pacific Science* **60**, 261-280, doi:10.1353/psc.2006.0004 (2006).

135 Beardsley, F. R. *Archaeological investigations on Kwajalein Atoll, Marshall Islands*. (International Archaeological Research Institute, 1994).

136 Yamaguchi, T., H. Kayanne & Yamano, H. Archaeological Investigation of the Landscape History of an Oceanic Atoll: Majuro, Marshall Islands. *Pacific Science* **63**, 537-565 (2009).

137 Kayanne, H., Yasukochi, T., Yamaguchi, T., Yamano, H. & Yoneda, M. Rapid settlement of Majuro Atoll, central Pacific, following its emergence at 2000 years CalBP. *Geophysical Research Letters* **38**, n/a-n/a, doi:10.1029/2011gl049163 (2011).

138 Piperno, D. R., Ranere, A. J., Holst, I., Iriarte, J. & Dickau, R. Starch grain and phytolith evidence for early ninth millennium BP maize from the Central Balsas River Valley, Mexico. *Proceedings of the National Academy of Sciences* **106**, 5019-5024 (2009).

139 Kistler, L. *et al.* Multiproxy evidence highlights a complex evolutionary legacy of maize in South America. *Science* **362**, 1309, doi:10.1126/science.aav0207 (2018).

140 Smith, B. D. Reconsidering the Ocampo Caves and the Era of Incipient Cultivation in Mesoamerica. *Latin American Antiquity* **8**, 342-383, doi:10.2307/972107 (1997).

141 MacNeish, R. S. Ancient Mesoamerican Civilization. *Science* **143**, 531-537 (1964).

142 Watts, W. A. & Bradbury, J. P. Paleoecological studies at Lake Patzcuaro on the west-central Mexican Plateau and at Chalco in the Basin of Mexico. *Quaternary Research* **17**, 56-70 (1982).

143 Kaplan, L. in *Environment and Subsistence: The Prehistory of the Tehuacán Valley* (ed D. Byers) 201-211 (University of Texas Press, 1967).

144 Whitmore, T. M. & Turner, B. L. Landscapes of Cultivation in Mesoamerica on the Eve of the Conquest. *Annals of the Association of American Geographers* **82**, 402-425 (1992).

145 Park, J., Byrne, R., Böhnel, H., Garza, R. M. & Conserva, M. Holocene climate change and human impact, central Mexico: a record based on maar lake pollen and sediment chemistry. *Quaternary Science Reviews* **29**, 618-632, doi:10.1016/j.quascirev.2009.10.017 (2010).

146 Byrne, A. R. & Horn, S. P. Prehistoric agriculture and forest clearance in the Sierra de los Tuxtlas, Veracruz, Mexico. *Palynology* **13**, 181-193 (1989).

147 Richards, J. F. *The Unending Frontier : An Environmental History of the Early Modern World*. (University of California Press, 2003).

148 Conserva, M. E. & Byrne, R. Late Holocene Vegetation Change in the Sierra Madre Oriental of Central Mexico. *Quaternary Research* **58**, 122-129, doi:https://doi.org/10.1006/qres.2002.2348 (2002).

149 Arce, J. L. *et al.* Geology and stratigraphy of the Mexico Basin (Mexico City), central Trans-Mexican Volcanic Belt. *Journal of Maps* **15**, 320-332, doi:10.1080/17445647.2019.1593251 (2019).

150 Miller, S. W. *An Environmental History of Latin America*. (Cambridge University Press, 2007).

151 Pollard, H. P. in *Ruling "Purépecha Chichimeca" in a Tarascan World* (eds Sarah Kurnick & Joanne Baron) (University Press of Colorado, 2016).

152 Malmstrom, V. H. Geographical Origins of the Tarascans. *Geographical Review* **85**, 31-40, doi:10.2307/215553 (1995).
153 Turner, B. L., 2nd & Sabloff, J. A. Classic Period collapse of the Central Maya Lowlands: insights about human-environment relationships for sustainability. *Proc Natl Acad Sci U S A* **109**, 13908-13914, doi:10.1073/pnas.1210106109 (2012).
154 Jones, J. G. *Pollen evidence of prehistoric forest modification and Maya cultivation in Belize* PhD thesis, Texas A & M University, (1991).
155 Leyden, B. W. *et al.* in *The Managed Mosaic: Ancient Maya Agriculture and Resource Use.* (ed S.L. Fedick) (University of Utah Press, 1996).
156 Ford, A. in *The Maya World* (eds S.R. Hutson & T. Ardren) 519-539 (Routledge Worlds, 2020).
157 Abrams, E. M. & Rue, D. J. The Causes and Consequences of Deforestation among the Prehistoric Maya. *Human Ecology* **16**, 377-395 (1988).
158 McNeil, C. L., Burney, D. A. & Burney, L. P. Evidence disputing deforestation as the cause for the collapse of the ancient Maya polity of Copan, Honduras. *Proc Natl Acad Sci U S A* **107**, 1017-1022, doi:10.1073/pnas.0904760107 (2010).
159 Clement, R. M. & Horn, S. P. Pre-Columbian land-use history in Costa Rica: a 3000-year record of forest clearance, agriculture and fires from Laguna Zoncho. *The Holocene* **11**, 419-426, doi:10.1191/095968301678302850 (2001).
160 Castilla-Beltrán, A. *et al.* Columbus' footprint in Hispaniola: A paleoenvironmental record of indigenous and colonial impacts on the landscape of the central Cibao Valley, northern Dominican Republic. *Anthropocene* **22**, 66-80, doi:10.1016/j.ancene.2018.05.003 (2018).
161 Ortiz Aguilú, J. J., Rivera Meléndez, J., Principe Jácome, A., Meléndez Maiz, M. & Lavergne Colberg, M. Intensive Agriculture in Pre-Columbian West Indies: The Case for Terraces. (1991).
162 Mickleburgh, H. L. & Pagán-Jiménez, J. R. New insights into the consumption of maize and other food plants in the pre-Columbian Caribbean from starch grains trapped in human dental calculus. *Journal of Archaeological Science* **39**, 2468-2478, doi:10.1016/j.jas.2012.02.020 (2012).
163 Lane, C. S., Horn, S. P. & Orvis, K. H. The Earliest Evidence of Ostionoid Maize Agriculture from the Interior of Hispaniola. *Caribbean Journal of Science* **44**, 43-52, doi:10.18475/cjos.v44i1.a6 (2008).
164 van Hengstum, P. J. *et al.* The intertropical convergence zone modulates intense hurricane strikes on the western North Atlantic margin. *Sci Rep* **6**, 21728, doi:10.1038/srep21728 (2016).
165 Steadman, D. W. *et al.* Exceptionally well preserved late Quaternary plant and vertebrate fossils from a blue hole on Abaco, The Bahamas. *Proceedings of the National Academy of Sciences of the United States of America* **104**, 19897-19902, doi:10.1073/pnas.0709572104 (2007).
166 Higuera-Gundy, A. *Antillean vegetational history and paleoclimate reconstructed from the paleolimnological record of Lake Miragoane, Haiti* Ph.D. thesis, University of Florida, (1991).
167 Pons, F. M. The politics of forced Indian labour in La Española 1493–1520. *Antiquity* **66**, 130-139 (1992).
168 Currey, B. & Hugo, B. *Famine as a Geographical Phenomenon*. 202 (Springer, 1984).
169 Valcárcel Rojas, R., Laffoon, J. E., Weston, D. A., Hoogland, M. L. P. & Hofman, C. L. Slavery of Indigenous People in the Caribbean: An Archaeological Perspective. *International Journal of Historical Archaeology*, doi:10.1007/s10761-019-00522-x (2019).
170 Schwaller, R. C. Contested Conquests:African Maroons and the Incomplete Conquest of Hispaniola, 1519–1620. *The Americas* **75**, 609-638, doi:10.1017/tam.2018.3 (2018).
171 Gott, R. *Cuba: A new history*. (Yale University Press, 2004).

172 Fortes-Lima, C. *et al.* Exploring Cuba's population structure and demographic history using genome-wide data. *Sci Rep* **8**, 11422, doi:10.1038/s41598-018-29851-3 (2018).

173 Morsink, J. Spanish-Lucayan Interaction: Continuity of Native Economies in Early Historic Times. *Journal of Caribbean Archaeology* **15**, 102-119 (2015).

174 Maezumi, S. Y. *et al.* New Insights From Pre-Columbian Land Use and Fire Management in Amazonian Dark Earth Forests. *Frontiers in Ecology and Evolution* **6**, doi:10.3389/fevo.2018.00111 (2018).

175 Bush, M. B. *et al.* Anthropogenic influence on Amazonian forests in pre-history: An ecological perspective. *Journal of Biogeography* **42**, 2277-2288, doi:10.1111/jbi.12638 (2015).

176 Piperno, D. R., McMichael, C. & Bush, M. B. Amazonia and the Anthropocene: What was the spatial extent and intensity of human landscape modification in the Amazon Basin at the end of prehistory? *The Holocene* **25**, 1588-1597, doi:10.1177/0959683615588374 (2015).

177 Montoya, E., Lombardo, U., Levis, C., Aymard, G. A. & Mayle, F. E. in *Neotropical Diversification: Patterns and Processes Fascinating Life Sciences* Ch. Chapter 19, 495-520 (2020).

178 Maezumi, S. Y. *et al.* The legacy of 4,500 years of polyculture agroforestry in the eastern Amazon. *Nat Plants* **4**, 540-547, doi:10.1038/s41477-018-0205-y (2018).

179 Bush, M. B. *et al.* Holocene fire and occupation in Amazonia: records from two lake districts. *Philosophical Transactions of the Royal Society B: Biological Sciences* **362**, 209-218, doi:doi:10.1098/rstb.2006.1980 (2007).

180 Roosevelt, A. C. The Amazon and the Anthropocene: 13,000 years of human influence in a tropical rainforest. *Anthropocene* **4**, 69-87, doi:10.1016/j.ancene.2014.05.001 (2013).

181 Bush, M. B. *et al.* A 6900-year history of landscape modification by humans in lowland Amazonia. *Quaternary Science Reviews* **141**, 52-64, doi:10.1016/j.quascirev.2016.03.022 (2016).

182 Sombroek, W. G. *Amazon Soils*. (Centre for agricultural publications and documentation, 1966).

183 Iriarte, J. in *Beyond Waters: Archaeology and Environmental History of the Amazonian Inland* (ed P Stenborg) 71-86 (University of Gothenburg, 2016).

184 McMichael, C. H. *et al.* Predicting pre-Columbian anthropogenic soils in Amazonia. *Proc Biol Sci* **281**, 20132475, doi:10.1098/rspb.2013.2475 (2014).

185 de Souza, J. G. *et al.* Pre-Columbian earth-builders settled along the entire southern rim of the Amazon. *Nat Commun* **9**, 1125, doi:10.1038/s41467-018-03510-7 (2018).

186 McMichael, C. N. H. & Bush, M. B. Spatiotemporal patterns of pre-Columbian people in Amazonia. *Quaternary Research* **92**, 53-69, doi:10.1017/qua.2018.152 (2019).

187 Clement, C. R., McCann, J. M. & Smith, N. J. in *Amazonian dark earths* 159-178 (Springer, 2003).

188 Levis, C. *et al.* How people domesticated Amazonian forests. *Frontiers in Ecology and Evolution* **5**, 171 (2018).

189 Levis, C. *et al.* Persistent effects of pre-Columbian plant domestication on Amazonian forest composition. *Science* **355**, 925-931 (2017).

190 Watling, J. *et al.* Impact of pre-Columbian "geoglyph" builders on Amazonian forests. *Proc Natl Acad Sci U S A* **114**, 1868-1873, doi:10.1073/pnas.1614359114 (2017).

191 Piperno, D. R., McMichael, C. & Bush, M. B. Further evidence for localized, short-term anthropogenic forest alterations across pre-Columbian Amazonia. *Proceedings of the National Academy of Sciences* **114**, E4118-E4119, doi:10.1073/pnas.1705585114 (2017).

192 McMichael, C. H. *et al.* Sparse pre-Columbian human habitation in western Amazonia. *Science* **336**, 1429-1431 (2012).

193 Loughlin, N. J. D., Gosling, W. D., Mothes, P. & Montoya, E. Ecological consequences of post-Columbian indigenous depopulation in the Andean-Amazonian corridor. *Nat Ecol Evol* **2**, 1233-1236, doi:10.1038/s41559-018-0602-7 (2018).

194 Chambouleyron, R. *Colonial Portuguese Amazon Region, from the 17th to 18th Centuries*. (Oxford Bibliographies, 2018).

195 Sioli, H. in *The Amazon: Limnology and landscape ecology of a mighty tropical river and its basin* (ed Harald Sioli) 1-13 (Springer Netherlands, 1984).

196 Buchillet, D. Colonization and Epidemic Diseases in the Upper Rio Negro Region, Brazilian Amazon (Eighteenth-Nineteenth Centuries). *Boletín de Antropología* **33**, doi:10.17533/udea.boan.v33n55a06 (2018).

197 Balée, W. *Cultural Forests of the Amazon: A Historical Ecology of People and Their Landscapes*. (The University of Alabama Press, 2013).

198 Lombardo, U. *et al.* Early Holocene crop cultivation and landscape modification in Amazonia. *Nature* **581**, 190-193, doi:10.1038/s41586-020-2162-7 (2020).

199 Denevan, W. M. *Cultivated landscapes of native Amazonia and the Andes*. (Oxford University Press, 2002).

200 Denevan, W. M. "Pre-Spanish Earthworks in the Llanos de Mojos of Northeastern Bolivia". *Revista Geográfica* **33**, 17-25 (1964).

201 Carson, J. F., Mayle, F. E., Whitney, B. S., Iriarte, J. & Soto, J. D. Pre-Columbian ring ditch construction and land use on a 'chocolate forest island' in the Bolivian Amazon. *Journal of Quaternary Science* **31**, 337-347, doi:10.1002/jqs.2835 (2016).

202 Lombardo, U., Canal-Beeby, E. & Bern, H. V. Eco-archaeological regions in the Bolivian Amazon: An overview of pre-Columbian earthworks linking them to their environmental settings. *Swiss Journal of Geography* **3**, 173–182 (2011).

203 Lombardo, U., May, J.-H. & Veit, H. Mid-to late-Holocene fluvial activity behind pre-Columbian social complexity in the southwestern Amazon basin. *The Holocene* **22**, 1035-1045 (2012).

204 Walker, J. H. in *The Handbook of South American Archaeology* (eds Helaine Silverman & William H. Isbell) 927-939 (Springer New York, 2008).

205 Whitney, B. S. *et al.* Pre-Columbian raised-field agriculture and land use in the Bolivian Amazon. *The Holocene* **24**, 231-241, doi:10.1177/0959683613517401 (2014).

206 Erickson, C. in *The Handbook of South American Archaeology* (eds H. Silverman & W. H. Isbell) 157-183 (Springer, 2008).

207 Erickson, C. L. The transformation of environment into landscape: The historical ecology of monumental earthwork construction in the Bolivian Amazon. *Diversity* **2**, 618-652 (2010).

208 Carson, J. F. *et al.* Pre-Columbian land use in the ring-ditch region of the Bolivian Amazon. *The Holocene* **25**, 1285-1300, doi:10.1177/0959683615581204 (2015).

209 Carson, J. F. *et al.* Environmental impact of geometric earthwork construction in pre-Columbian Amazonia. *Proc Natl Acad Sci U S A* **111**, 10497-10502, doi:10.1073/pnas.1321770111 (2014).

210 Whitney, B. S., Dickau, R., Mayle, F. E., Soto, J. D. & Iriarte, J. Pre-Columbian landscape impact and agriculture in the Monumental Mound region of the Llanos de Moxos, lowland Bolivia. *Quaternary Research* **80**, 207-217, doi:10.1016/j.yqres.2013.06.005 (2013).

211 Brugger, S. O. *et al.* Long-term man–environment interactions in the Bolivian Amazon: 8000 years of vegetation dynamics. *Quaternary Science Reviews* **132**, 114-128, doi:10.1016/j.quascirev.2015.11.001 (2016).

212 Urrego, D. H. *et al.* Holocene fires, forest stability and human occupation in south-western Amazonia. *Journal of Biogeography* **40**, 521-533, doi:10.1111/jbi.12016 (2013).

213 Block, D. *Mission Culture on the Upper Amazon: Native Tradition, Jesuit Enterprise & Secular Policy in Moxos, 1660-1880*. (Lincoln and London, 1994).

214 Jones, J. C. *Conflict between whites and Indians on the llanos de Moxos, Beni Department* Ph.D. thesis, University of Florida, (1980).

215 Denevan, W. M. Estimating the Aboriginal Population of Latin America in 1492: Methodological Synthesis. *Publication Series (Conference of Latin Americanist Geographers)* **5**, 125-132 (1976).

216 Gassón, R. A. Orinoquia: The Archaeology of the Orinoco River Basin. *Journal of World Prehistory* **16**, 237-311, doi:10.1023/A:1020978518142 (2002).

217 Leal, A., Gassón, R., Behling, H. & Sánchez, F. Human-made fires and forest clearance as evidence for late Holocene landscape domestication in the Orinoco Llanos (Venezuela). *Vegetation History and Archaeobotany* **28**, 545-557, doi:10.1007/s00334-019-00713-w (2019).

218 Rostain, S. in *The Handbook of South American Archaeology* (eds Helaine Silverman & William H. Isbell) 217-233 (Springer New York, 2008).

219 Hooghiemstra, H. & Flantua, S. G. A. in *The Geology of Colombia: Volume 4 Quaternary.* (eds J. Gómez & A.O. Pinilla–Pachon) (Servicio Geológico Colombiano, Publicaciones Geológicas Especiales 38, 2019).

220 Berrio, J. C., Hooghiemstra, H., Behling, H., Botero, P. & Van der Borg, K. Late-Quaternary savanna history of the Colombian Llanos Orientales from Lagunas Chenevo and Mozambique: a transect synthesis. *The Holocene* **12**, 35-48 (2002).

221 Bacci, M. L. Venezuela's melting pot: 1500-1800. *Belo Horizonte* **34**, 199-221 (2017).

222 Ledru, M.-P. in *The Cerrados of Brazil Ecology and Natural History of a Neotropical Savanna* (eds Paulo S. Oliveira & Robert J. Marquis) 33-50 (Columbia University Press, 2002).

223 da Silva, C. M. The miracle of the Brazilian Cerrados as a juggernaut: soil, science, and national culture. *Hispanic Issues Series* **24**, 98-116 (2019).

224 Pearsall, D. M. in *The Handbook of South American Archaeology* (eds Helaine Silverman & William H. Isbell) 105-120 (Springer New York, 2008).

225 Pivello, V. R. The Use of Fire in the Cerrado and Amazonian Rainforests of Brazil: Past and Present. *Fire Ecology* **7**, 24-39, doi:10.4996/fireecology.0701024 (2011).

226 Posey, D. Indigenous Management of Tropical Forest Ecosystems: The Case of the Kayapó Indians of the Brazil Amazonia. *Agroforestry Systems* **3**, 139-158 (1985).

227 Araujo, A. G. M., Neves, W. A., Piló, L. B. & Atui, J. P. V. Holocene dryness and human occupation in Brazil during the "Archaic Gap". *Quaternary Research* **64**, 298-307, doi:https://doi.org/10.1016/j.yqres.2005.08.002 (2005).

228 Klink, C. A. & Moreira, A. G. in *The Cerrados of Brazil Ecology and Natural History of a Neotropical Savanna* (eds Paulo S. Oliveira & Robert J. Marquis) 69-88 (Columbia University Press, 2002).

229 De Oliveira, P. E., Raczka, M., McMichael, C. N. H., Pinaya, J. L. D. & Bush, M. B. Climate change and biogeographic connectivity across the Brazilian cerrado. *Journal of Biogeography* **47**, 396-407, doi:10.1111/jbi.13732 (2019).

230 Lauterjung, M. B. *et al.* Phylogeography of Brazilian pine (Araucaria angustifolia): integrative evidence for pre-Columbian anthropogenic dispersal. *Tree Genetics & Genomes* **14**, doi:10.1007/s11295-018-1250-4 (2018).

231 da Cunha, M. C. *História dos índios no Brasil*. (Editora Companhia das Letras, 1992).

232 Sandweiss, D. H. & Richardson, J. B. in *The Handbook of South American Archaeology* (eds Helaine Silverman & William H. Isbell) 93-104 (Springer New York, 2008).

233 Drennan, R. D. in *The Handbook of South American Archaeology* (eds Helaine Silverman & William H. Isbell) 381-403 (Springer New York, 2008).

234 Behling, H., Hooghiemstra, H. & Negret, A. J. Holocene History of the Chocó Rain Forest from Laguna Piusbi, Southern Pacific Lowlands of Colombia. *Quaternary Research* **50**, 300-308, doi:https://doi.org/10.1006/qres.1998.1998 (1998).

235 Kris, R. & Lane, M. D. Miners & Maroons: Freedom of the Pacific Coast of Colombia and Ecuador. *Cultural Survival Quarterly Magazine* (2001).

236 Leal, C. *Landscapes of Freedom: Building a Postemancipation Society in the Rainforests of Western Colombia*. (University of Arizona Press, 2018).

237 Acemoglu, D., García-Jimeno, C. & Robinson, J. A. Finding Eldorado: Slavery and long-run development in Colombia. *Journal of Comparative Economics* **40**, 534-564, doi:https://doi.org/10.1016/j.jce.2012.07.003 (2012).

238 <Cannon_2005.pdf>.

239 Leal, C. & Van Ausdal, S. *Landscapes of Freedom and Inequality: Environmental Histories of the Pacific and Caribbean Coasts of Colombia: desiguALdades.net Working Paper Series 58*. (International Research Network on Interdependent Inequalities in Latin America, 2013).

240 Erickson, C. L. Prehistoric landscape management in the Andean highlands: Raised field agriculture and its environmental impact. *Population and Environment* **13**, 285-300, doi:10.1007/BF01271028 (1992).

241 Etter, A., McAlpine, C. & Possingham, H. Historical Patterns and Drivers of Landscape Change in Colombia Since 1500: A Regionalized Spatial Approach. *Annals of the Association of American Geographers* **98**, 2-23, doi:10.1080/00045600701733911 (2008).

242 Zimmerer, K. S. & Bell, M. G. Time for change: The legacy of a Euro-Andean model of landscape versus the need for landscape connectivity. *Landscape and Urban Planning* **139**, 104-116, doi:10.1016/j.landurbplan.2015.02.002 (2015).

243 Wernke, S. *Negotiated Settlements: Andean Communities and Landscapes under Inka and Spanish Colonialism*. (University Press of Florida, 2013).

244 Williams, J. W. *et al.* The Neotoma Paleoecology Database, a multiproxy, international, community-curated data resource. *Quaternary Research* **89**, 156-177, doi:10.1017/qua.2017.105 (2018).

245 Flantua, S. G. A., Blaauw, M. & Hooghiemstra, H. Geochronological database and classification system for age uncertainties in Neotropical pollen records. *Climate of the Past* **12**, 387-414, doi:10.5194/cp-12-387-2016 (2016).

246 Bacon, A.-M. *et al.* Testing the savannah corridor hypothesis during MIS2: The Boh Dambang hyena site in southern Cambodia. *Quaternary International* **464**, 417-439, doi:10.1016/j.quaint.2017.10.047 (2018).

247 Chen, S.-H. *et al.* Late Holocene paleoenvironmental changes in subtropical Taiwan inferred from pollen and diatoms in lake sediments. *Journal of Paleolimnology* **41**, 315-327, doi:10.1007/s10933-008-9227-1 (2009).

248 Chen, S.-H. *et al.* Late Holocene paleoenvironmental changes in subtropical Taiwan inferred from pollen and diatoms in lake sediments. *Journal of Paleolimnology* **41**, 315-327, doi:10.1007/s10933-008-9227-1 (2008).

249 Blaauw, M. & Christen, J. A. Flexible Paleoclimate Age-Depth Models Using an Autoregressive Gamma Process. *Bayesian Analysis* **6**, 457-474, doi:10.1214/11-ba618 (2011).

250 R: A language and environment for statistical computing (R Foundation for Statistical Computing, Vienna, 2013).

251 Dam, R. A. C., Fluin, J., Suparan, P. & van der Kaars, S. Palaeoenvironmental developments in the Lake Tondano area (N. Sulawesi, Indonesia) since 33,000yr B.P. *Palaeogeography, Palaeoclimatology, Palaeoecology* **171**, 147-183, doi:https://doi.org/10.1016/S0031-0182(01)00244-9 (2001).

252 Athens, J. S. & Ward, J. V. Paleoenvironment and Prehistoric Landscape Change: A Sediment Core Record from Lake Hagoi, Tinian, CNMI. (Department of the Navy Pacific Division, Naval Facilities Engineering Command, Hawaii, 1998).

253 Athens, J. S. & Ward, J. V. Paleoenvironment of the Orote Penisula, Guam. *Micronesica* **28**, 51-76 (1995).

254 Athens, J. S. & Ward, J. V. Holocene Paleo-environment of Saipan: Analysis of a Core from Lake Susupe., (Division of Historic Preservation, Department of Community and Cultural Affairs, CNMI Saipan, Hawaii, 2004).

255 Ward, J. V. in *Archaeology in the Marine Drive Corridor from Agana to Agat, Guam* (eds E.F. Wells, U.K. Prasad, & R. Hunter-Anderson) Ch. Appendix B, 203-224 (Guam Department of Public Works, 1994).

256 Athens, J. S., Dega, M. F. & Ward, J. V. Austronesian Colonisation of the Mariana Islands: The palaeoenvironmental evidence. *Indo-Pacific Prehistory Association Bulletin* **24**, 21-30 (2004).

257 Athens, J. S. & Ward, J. V. Holocene Paleoenvironmental Investigations on Ngerekebesang, Koror, South Babeldaob, and Peleliu Islands, Palau. (Palau National Communications Corporation, Koror, Palau, Hawaii, 2002).

258 Dodson, J. R. & Intoh, M. Prehistory and palaeoecology of Yap, federated states of Micronesia. *Quaternary International* **59**, 17-26, doi:https://doi.org/10.1016/S1040-6182(98)00068-8 (1999).

259 Goman, M. & Byrne, R. A 5000-year record of agriculture and tropical forest clearance in the Tuxtlas, Veracruz, Mexico. *The Holocene* **8**, 83-89, doi:http://dx.doi.org/10.1191/095968398670396093 (1998).

260 Nunn, P. D. Sea-Level Changes over the past 1,000 Years in the Pacific. *Journal of Coastal Research* **14**, 23-30 (1998).

261 Rue, D. J. Early agriculture and early Postclassic Maya occupation in western Honduras. *Nature* **326**, 285-286, doi:10.1038/326285a0 (1987).

262 Taylor, Z. P., Horn, S. P. & Finkelstein, D. B. Pre-Hispanic agricultural decline prior to the Spanish Conquest in southern Central America. *Quaternary Science Reviews* **73**, 196-200, doi:10.1016/j.quascirev.2013.05.022 (2013).

263 Crausbay, S. D., Martin, P. H., Kelly, E. F. & McGlone, M. Tropical montane vegetation dynamics near the upper cloud belt strongly associated with a shifting ITCZ and fire. *Journal of Ecology* **103**, 891-903, doi:10.1111/1365-2745.12423 (2015).

264 Castilla-Beltrán, A. *et al.* Columbus' footprint: Land-use change before and after European incursion in Hispaniola. *Past Global Changes Magazine* **28**, 24-25 (2020).

265 Hooghiemstra, H. *et al.* Columbus' environmental impact in the New World: Land use change in the Yaque River valley, Dominican Republic. *Holocene* **28**, 1818-1835, doi:10.1177/0959683618788732 (2018).

266 Kennedy, L. M., Horn, S. P. & Orvis, K. H. A 4000-year record of fire and forest history from Valle de Bao, Cordillera Central, Dominican Republic. *Palaeogeography, Palaeoclimatology, Palaeoecology* **231**, 279-290, doi:10.1016/j.palaeo.2005.08.002 (2006).

267 Caffrey, M. A. & Horn, S. P. Long-Term Fire Trends in Hispaniola and Puerto Rico from Sedimentary Charcoal: A Comparison of Three Records. *The Professional Geographer* **67**, 229-241, doi:10.1080/00330124.2014.922017 (2015).

268 Higuera-Gundy, A. *et al.* A 10,300 14C yr Record of Climate and Vegetation Change from Haiti. *Quaternary Research* **52**, 159-170, doi:https://doi.org/10.1006/qres.1999.2062 (1999).

269 Kelly, T. J. *et al.* The vegetation history of an Amazonian domed peatland. *Palaeogeography, Palaeoclimatology, Palaeoecology* **468**, 129-141, doi:10.1016/j.palaeo.2016.11.039 (2017).

270 Bush, M. B., Silman, M. R. & Listopad, C. M. C. S. A regional study of Holocene climate change and human occupation in Peruvian Amazonia. *Journal of Biogeography* **34**, 1342-1356, doi:10.1111/j.1365-2699.2007.01704.x (2007).

271 Berrio, J. C., Hooghiemstra, H., Behling, H. & van der Borg, K. Late Holocene history of savanna gallery forest from Carimagua area, Colombia. *Review of Palaeobotany and Palynology* **111**, 295-308, doi:https://doi.org/10.1016/S0034-6667(00)00030-0 (2000).

272 Velez, M. I., Wille, M., Hooghiemstral, H. & Metcalfe, S. Integrated diatom-pollen based Holocene environmental reconstruction of lake Las Margaritas, eastern savannas of Colombia. *The Holocene* **15**, 1184-1198, doi:10.1191/0959683605hl890rp (2005).

273 Ledru, M.-P. Late Quaternary Environmental and Climatic Changes in Central Brazil. *Quaternary Research* **39**, 90-98, doi:10.1006/qres.1993.1011 (1993).

274 Behling, H. A high resolution Holocene pollen record from Lago do Pires, SE Brazil: vegetation, climate and fire history. *Journal of Paleolimnology* **14**, 253-268, doi:10.1007/BF00682427 (1995).

275 Behling, H. Late Quaternary vegetation, climate and fire history from the tropical mountain region of Morro de Itapeva, SE Brazil. *Palaeogeography, Palaeoclimatology, Palaeoecology* **129**, 407-422, doi:https://doi.org/10.1016/S0031-0182(97)88177-1 (1997).

276 Behling, H. Late Quaternary vegetation, climate and fire history of the Araucaria forest and campos region from Serra Campos Gerais, ParanáState (South Brazil). *Review of Palaeobotany and Palynology* **97**, 109-121, doi:https://doi.org/10.1016/S0034-6667(96)00065-6 (1997).

277 Behling, H. Investigations into the late Pleistocene and Holocene history of vegetation and climate in Santa Catarina (S Brazil). *Vegetation History and Archaeobotany* **4**, 127-152, doi:10.1007/BF00203932 (1995).

278 Vélez, M. I. *et al.* Late Holocene environmental history of southern Chocó region, Pacific Colombia; sediment, diatom and pollen analysis of core El Caimito. *Palaeogeography, Palaoclimatology, Palaeoecology* **173**, 197-214. (2001).

279 Lim, S. *et al.* Ecological effects of natural hazards and human activities on the Ecuadorian Pacific coast during the late Holocene. *Palaeogeography, Palaeoclimatology, Palaeoecology* **415**, 197-209, doi:10.1016/j.palaeo.2013.12.021 (2014).

280 Urrego, L. E., Molina, L. A., Urrego, D. H. & Ramírez, L. F. Holocene space–time succession of the Middle Atrato wetlands, Chocó biogeographic region, Colombia. *Palaeogeography, Palaeoclimatology, Palaeoecology* **234**, 45-61, doi:https://doi.org/10.1016/j.palaeo.2005.10.018 (2006).

281 Berrío, J. C., Hooghiemstra, H., Marchant, R. & Rangel, O. Late-glacial and Holocene history of the dry forest area in the south Colombian Cauca Valley. *Journal of Quaternary Science* **17**, 667-682 (2002).

282 Behling, H., Negret, A. J. & Hooghiemstra, H. Late Quaternary vegetational and climatic change in the Popayán region, southern Colombian Andes. *Journal of Quaternary Science* **13**, 43-53, doi:10.1002/(SICI)1099-1417(199801/02)13:1<43::AID-JQS348>3.0.CO;2-G (1998).

283 Wille, M., Hooghiemstra, H., Behling, H., van der Borg, K. & Negret, A. J. Environmental change in the Colombian subandean forest belt from 8 pollen records: the last 50 kyr. *Vegetation History and Archaeobotany* **10**, 61-77 (2001).

284 Vélez, M. I., Berrío, J. C., Hooghiemstra, H., Metcalfe, S. & Marchant, R. Palaeoenvironmental changes during the last ca. 8590 calibrated yr (7800 radiocarbon yr) in the dry forest ecosystem of the Patía Valley, Southern Colombian Andes: a multiproxy approach. *Palaeogeography, Palaeoclimatology, Palaeoecology* **216**, 279-302, doi:10.1016/j.palaeo.2004.11.006 (2005).

285 Epping, I. *Environmental change in the Colombian upper forest belt* Masters thesis, University of Amsterdam, (2009).

286 Niemann, H. & Behling, H. Late Pleistocene and Holocene environmental change inferred from the Cocha Caranga sediment and soil records in the southeastern Ecuadorian Andes. *Palaeogeography, Palaeoclimatology, Palaeoecology* **276**, 1-14, doi:10.1016/j.palaeo.2009.02.018 (2009).

287 Napolitano, M. F. *et al.* Reevaluating human colonization of the Caribbean using chronometric hygiene and Bayesian modeling. *Science Advances* **5**, eaar7806, doi:10.1126/sciadv.aar7806 (2019).

288 Velásquez-R, C. A. & Hooghiemstra, H. Pollen-based 17-kyr forest dynamics and climate change from the Western Cordillera of Colombia; no-analogue associations and temporarily lost biomes. *Review of Palaeobotany and Palynology* **194**, 38-49, doi:10.1016/j.revpalbo.2013.03.001 (2013).

289 Rull, V., Salgado-Labouriau, M.-L., Schubert, C. & Valastro, S., Jr. . Late Holocene temperature depression in the Venezuelan Andes: palynological evidence. *Palaeogeography, Palaeoclimatology, Palaeoecology* **60**, 109-121 (1987).

290 Graf, K. *Pollen diagramme aus den Anden, eine Synthese zur Klimageschichte und Vegetationsentwicklung seit der letzten Eiszeit.*, Vol. 34 138 (University of Zurich, 1992).

291 Kuhry, P., Salomons, J. B., Riezebos, P. A. & Van der Hammen, T. in *La Cordillera Central Colombiana transecto Parque Los Nevados Studies on Tropical Andean Ecosystems/Estudios de Ecosistemas Tropandinos* (eds T. van der Hammen, P.A. Perez, & P. Pinto) 227-261 (Cramer, 1983).

292 van der Hammen, T. Palinologia de la region de "Laguna de los Bobos": Historia de su clima, vegetacion y agricultura durante los ultimos 5.000 años. Revista de la Academia Colombiana de Ciencias Exactas. *Físicas y Naturales* **11**, 359-361 (1962).

293 Goldberg, W. M. *The Geography, Nature and History of the Tropical Pacific and Its Islands.* (Springer, 2017).

294 Pagán-Jiménez, J. R., Rodríguez-Ramos, R., Reid, B. A., van den Bel, M. & Hofman, C. L. Early dispersals of maize and other food plants into the Southern Caribbean and Northeastern South America. *Quaternary Science Reviews* **123**, 231-246, doi:10.1016/j.quascirev.2015.07.005 (2015).
