# Supplementary material for: Non-uniform tropical forest responses to the ‘Columbian Exchange’ in the Neotropics and Asia-Pacific
Source: Nat Ecol Evol. 2021 Jun 10;5(8):1174–84. doi: 10.1038/s41559-021-01474-4 (PMC8324576; doi:10.1038/s41559-021-01474-4)
Supplement: Supplementary file 2 — Reporting Summary [file 41559_2021_1474_MOESM2_ESM.pdf]

nature research

Corresponding author(s): Rebecca Hamilton
Patrick Roberts

Last updated by author(s): Mar 30, 2021

Reporting Summary

Nature Research wishes to improve the reproducibility of the work that we publish. This form provides structure for consistency and transparency in reporting. For further information on Nature Research policies, see our Editorial Policies and the Editorial Policy Checklist.

Statistics

For all statistical analyses, confirm that the following items are present in the figure legend, table legend, main text, or Methods section.

n/a	Confirmed	
☐	☒	The exact sample size (*n*) for each experimental group/condition, given as a discrete number and unit of measurement
☐	☒	A statement on whether measurements were taken from distinct samples or whether the same sample was measured repeatedly
☐	☒	The statistical test(s) used AND whether they are one- or two-sided<br>*Only common tests should be described solely by name; describe more complex techniques in the Methods section.*
☐	☒	A description of all covariates tested
☐	☒	A description of any assumptions or corrections, such as tests of normality and adjustment for multiple comparisons
☒	☐	A full description of the statistical parameters including central tendency (e.g. means) or other basic estimates (e.g. regression coefficient) AND variation (e.g. standard deviation) or associated estimates of uncertainty (e.g. confidence intervals)
☒	☐	For null hypothesis testing, the test statistic (e.g. *F*, *t*, *r*) with confidence intervals, effect sizes, degrees of freedom and *P* value noted<br>*Give P values as exact values whenever suitable.*
☒	☐	For Bayesian analysis, information on the choice of priors and Markov chain Monte Carlo settings
☒	☐	For hierarchical and complex designs, identification of the appropriate level for tests and full reporting of outcomes
☒	☐	Estimates of effect sizes (e.g. Cohen's *d*, Pearson's *r*), indicating how they were calculated

Our web collection on statistics for biologists contains articles on many of the points above.

Software and code

Policy information about availability of computer code

Data collection	All data collection databases/software are open access as specified below:<br>- All Spanish American pollen datasets were extracted from The Neotoma Paleoecology Database https://www.neotomadb.org/<br>- 4 Asia-Pacific charcoal datasets were extracted from the Global Paleofire database https://www.paleofire.org/<br>- Other pollen, charcoal and phytolith data from the Asia Pacific were extracted using Web Plot Digitiser 4.4
Data analysis	All analytical software is open access as specified below:<br>- Reworked chronological models were run in the R program Bacon (2.5.0)<br>- Implementation of the GAM fits, calculation of uncertainty intervals, and creation of core-specific GAM plots was undertaken in R version 3.6.2 using the “mgcv” package version 1.8-31. Data import and manipulation used the “data.table” version 1.12.8 and “readxl” version 1.3.1 packages.<br>- Cluster analysis of the Spanish American plant functional group data was conducted in R version 3.6.2 using the 'rioja' package 0.9-21

For manuscripts utilizing custom algorithms or software that are central to the research but not yet described in published literature, software must be made available to editors and reviewers. We strongly encourage code deposition in a community repository (e.g. GitHub). See the Nature Research guidelines for submitting code & software for further information.

Data

Policy information about availability of data

All manuscripts must include a data availability statement. This statement should provide the following information, where applicable:

- Accession codes, unique identifiers, or web links for publicly available datasets
- A list of figures that have associated raw data
- A description of any restrictions on data availability

The synthesised datasets used to undertake this analyses and analytical script used to create core-specific plots is available at the following OSF project page: https://osf.io/gu483/. This also includes code to apply the same methods used in this paper to other datasets (DOI 10.17605/OSF.IO/GU483).

Field-specific reporting

Please select the one below that is the best fit for your research. If you are not sure, read the appropriate sections before making your selection.

☐ Life sciences ☐ Behavioural & social sciences ☒ Ecological, evolutionary & environmental sciences

For a reference copy of the document with all sections, see nature.com/documents/nr-reporting-summary-flat.pdf

Ecological, evolutionary & environmental sciences study design

All studies must disclose on these points even when the disclosure is negative.

Study description

We compile and synthesis pollen data (Spanish Americas), and pollen, phytolith and charcoal data (Spanish East Indies) from available palaeoenvironmental records over the past 2,000 years to determine how tropical forests in the former Iberian Empire responded to land-use change associated with Indigenous population decline and European colonization. Specifically, we test the degree to which a uniform, pan-tropical 'anthropocene' process is visible following Iberian colonization, and assess how interplays of physical and human geography may complicate, or even overprint this signal in ecosystem dynamics.

Research sample

Due to the inaccessibility of raw palynological data from sites in the Spanish East Indies relative to the Spanish Americas, palaeoecological data from each of these regions were extracted and prepared differently as described below.

We extracted all pollen datasets made available for the Spanish Americas from The Neotoma Paleoecology Database (Neotoma). These were refined to those relevant for reconstructing tropical floristic change in the former Spanish Americas prior to-, at-, and after-Spanish colonisation using the sampling strategy outlined in the below "sampling strategy" section. We did not examine charcoal data from the Spanish Americas as this has already been completed in previous, recent research.

No pollen records, and only four relevant charcoal records from the Spanish East Indies have been made available through the Neotoma and the Global Paleofire Databases. We thus obtained additional charcoal and pollen records from the region using the following methods:
1) We obtained an unpublished raw charcoal dataset prepared by J. Stevenson (coauthor) from a site (Lake Bulalacao) in the Philippines, and a raw pollen dataset from Lake Paoay in the Philippines. The preparation of these data follows standard methods for calculating charcoal influx and the methods applied to the Neotropical pollen datasets extracted for assessment of Spanish American vegetation change.
2) In order to increase data capture within the Spanish East Indies, a review of regional pollen and phytolith publications was conducted. Data from any plots made of grass or dry herb data plotted against depth (in most cases used as a proxy for landscape openness), and any complimentary charcoal data was extracted using Web Plot Digitiser. While all efforts were made to ensure precise data extraction, minor sample or variable offsets may have been introduced as errors into the dataset depending on the quality of the initial graph production. Because some of these plots were made against depth (versus age), and interpretation of age was based on outdated chronologies, updated age-depth model for several of the cores was constructed using the program Bacon.

Sampling strategy

Because of the paucity of data from the Spanish East Indies relative to the Spanish Americas, and the availability of complete raw datasets for the Spanish Americans versus the Spanish East Indies, the sampling strategy (i.e. selection for inclusion and data preparation) varies between the two region as described below:

Spanish Americas
Pollen records were included in the study if:
1) The record was directly dated
2) The record encompasses the time period spanning at least 600 to 1900 CE, permitting reasonable assessment of the scale of Spanish induced change relative to the past 2,000 years.
3) The record derives from terrestrial sites that currently occur within a tropical or subtropical biome. If in a 'montane grassland, savanna and shrubland' biome, the site is proximal (<5km) to a tropical or subtropical biome.
4) The record includes at least one sample that is estimated to come from the time frame 1500 to 1600 CE, thereby permitting assessment of floristic response to any Spanish-induced land use change;
5) The temporal resolution of the upper 2000 years (or total core length where the base of the record is <2000 cal. BP), is <200 years per sample. This cutoff was set in an attempt to capture forest turnover while maintaining a reasonable distribution of records across study area. One record - Cobweb Swamp (Sawgrass Core) - which has a resolution of 212 years per sample, was retained for analysis as it is situated within the heart of urban development across the Mesoamerican lowlands during the Classic Maya period.
The application of the above criteria resulted in final selection of 28 pollen records.

	Spanish East Indies<br>Records were included if they captured environmental change within the 200-year period prior to Spanish/Portuguese contact (or known disease-influenced population decline), and at least one post Spanish imperial sample. The extracted data totaled 10 pollen, eight charcoal, and one phytolith record from 13 sites.
Data collection	RH collected and synthesised all of the data from Neotoma, published pollen records, and the Paleofire database as described in "Research sample".
Timing and spatial scale	Temporal framework: two-thousand years was set as an appropriate timeframe for assessing ecological dynamics as is short enough to identify late Holocene-scale floristic change, while being long enough to assess the magnitude of Spanish-influenced change against the backdrop of pre-Spanish land-use dynamics and low-magnitude late Holocene climate forcing (e.g. the Medieval Warm Period the Little Ice Age).<br>Spatial scale: All records from the area occupied by the former Spanish Empire were included in the analysis, and then refined following the "sampling strategy" detailed above.
Data exclusions	Data exclusions were systematic, and only occurred where the records did not meet the criteria for inclusion, and detailed in the "sampling strategy" above.
Reproducibility	Note that the project uses the entire population of the available palaeoecological datasets available for the study region. All data from the Neotropics were systematically reclassified following Marchant (2009) to eliminate uncertainty in assigning functional grouping to individual pollen taxa (a common issue in palynological analysis). The results produced from our analyses should be entirely reproducible if reanalysed.
Randomization	Note that the project uses the entire population of the available palaeoecological datasets available for the study region.<br>Randomisation is part of the statistical assessment of the individual records using Generalised Additive Models (GAMs). Specifically "GAMs use automatic smoothness selection methods to objectively determine the complexity of the fitted trend, and as formal statistical models, GAMs, allow for potentially complex, non-linear trends, a proper accounting of model uncertainty, and the identification of periods of significant temporal change... (As an) underlying mechanism of GAMs... the first derivatives of the trend are used to properly account for model uncertainty and identify periods of change." (Simpson, 2018, FREE. 6:149).
Blinding	Blinding is not relevant to this study as the project included the entire population of available palaeoecological data for the specified region.

Did the study involve field work? ☐ Yes ☒ No

Reporting for specific materials, systems and methods

We require information from authors about some types of materials, experimental systems and methods used in many studies. Here, indicate whether each material, system or method listed is relevant to your study. If you are not sure if a list item applies to your research, read the appropriate section before selecting a response.

Materials & experimental systems

n/a	Involved in the study
☒	☐ Antibodies
☒	☐ Eukaryotic cell lines
☒	☐ Palaeontology and archaeology
☒	☐ Animals and other organisms
☒	☐ Human research participants
☒	☐ Clinical data
☒	☐ Dual use research of concern

Methods

n/a	Involved in the study
☒	☐ ChIP-seq
☒	☐ Flow cytometry
☒	☐ MRI-based neuroimaging
